# Reversible Dual-Covalent Molecular Locking of the 14-3-3/ERRγ Protein–Protein Interaction as a Molecular Glue Drug Discovery Approach

**DOI:** 10.1021/jacs.2c12781

**Published:** 2023-03-16

**Authors:** Bente A. Somsen, Rick J.C. Schellekens, Carlo J.A. Verhoef, Michelle R. Arkin, Christian Ottmann, Peter J. Cossar, Luc Brunsveld

**Affiliations:** † Laboratory of Chemical Biology, Department of Biomedical Engineering, Institute for Complex Molecular Systems, Eindhoven University of Technology, P.O. Box 513, 5600 MB Eindhoven, The Netherlands; ‡ Department of Pharmaceutical Chemistry and Small Molecule Discovery Centre (SMDC), University of California, San Francisco, California 94143, United States

## Abstract

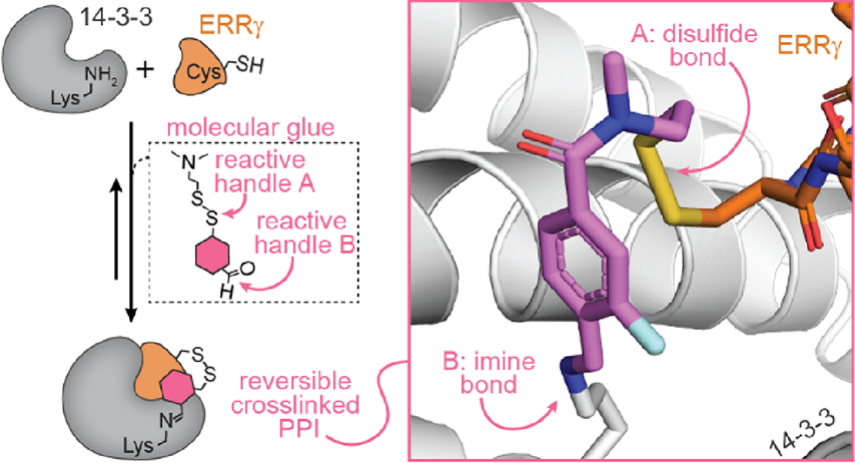

Molecules that stabilize protein–protein interactions (PPIs) are invaluable as tool compounds for biophysics and (structural) biology, and as starting points for molecular glue drug discovery. However, identifying initial starting points for PPI stabilizing matter is highly challenging, and chemical optimization is labor-intensive. Inspired by chemical crosslinking and reversible covalent fragment-based drug discovery, we developed an approach that we term “molecular locks” to rapidly access molecular glue-like tool compounds. These dual-covalent small molecules reversibly react with a nucleophilic amino acid on each of the partner proteins to dynamically crosslink the protein complex. The PPI between the hub protein 14-3-3 and estrogen-related receptor γ (ERRγ) was used as a pharmacologically relevant case study. Based on a focused library of dual-reactive small molecules, a molecular glue tool compound was rapidly developed. Biochemical assays and X-ray crystallographic studies validated the ternary covalent complex formation and overall PPI stabilization via dynamic covalent crosslinking. The molecular lock approach is highly selective for the specific 14-3-3/ERRγ complex, over other 14-3-3 complexes. This selectivity is driven by the interplay of molecular reactivity and molecular recognition of the composite PPI binding interface. The long lifetime of the dual-covalent locks enabled the selective stabilization of the 14-3-3/ERRγ complex even in the presence of several other competing 14-3-3 clients with higher intrinsic binding affinities. The molecular lock approach enables systematic, selective, and potent stabilization of protein complexes to support molecular glue drug discovery.

## Introduction

Covalent tool compounds are instrumental in investigating protein function, proteomics, drug discovery, and protein visualization.^1−3^ Molecules that react covalently with a protein polypeptide chain enable the study of molecular mechanisms typically not addressable using early-stage, noncovalent chemical matter. For instance, chemical crosslinkers,^4^ genetically encoded reactive handles,^5,6^ and photo^7^/chemical^8,9^-affinity labels have provided a deep mechanistic understanding of the proteins under study. Moreover, covalent chemical probes, tethered fragment libraries,^10−12^ ligand-directed cargo release,^13^ and covalent targeted protein degraders^14,15^ have enabled the study of disease states and have rapidly empowered drug discovery.

Molecular glues have also emerged as a powerful chemical biology concept and drug discovery innovation. These small molecules stabilize protein complexes leading to enhanced protein complex avidity. Molecular glues like Rapamycin,^16,17^ IMiDs,^18−20^ dCeMMs,^21^ and EN450^22^ have provided a greater understanding of protein–protein interaction (PPI) regulatory mechanisms and disease dysregulation.^23^ Moreover, native PPI stabilizers like NRX-103094^24^ and Trametiglue^25^ stand as compelling examples of the potential of native protein complex stabilization. Notwithstanding, the current paucity of molecular glues and of conceptual approaches to identify molecular starting points for their development highlights the significant challenges in designing “glue-like” chemical probes. This challenge at least partially originates from a lack of native ligands and the dynamic nature of multicomponent protein complexes. Consequently, covalent tool compounds could provide entries to study and “drug” a greater scope of PPIs.

Covalent molecular glues EN450^22^ and RM-18,^26,27^ which stabilize the UBE2D/NFκB and KRAS^G12C^/CYPA protein complexes, respectively, demonstrate the promising role of reactive handles in molecular glue tool compound design. Covalent chemical probes react specifically with a nucleophilic amino acid on a protein surface using an electrophilic moiety, such as an acrylamide or β-haloketone group. This is beneficial as the off-rate of small-molecule binding is reduced, enhancing target engagement. The targetable scope of nucleophilic amino acids has moreover expanded beyond cysteine, to encompass serine,^28^ lysine,^29−31^ and arginine,^32^ providing greater control of anchoring sites on the protein and expanded possibilities for more diverse tool compounds that stabilize protein complexes. Covalent drug discovery has already shown significant success in targeting challenging proteins,^15,33−36^ which makes the strategy promising to molecular glue tool compound development.

**Figure 1:**
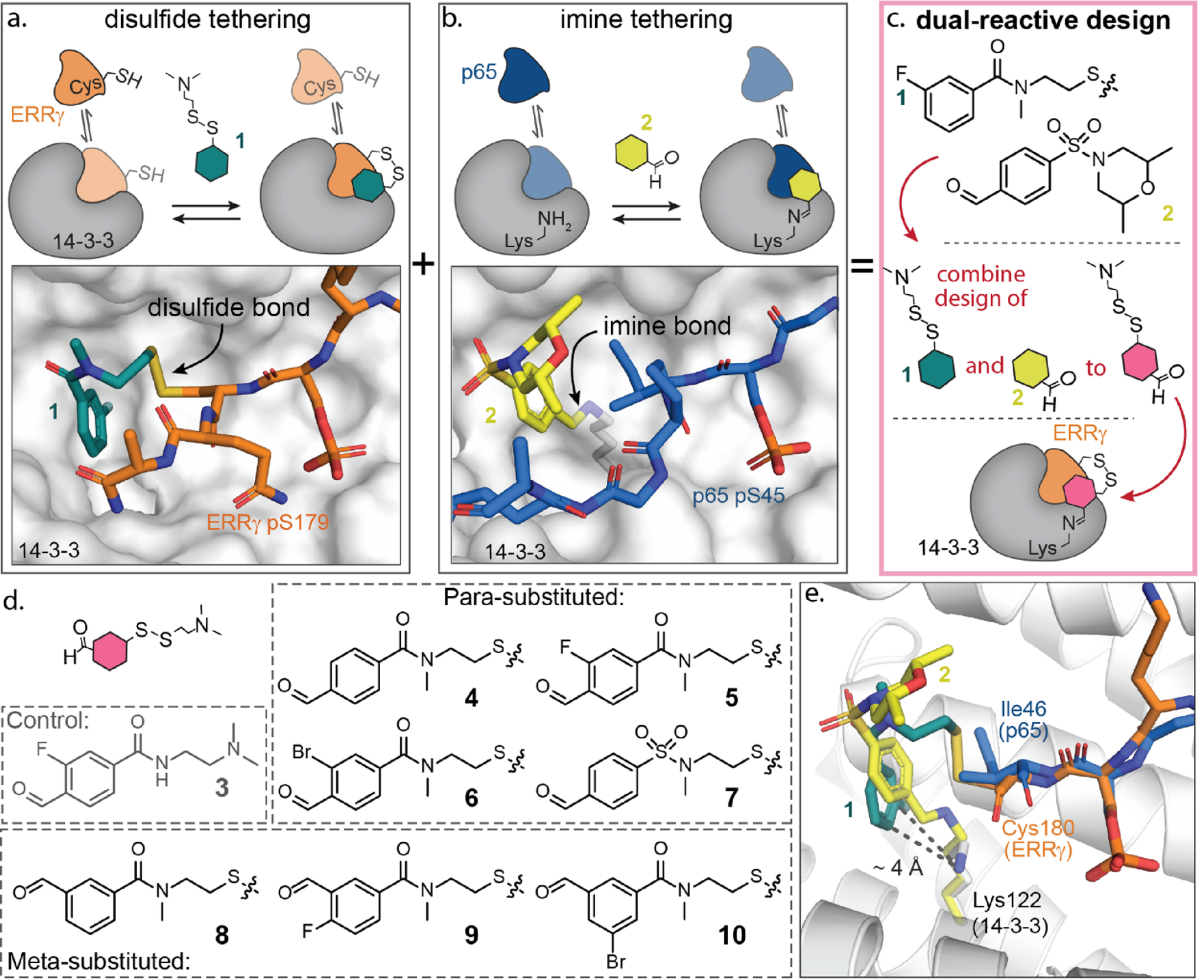
Conceptual design of reversible covalent molecular locks. (a) Schematic depiction of disulfide-based tethering by fragment **1** (cyan hexagon) to cysteine-containing ERRγ (orange) to stabilize the interaction between 14-3-3 (gray) and ERRγ, including an enlarged view of the X-ray crystal structure of **1** and ERRγ bound to 14-3-3σ (gray) (PDB: 6Y3W). (b) Schematic depiction of imine-based tethering of fragment **2** (yellow hexagon) to a lysine of 14-3-3 (gray) to stabilize the interaction between 14-3-3 and p65 (blue), including an enlarged view of the X-ray crystal structure of **2** and p65 bound to 14-3-3σ (PDB: 6YQ2). (c) Conceptual design of reversible dual-covalent molecular locks based on fragments **1** and **2**. (d) Chemical structures of the control compound (**3**) and reversible dual-covalent locks (**4**–**10**). (e) Structural overlay of disulfide-tethered fragment **1** and imine-tethered fragment **2** within the 14-3-3 binding groove.

The emergence of reversible covalent chemistry on proteins, such as via cyano-acrylamide,^12,37,38^ disulfide,^39−41^ α-ketoamide,^42,43^ and aldehyde^29,31^ electrophiles has further diversified the portfolio and properties of chemical probes and tool compounds in chemical biology. The exchangeable nature of reversible covalent tethering is similarly attractive for molecular glue design. We and our colleagues previously developed reversible covalent fragment tethering approaches to identify initial chemical matter to stabilize various protein complexes involving the hub protein 14-3-3.^44^ Disulfide and imine tethering was instrumental in identifying fragments for stabilization of the 14-3-3/ERRγ and 14-3-3/p65 protein/phosphopeptide complexes, respectively (Figure 1a,b).^29,45^

Inspired by chemical crosslinking, here, we envisioned fusing two such tethered fragments into dual-reactive compounds featuring both an aldehyde and a disulfide reactive handle to reversibly crosslink the 14-3-3/ERRγ protein/phosphopeptide complex (Figure 1c). We term this concept “molecular locking”. Locking provides rapid access to tool compounds that strongly glue a PPI into a stable complex. Previously, dual-reactive groups have shown promise in the stimulus-responsive release of peptides as a drug delivery approach and in the development of a highly selective FGFR4 inhibitor.^46,47^ Our approach uniquely provides an entry to stabilize native PPIs using reversible dual-covalent chemistry crosslinking.

Utilizing the interaction between the hub protein 14-3-3^48−50^ and ERRγ^45^ as a case study, we demonstrate that the molecular locks enhance 14-3-3/ERRγ complex formation and stability. Intact mass spectrometry and X-ray crystallography validate the protein–phosphopeptide crosslinking, and time-dependent fluorescence anisotropy (TD-FA), differential scanning fluorimetry (DSF), and peptide washout experiments provided insight into the mechanism of protein–complex stabilization. Specifically, molecular lock binding is driven by the interplay of molecular reactivity and recognition by the composite PPI binding interface. The molecular lock approach is highly selective as illustrated by the stabilization of the 14-3-3/ERRγ complex in the presence of several other competing high-affinity 14-3-3 client peptides and supported by a mathematical model. Further, cell lysate experiments showed that the approach is applicable to the reactive environment of the cell. The dual-reactive molecular locks provide unique and rapid opportunities for selective PPI stabilization.

## Results and Discussion

### Design and Synthesis of a Molecular Lock

Seven dual-reversible covalent molecules were developed to investigate molecular locking of the 14-3-3/ERRγ protein complex (Figure 1d). The design was based on the X-ray crystal structure of fragment **1** in complex with 14-3-3/ERRγ^45^ and fragment **2** in complex with 14-3-3/NF-κB^29,51^ (Figure 1). Previously, we have shown that fragment **1** forms a disulfide bond with Cys180 of ERRγ and **2** reacts with Lys122 of 14-3-3σ via the formation of an imine bond (Figure 1a,b). Analysis of **1** showed that Lys122 of 14-3-3σ sat proximal to the benzamide ring (∼4 Å) (Figure 1e), which has shown to be a key residue for 14-3-3 molecular glue development.^52,53^ To develop the molecular locks, disulfide fragment **1** was extended from either the *m*- or *p*-position of the benzamide ring with a formyl group to react with Lys122 of 14-3-3 (Figure 1c, fragments **3**–**10**). Locks **5–6** and **9–10** were designed based on previous chemistry campaigns, which identified that halogen-substituted phenyl rings probe a subpocket of 14-3-3 shaped by residues Ser45, Phe119, and Met123, resulting in enhanced stabilization.^45,53^ Additionally, the amide bond (τ = 30 and 150°) was replaced with a sulfonyl amide, **7**, (τ = 90 ± 30°) to investigate the significance of torsion angles between the carbonyl/sulfonyl and beta-carbon of the linker.

Compounds **4–6** and **8–10** were accessed via a three-step synthesis. Starting from an amide coupling of the benzoic acid with cystamine HCl, subsequent N-methylation and disulfide exchange with 2,2′-bis(dimethylamino)diethyl disulfide dihydro chloride afforded the compounds in acceptable yield (Scheme S1). Compound **7** was synthesized via an amide coupling from the diacetate sulfonyl chloride precursor followed by deprotection, N-methylation, and disulfide exchange (Scheme S2).

### Molecular Locks Stabilize the ERRγ/14-3-3 Complex

**Figure 2:**
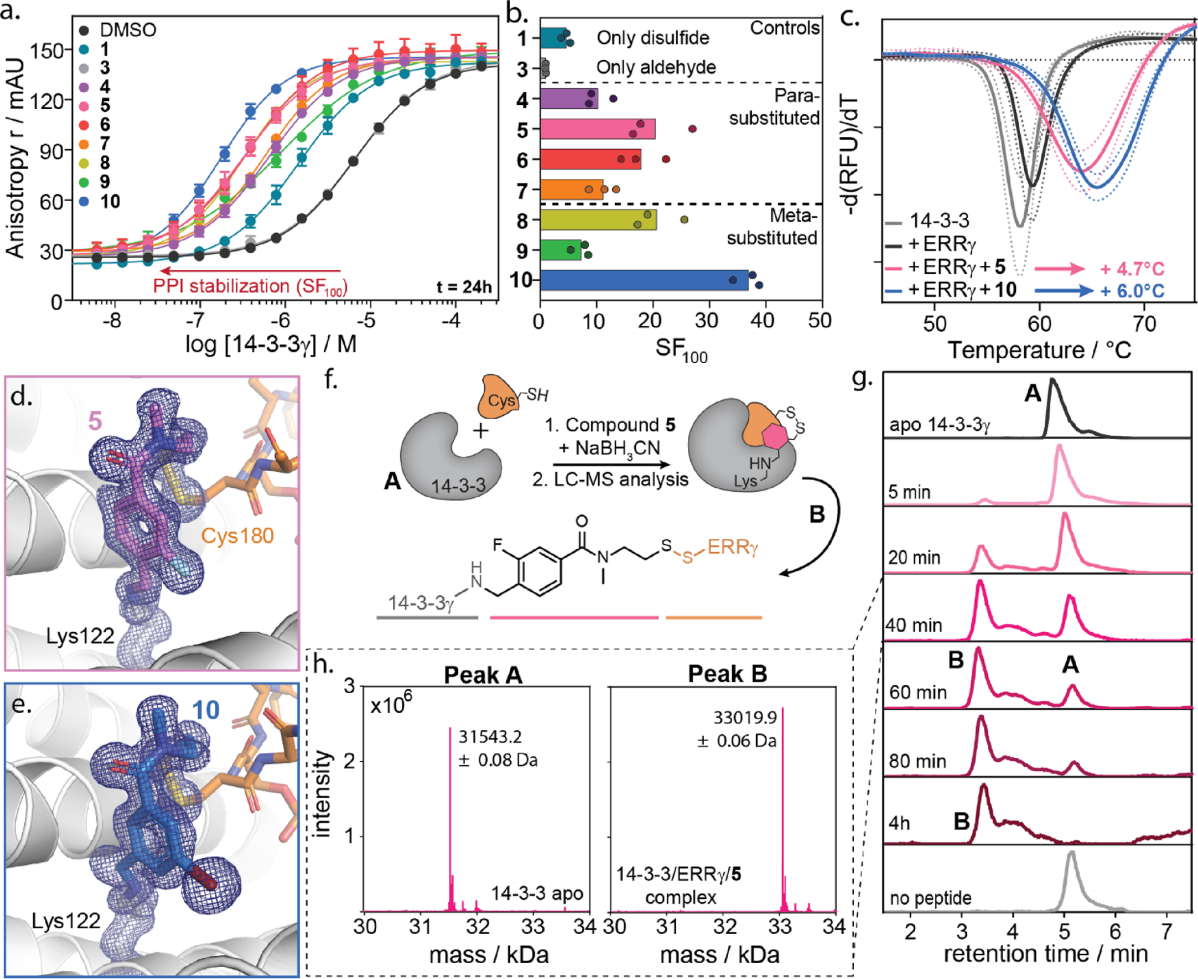
Reversible dual-covalent lock stabilization of the 14-3-3/ERRγ protein complex. (a) Fluorescence anisotropy assay data of a 14-3-3γ titration to 10 nM fluorescein-ERRγ peptide with 100 μM compound (or DMSO as a negative control). (b) Bar plot of the stabilization factor (SF_100_, fold-increase in affinity) induced by 100 μM of each compound acting on the 14-3-3γ/ERRγ complex. (c) Differential melting curve of 14-3-3γ (5 μM, gray), the binary 14-3-3γ/ERRγ (10 eq.) complex (black), and 14-3-3γ/ERRγ with 200 μM lock **5** (pink) or **10** (blue). Increases in melting temperature *T*_m_ as induced by compounds **5** and **10** are provided. (d,e) Crystal structures of **5** (PDB: 8B4Q) and **10** (PDB: 8B5P) covalently bound to Lys122 14-3-3σ C38N (white cartoon) and Cys180 of ERRγ (orange sticks). The 2Fo–Fc electron density map (blue mesh) is contoured at 1σ. (f) Schematic representation of ternary complex formation of 14-3-3γ, ERRγ, and compound **5**, and the analysis through QTOF-MS including the chemical structure of compound **5**, covalently bound to both 14-3-3γ and ERRγ. (g) Normalized LC–MS chromatograms of *apo*-14-3-3γ, 14-3-3γ incubated with ERRγ and compound **5** after different incubation times, and 14-3-3γ incubated with compound **5** only (no peptide) for 6 h. (h) Deconvoluted mass spectra of the two mains peaks of the 14-3-3/ERRγ**/5** ternary complex formation after 40 min of incubation, indicating the presence of *apo*-14-3-3γ in peak A (calculated mass: 31542.8 Da) and the ternary complex of 14-3-3γ/ERRγ/**5** in peak B (calculated mass: 33,019.5 Da).

The stabilization of the ERRγ/14-3-3γ complex by the molecular locks was assessed using a fluorescence anisotropy (FA) assay where 14-3-3γ was titrated into a fluorescently labeled ERRγ phosphopeptide in the absence or presence of a 100 μM molecular lock (Figure 2a,b); this compound concentration was selected based on a FA compound titration assay (Figure S1). Complex stabilization after a 24 h incubation was quantified by dividing the *K*_D_ of the binary 14-3-3/ERRγ complex (DMSO control) by the apparent *K*_D_ (*K*_D_^app^) of the 14-3-3γ/ERRγ complex upon stabilization with 100 μM of each compound, providing the stabilization factor (SF_100_) (Figure 2b and Tables S1 and S2). Notably, we define *K*_D_^app^ as the binding affinity of the ERRγ peptide to 14-3-3γ in the presence of a stabilizer.

Compounds **1** and **4**–**10** all induced a leftward shift of the binding curve compared to the binary 14-3-3γ/ERRγ complex (*K*_D_ = 6.5 ± 0.9 μM), indicating stabilization of the PPI (Figure 2b and Table S1). Compound **1**, containing only the disulfide moiety, elicited a five-fold stabilization of the 14-3-3γ/ERRγ complex (*K*_D_^app^ = 1.4 ± 0.2 μM), and compound **3**, containing only the aldehyde moiety, elicited no protein complex stabilization (SF_100_ = 1). The dual-reactive molecular locks showed a significant increase in the stabilization factor, with the SF_100_ ranging from 7.3 to 37 (14-3-3γ/ERRγ *K*_D_^app^ = 0.92–0.18 μM). Interestingly, the 14-3-3/ERRγ composite pocket favored an *m*-substitution of the reactive handles compared with a *p*-substitution (**8** vs **4**). Substitution of the amide for a sulfonyl amide showed no significant change in the stabilization^.^ (**4** vs **7**). Halogen-substituted benzamide rings improved stabilization (**5**, **6**, and **10**), except for **9**. The optimal combination proved to be a 3,5-substitution of the bromine and aldehyde functionalities (**10**).

Time-dependent fluorescence anisotropy (TD-FA) measurements provided insights in the kinetics of ternary complex (14-3-3/ERRγ/lock) formation (Figures S2 and S3). All fragments reached saturation by ∼12 h. Disulfide **1** showed significantly slower binding kinetics than molecular locks **4**–**10**. Carboxamides **4**–**6** and **8**–**10** reached saturation within 5 h. Molecular lock **5** elicited the fastest reaction kinetics, with saturation reached at 2 h. Interestingly, **5** also elicited the second lowest *K*_D_^app^ value.

### Molecular Locks Enhance the Thermal Stability of the ERRγ/14-3-3 PPI Complex

Differential scanning fluorimetry (DSF) measurements revealed the effect on the thermal stability of the 14-3-3/ERRγ PPI complex by the molecular locks. We screened the *apo*-14-3-3γ, the binary 14-3-3γ/ERRγ (10 eq.) complex, and the ternary complex of 14-3-3γ, the ERRγ phosphopeptide, and compounds **1** and **3–10** (Figure 2c and Figures S4 and S5). The DSF data corroborated the enhanced stabilization observed in the FA data, with molecular locks **5** and **10** eliciting 14-3-3γ melting temperature increases of 4.7 ± 0.2 and 6.0 ± 0.3 °C, respectively (Figure 2c). In comparison, reference compound **1** decreased the melting temperature by 2.6 °C (Figures S4 and S5), and compound **3** induced no significant change. Interestingly, TD-FA and DSF assays showed that while all carboxamide-based molecular locks elicited similar binding kinetics, a clear difference in the final avidity of the stabilized complex was observed. This result suggests that stabilization of the complex is driven by tripartite molecular complementarity between the molecular lock, ERRγ, and 14-3-3, not simply reactivity.

### Structural Elucidation and Validation of Dual Reversible Covalent Crosslinking

High-resolution X-ray structures (<1.4 Å) were obtained for all molecular locks, except compound **9**, in cocrystallization with the ERRγ phosphopeptide and 14-3-3σ (C38N) (Figure 2d,e and Figures S6 and S7). The 14-3-3σ isoform was used for the cocrystallographic studies because of its favorable crystallization properties. 14-3-3σ features a cysteine (Cys38) in the binding groove in proximity to 14-3-3 Lys122. The cysteine was mutated into an asparagine (C38N), as present in the other 14-3-3 isoforms, to avoid potential reactivity in the crystal structures. The inability of compound **9** to cocrystallize was in line with the FA studies where **9** was the least active. For all other molecular locks, a continuous density was observed for the entire compound, including both the covalent bonds with Lys122 of 14-3-3 and Cys180 of ERRγ (Figure S7). In addition to the dynamic covalent bonds, the compounds also make noncovalent interactions with both 14-3-3 and with ERRγ, responsible for the tripartite molecular complementarity between the molecular lock, ERRγ, and 14-3-3.

To further confirm the covalent crosslinking in solution, we performed intact mass spectrometry (MS) on the 14-3-3γ/ERRγ/**5** and the 14-3-3γ/ERRγ/**10** protein complexes. To ensure detection of the acid-labile imine-bonded complexes, the covalent imine bond was reduced *in situ* to the corresponding secondary amine using sodium cyanoborohydride. Samples were incrementally taken over 4 h and analyzed with high-resolution MS to determine crosslinking of the 14-3-3γ/ERRγ complex (Figure 2f–h and Figures S8 and S9). Intact MS analysis confirmed time-dependent crosslinking of the ternary complex (14-3-3γ/ERRγ/lock). Treatment with **5** resulted in near quantitative consumption of *apo*-14-3-3γ into the 14-3-3γ/ERRγ/**5** complex with a molecular weight of 33,019.9 Da after 4 h. Notably, ternary complex formation was not observed in the absence of sodium cyanoborohydride (Figure S10). A similar result was also observed for **10** (calculated mass: 33,080.4 Da) (Figure S11a–c). Notably, although compound **10** eliciting improved stabilizer activity in FA assays, complete consumption of *apo*-14-3-3γ was not observed.

### Tunability of Reversible Covalent Crosslinking

We hypothesize the enhanced avidity of the ternary complex to be a function of the multivalent binding of the dual-reactive groups. Specifically, the dissociation of a molecular lock must pass through two equilibrium reactions leading to high effective molarity of the compound at the protein–protein interface and a high energy barrier for the dissociation of the ternary complex.

**Figure 3:**
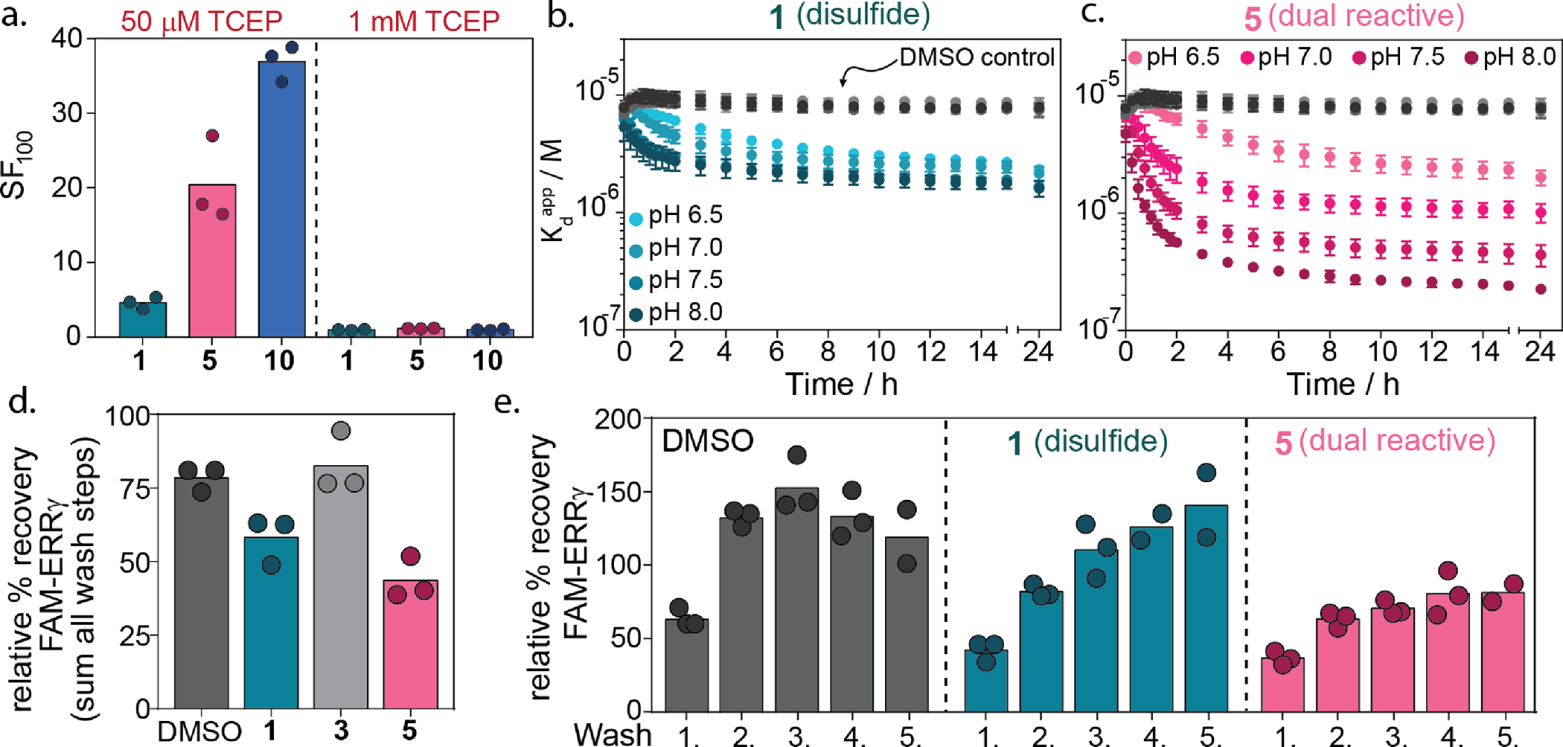
(a) Bar plot representation of the stabilization factor (SF_100_) of 14-3-3/ERRγ complex formation by compounds **1**, **5**, and **10** with either 50 μM TCEP or 1 mM TCEP. (b,c) Time-dependent development of the *K*_D_^app^ of the 14-3-3γ/ERRγ complex with DMSO or 100 μM compound **1** and **5** at different pH values as measured by time-resolved fluorescence anisotropy. (d) Total recovery of the fluorescein-labeled ERRγ 9-mer peptide in a washout experiment of the immobilized 14-3-3γ/ERRγ complex in the presence of DMSO or 100 μM compound **1**, **3**, or **5** after 5 subsequent washing steps (each with a 20 min incubation). Data shown are normalized to the amount of ERRγ recovery in the absence of 14-3-3γ to correct for nonspecific binding of the peptide to the beads. (e) Recovery of the fluorescein-labeled ERRγ 9-mer peptide in a washout experiment of the immobilized 14-3-3γ/ERRγ complex relative to recovery of the fluorescein-labeled ERRγ 9-mer peptide in the absence of 14-3-3γ. Data show ERRγ peptide recovery for five subsequent washing steps individually, in presence of DMSO or 100 μM compound **1** or **5**.

To test the effect of dual reactivity on PPI stabilization, the control compound **1** and molecular locks **5** and **10** were screened in FA and DSF assays in the presence of either 50 μM or 1 mM TCEP. Previous research has shown that low concentrations of reducing agents can enhance disulfide exchange.^10^ In contrast, a high concentration of the reducing agent results in a complete reduction of disulfide bond formation. Analysis of the FA data showed stabilization by compounds **1**, **5**, and **10** at low TCEP concentrations (50 μM) (SF_100_ of 5, 18, and 37, respectively) was completely abolished at a high TCEP concentration of 1 mM (Figure 3a and Figure S12a–c). A similar effect was observed in DSF, where the increase in melting temperature upon addition of the molecular locks was abolished in the presence of high concentrations of TCEP (Figure S13), indicating the need for the disulfide in the dual-covalent lock to stabilize the ERRγ/14-3-3γ complex. The importance of the disulfide was further confirmed in FA assays using an ERRγ C180S mutant (Figure S12d–f).

TD-FA experiments were also performed at different pH values to study ternary complex (14-3-3γ/ERRγ/compound) formation and the importance of the imine bond in the molecular lock (Figure 3b,c and Figures S14–S16). Analysis of the thermodynamic parameters showed that pH did not affect the *K*_D_ of the binary 14-3-3/ERRγ complex (DMSO control) (Figures S14 and S15). Similarly, the avidity of the 14-3-3/ERRγ/**1** complex at equilibrium was not affected by pH (Figure 3b). In contrast, **5** and **10** elicited an inverse relationship between pH and *K*_D_^app^ (Figure 3c and Figure S14), connected to impaired imine bond formation at low pH. Notably, at pH 6.5, molecular locks **5** and **10** even showed similar stabilizing capacity to disulfide **1**, indicating the strong enhancement of the PPI stabilization by the molecular locks upon imine bond formation.

Kinetic analysis showed that binary 14-3-3γ/ERRγ complex formation occurred rapidly, independent of pH, and remained constant over time (Figure 3b, DMSO control). In contrast, the formation of the ternary complexes of 14-3-3γ/ERRγ/compound with **1**, **5**, and **10** was time-dependent and pH-responsive (Figure 3b,c and Figures S15 and S16). Interestingly, whereas thermodynamically, the 14-3-3γ/ERRγ/**1** complex formation was pH-independent, kinetic analysis clearly indicated that the speed of compound **1**-induced PPI stabilization was pH-dependent. At pH 6.5, the kinetics of disulfide bond formation was 50% slower. For instance, at pH 6.5, the maximum effect of **1** was reached after a 4.2 h incubation, compared to 1.7–0.8 h for pH 7.0–8.0 (Figure S16g). Similar trends were observed for molecular locks **5** and **10**, where time-dependent complex stabilization was directly affected by pH, with low pH impeding ternary complex formation (Figure 3c and Figures S15 and S16). Effective disulfide formation thus appeared to aid in the imine bond formation of the dual-covalent locks. Combining the two reactive handles increased the effective molar concentration of the lock within the binding pocket, illustrating the need for dual reactivity and leading to enhanced 14-3-3/ERRγ avidity.

### Molecular Locks Inhibit Complex Dissociation

Next, a washout experiment was performed to understand ternary 14-3-3/ERRγ/molecular lock complex dissociation. Fluorescein-labeled ERRγ was incubated overnight with biotin-functionalized 14-3-3γ in the presence of either DMSO, disulfide **1**, aldehyde **3**, or molecular lock **5** and subsequently immobilized on streptavidin-functionalized magnetic beads (Figure S17a). The beads were then washed five times in a peptide and compound-free buffer. During each wash step, the beads were incubated in the buffer for 20 min to allow dissociation of the ERRγ peptide from the immobilized 14-3-3γ. The fluorescence was then measured for all samples before capture, after capture, and after each washing step (Figure S17b), and the amount of peptide recovery was calculated. Notably, the final data were normalized against recovery of the ERRγ peptide in the absence of biotinylated 14-3-3γ to correct for nonspecific ERRγ binding (Figure S17d). Analysis of the washout experiment showed a significant reduction in the total recovered ERRγ peptide for disulfide fragment **1** and dual-reactive molecular lock **5**, with 58 and 44% peptide recovery, respectively (Figure 3d). The analysis of individual washing steps provided further insight into the ternary complex dissociation kinetics (Figure 3e). Washout experiments of the binary 14-3-3γ/ERRγ complex (DMSO) showed that the ERRγ peptide quickly dissociates from immobilized 14-3-3γ. In contrast, a significant reduction in initial peptide recovery was observed for **1** and **5** (63% for DMSO vs 42% with **1** and 36% with **5**), indicating kinetic stabilization of 14-3-3γ/ERRγ complex. Subsequent washing steps showed significantly more peptide release for **1** compared to **5**. Notably, after five washes, incomplete recovery of the ERRγ peptide was observed for **5**. These results indicate that the presence of a second reactive handle slows down the dissociation of the ternary complex as the molecular lock must pass through two equilibrium reactions.

### The Interplay of Dual Reactivity and Templating Drives PPI Selectivity of the Molecular Locks

**Figure 4:**
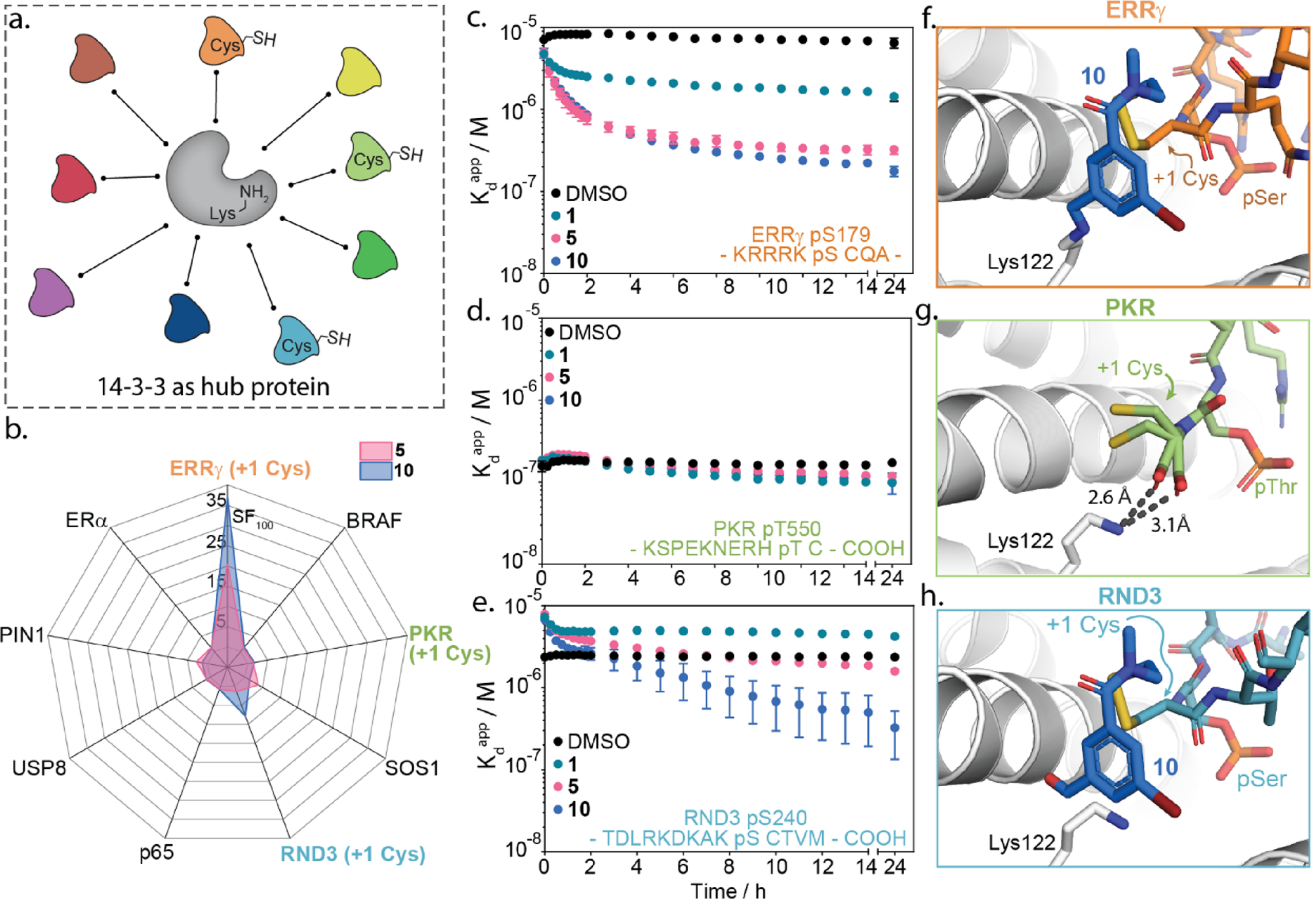
Selectivity of reversible dual-covalent locks. (a) Schematic representation of 14-3-3 as a hub protein (gray blob) where it binds to many different (phosphorylated) binding partners (colored blobs). (b) Radar plot of the stabilization factor (SF_100_) from FA binding studies of compounds **5** and **10** to 14-3-3γ with nine different phosphopeptides. (c–e) Apparent *K*_D_ of the 14-3-3γ/ERRγ, 14-3-3γ/PKR, or 14-3-3γ/RND3 complex with DMSO or 100 μM compound **1**, **5**, and **10** over time as measured by TD-FA. (f–h) Crystal structures of 14-3-3σ (white cartoon) bound to ERRγ pS179 and **10** (orange sticks), PKR pT550 (green sticks), or RND3 pS240 and **10** (blue sticks), which all contain a +1 cysteine relative to the phosphoserine (PDBs: 8B5P, 8B17, and 8B2K).

Drugging the hub protein 14-3-3 raises the challenge of selectivity, given that 14-3-3 binds several hundred phosphoprotein partners (Figure 4a).^54^ We hypothesized that the interplay of chemical reactivity, the topology, and functionality of the composite binding pocket shaped by 14-3-3 and the interaction partner drives selective stabilization of the 14-3-3/ERRγ complex.^53^ To validate selectivity, molecular locks **5** and **10** were tested against a panel of nine 14-3-3 client peptides in an FA assay, and the SF_100_ was determined for each 14-3-3 PPI (Figure 4b and Figures S18–S20). This client panel contained a diverse representation of 14-3-3 binding proteins, differing for example in size and hydrophobicity of the +1 amino acid. Importantly, clients PKR and RND3, each containing a +1 Cys, were included in the panel.

Molecular locks **5** and **10** proved highly selective for the 14-3-3/ERRγ complex in the client screen (Figure 4b). Molecular lock **5** elicited minor off-target effects against SOS1 and PIN1, which contain a +1 Ala and Trp, respectively. Lock **10** also showed weak stabilization of RND3 (SF_100_ ∼ 7.9).

To further assess off-target reactivity to +1 Cys-containing peptides, fragment **1**, locks **5** and **10** were also screened against PKR and RND3 in the TD-FA assay. In contrast to binding to ERRγ, fragments **1**, **5**, and **10** showed no stabilization of the PKR complex (Figure 4d). An interesting binding profile was observed for 14-3-3γ/RND3 modulation by fragments **1**, **5**, and **10**. At short time lengths, all compounds showed immediate destabilization of the complex, as illustrated by the higher *K*_D_^app^ values in the presence of **1**, **5**, or **10** relative to the binary complex (Figure 4e, DMSO). These results indicate rapid disulfide exchange between the fragments and RND3, leading to reduced RND3 binding to 14-3-3. Over time, molecular lock **10** and to a lesser extent **5** showed an enhancement in RND3 binding to 14-3-3γ, whereas disulfide fragment **1** remains an inhibitor. This provides an indication of the order of complex assembly for molecular locks **5** and **10**, suggesting that disulfide formation positions the lock in proximity to Lys122 of 14-3-3 for imine bond formation, causing the observed inhibitor-to-stabilizer behavior.

To mechanistically understand the differences in affinity profiles of **5** and **10**, we investigated the structural changes between ERRγ, PKR, and RND3 binding to 14-3-3 via protein cocrystallography. The PKR phosphopeptide bound 14-3-3 via a sequence with a C-terminal cysteine. The cysteine occupied two conformations, with both conformers of the carboxylic terminus forming an electrostatic interaction with Lys122 of 14-3-3σ (Figure 4g). This positioned both conformations of the cysteine side chain into a small amphipathic subpocket of 14-3-3σ. The positioning of the thiol side chain and the electrostatic interaction made the +1 Cys and Lys122 of 14-3-3 inaccessible for molecular lock crosslinking. Analysis of lock **10** with the 14-3-3/RND3 complex showed clear electron density for the disulfide bond, but only partial imine bond formation was observed with the noncovalent free aldehyde being the major species (Figure S21). We reasoned that **10** adopted an unfavorable conformation for crosslinking, potentially a function of the steric clash between +3 Val of RND3 and **10** that destabilized the interactions. In ERRγ, a less sterically bulky +3 Ala is present (Figure 4f). The lack of covalent crosslinking for lock **10** provides explanation for the differences in stabilization when comparing ERRγ and RND3. Taken together, these results suggested that dual-reactive molecular lock stabilization of 14-3-3 complexes is driven by an interplay of reactivity and molecular recognition of the composite 14-3-3/partner protein pocket.

### Molecular Locks Selectively Crosslink the 14-3-3/ERRγ Complex in a Competitive Environment

**Figure 5:**
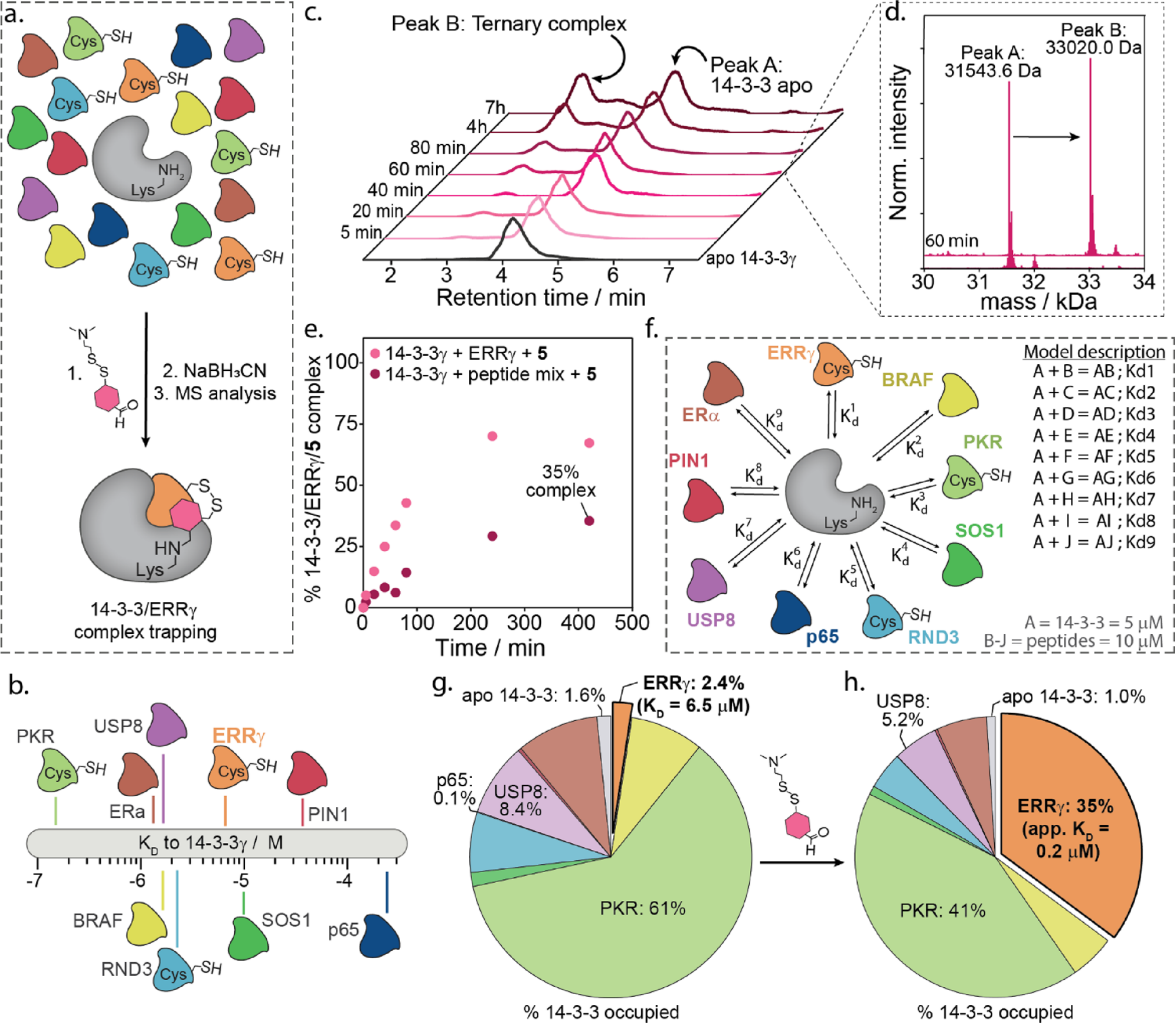
(a) Schematic representation of the QTOF-MS experiment in which 14-3-3γ is mixed with nine different phosphopeptides of which three contain a +1 cysteine residue. A dual-reactive compound is added to form the 14-3-3γ/ERRγ/**5** ternary complex. At the indicated time points, NaBH_3_CN is added to reduce the imine bond to an amine, and the sample is analyzed on high-resolution LC–MS to determine the amount of 14-3-3γ/ERRγ/**5** ternary complex formation. (b) Representation of the affinities of all phosphopeptides to 14-3-3γ. (c) Chromatograms of the time-dependent ternary complex of 14-3-3γ/ERRγ/**5** formation from 14-3-3γ (5 μM), ERRγ (10 μM), and **5** (50 μM) in the presence of eight additional phosphopeptides (10 μM each). (d) Deconvoluted mass spectra associated with peak A and peak B after a 60 min incubation time. (e) Percentage of 14-3-3γ/ERRγ/**5** complex formation over time when having only the ERRγ phosphopeptide with 14-3-3γ and compound **5** or when using a mixture of nine phosphopeptides. (f) Schematic representation of the thermodynamic model of one species A (14-3-3) binding to nine other species B–J (phosphopeptides) with each their own affinity to A. (g) Thermodynamic model results of client binding to 14-3-3 in a complex environment consisting of nine competing peptides under nonstabilized conditions. Here, occupancies of each client binding to 14-3-3 are derived from the model parameters and the *K*_D_ values obtained from FA measurements. (h) Thermodynamic model results of client binding to 14-3-3 in a complex environment consisting of nine competing peptides in the presence of **5**. Here, the apparent *K*_D_ of ERRγ to 14-3-3 was determined based on the 35% conjugation observed in intact MS experiments and the *K*_D_ values of the binary 14-3-3/client protein complex (eight competing peptides) derived from FA measurements.

14-3-3 proteins interact with many phosphorylated proteins, with differing affinities and cellular concentrations.^44,49,50^ On the cellular level, there is a large reservoir of 14-3-3, typically in vast excess to its binding partners. Notwithstanding, we were interested in developing a testable model for the effect of the molecular locking on a PPI network. To investigate the intricacies of selective stabilization of the 14-3-3/ERRγ interaction in a complex matrix, we performed time-dependent intact MS experiments in a competitive environment of excess of 14-3-3 client phosphopeptides. A mixture of 14-3-3 (1 eq.), molecular lock **5** (10 eq.), ΕRRγ (2 eq.), and eight additional client phosphopeptides (8 × 2 eq.) was incubated, and incremental samples were taken, quenched with sodium cyanoborohydride, and measured (Figure 5). Notably, the peptide panel was designed to contain a spectrum of different partner protein affinities, spanning 3.5 orders of magnitude. The peptide panel contained four higher-affinity and three lower-affinity 14-3-3 binding clients, relative to ERRγ, (Figure 5b) and contained +1 Cys-containing peptides RND3 and PKR.

Analysis of the time-dependent intact MS results showed the formation of a new peak at 2.85 min corresponding to the mass of the crosslinked 14-3-3/ERRγ/**5** complex (33,019.5 Da) (Figure 5c,d and Figures S22 and S23), with a maximum crosslinking efficiency of 35% and no evidence of any other crossed linked 14-3-3 PPI. Remarkably, given the stoichiometry of peptide competitors relative to ERRγ (8:1), only an ∼2-fold reduction in crosslinking capacity was observed compared to the 14-3-3/ERRγ in isolation (∼70%) (Figure 5e).

To gain a thermodynamic understanding of 14-3-3/ERRγ complex formation in the competitive environment, a multicomponent equilibrium model was constructed. The model contained the mass balance and equilibrium equations of a system where one species A (14-3-3) can individually bind to nine other species B–J (partner peptides) with connected dissociation constants Kd1–Kd9 (Figure 5f).^55^ The model derived the fraction of protein complexes based on the concentrations of all species used in the intact MS experiment (A = 5 μM; B–J = 10 μM), and peptide affinities to 14-3-3 were based on FA studies (Figure S24).

Analysis of the nonstabilized equilibrium model (Figure 5g and Figure S24) unsurprisingly showed that peptide affinity dictated the percentage of total 14-3-3 occupied. The highest-affinity peptide, PKR (*K*_D_ = 180 nM), occupied 61% of the total 14-3-3 population, while the low-affinity peptide p65 (*K*_D_ = 260 μM) occupied only 0.1% of the total 14-3-3 population. ERRγ occupied only 2.4% of 14-3-3, based on its *K*_D_ of 6.5 μM. Comparison of the fraction of ERRγ bound (2.4%) in the modeled data with the intact MS result (35%) upon addition of molecular lock **5** indicates that **5** significantly increased the total number of 14-3-3/ERRγ complexes in solution, by 15-fold.

To gain further insight into lock-induced ternary complex (14-3-3γ/ERRγ/**5**) formation, we also modeled the 14-3-3γ/ERRγ molecular lock-stabilized system (Figure 5h and Figure S24). Here, the apparent *K*_D_ of ERRγ to 14-3-3γ was calculated, based on the intact MS-observed 35% complex formation and the *K*_D_ values of the binary 14-3-3/peptide interactions. Notably, a key assumption was made that the other eight peptides were unaffected by the addition of 10 eq. of the stabilizer. The thermodynamic model calculated a *K*_D_^app^ of the 14-3-3γ/ERRγ/**5** complex of 0.2 μM in the presence of 50 μM **5**. Further analysis of the modeling data showed that the cost for enhanced 14-3-3 binding to ERRγ was paid by all other complexes roughly equally, with a 33–42% fractional reduction in occupancy of bound 14-3-3 complexes observed for all other client protein interactions. Notably, as a percentage, low-affinity binders contributed marginally more to the increased ERRγ binding, with the low-affinity binder p65 having the largest fractional reduction (42%) in 14-3-3 binding and the high-affinity binder PKR the lowest fractional reduction (33%). However, the strongest binders contributed the most of the 14-3-3 required in terms of total quantity. It should be noted that, in contrast to the situation in a cellular setting, in our model experiment, there is a limiting concentration of hub protein, challenging the redistribution of 14-3-3 with all its interaction partners. The natural large reservoir of 14-3-3 will ensure enough buffering capacity of the protein for functional locking of specific complexes and molecular glue action. Taken together, these results showed the functionality of selective stabilization of a hub protein’s PPI in a competitive environment, with several high-affinity interaction partners in competition for 14-3-3, with our molecular locks.

### Molecular Locks Stabilize the 14-3-3/ERRγ PPI in the Reactive Environment of a Cell Lysate

The cell represents a complex biochemical environment composed of a high reducing potential, a pool of potential nucleophiles, and competing 14-3-3 interaction partners. To investigate molecular lock-induced complex formation in a reactive environment, we first investigated binary 14-3-3/ERRγ formation in the HEK293 cell lysate. Here, the HEK293 cell lysate was titrated to the preformed 14-3-3γ/ERRγ complex in an FA assay and incubated overnight (Figure S25a–c). Analysis of the binary 14-3-3γ/ERRγ PPI showed that the complex was inhibited by the lysate, potentially a result of a competition with high-affinity 14-3-3 binding partners present in the lysate. Next, we investigated molecular lock **5**-induced complex formation (Figures S25 and S26). Gratifyingly, the 14-3-3γ/ERRγ/**5** complex was observed up to 2 mg mL^–1^ cell lysate, albeit at a loss in affinity (half-maximal complex concentration (CC_50_) of 240 μM). Moreover, early observed cell lysate-induced abolishment of the 14-3-3γ/ERRγ complex formation was almost completely recovered upon addition of **5**, to similar complex concentrations to that in the absence of the lysate. This is remarkable given the limitations of disulfide reactive groups as cellularly compatible electrophiles. The result of tool compound **5** shows the potential of this concept for the development of dual-covalent chemical probes with more cellularly compatible electrophiles, such as cyanoacrylamides, to investigate PPI stabilization in cells.

## Conclusions

The design of molecular glues and identification of chemical starting points for PPI stabilization remain a significant challenge for drug discovery. Novel approaches to develop tool compounds and chemical probes are needed, both to investigate the regulatory roles of PPIs and as chemical leads for aiding drug development. Here, we present an integrative approach to develop “molecular glue-like” tool compounds from tethered fragments. We demonstrate that with a relatively small library of compounds, rapidly, a strong stabilization of the 14-3-3/ERRγ PPI can be achieved using dual reversible covalent chemistry. Enhanced complex stability is a function of the multivalent binding of two reversible covalent handles and molecular recognition of the PPI interface, which enhances the effective molar concentration of the ligand within the composite binding pocket. Further, the molecular locks show remarkably selective stabilization of the 14-3-3/ERRγ complex in a competitive environment of other multiple, stronger binding, 14-3-3 interaction partners. The data revealed this selectivity to be a function of the interplay between reactivity and molecular recognition of the molecular locks and PPI. A concomitant mathematical model helps to both understand the complex interaction equilibria in competitive mixtures and to determine resulting affinity constants upon molecular locking of the PPI at hand. The molecular lock approach thus provides a rapid means of coarse-grain assessment of a PPI target for molecular glue development.

The concept of molecular locks, as tool compounds for addressing PPIs, can be easily translated to other reactive handles, such as cyanoacrylamides or α-ketoamides. The recent expansion of targetable nucleophilic amino acids makes this concept modular and expandable to include a wide scope of potential PPI targets. The broad scope of reactive handles allows the chemical properties of the dual-reactive molecular locks to be tailored to the local environment of the binding pocket or the PPI interface, potentially complemented by strategically introduced point mutations for molecular targeting of the locks. The molecular lock concept should not be limited to molecular glues and could easily be implemented to other fields of drug discovery. For instance, this concept should be latterly transferable to covalent PROTACs with the introduction of a second reactive handle providing enhanced residence times of the E3 ligase protein complex through enhanced effective molarity.

## Supporting Information Available

Detailed experimental procedures, Figures S1–S26, and Tables S1–S3 (PDF)
