# Supplementary material for: Reversible Dual-Covalent Molecular Locking of the 14-3-3/ERRγ Protein–Protein Interaction as a Molecular Glue Drug Discovery Approach
Source: J Am Chem Soc. 2023 Mar 16;145(12):6741–52. doi: 10.1021/jacs.2c12781 (PMC10064330; doi:10.1021/jacs.2c12781)
Supplement: Supplementary file 1 — ja2c12781_si_001.pdf [file ja2c12781_si_001.pdf]

Supporting Information for

Reversible dual covalent molecular locking of the 14-3-3/ ERRγ protein-protein interaction as a molecular glue drug discovery approach

Bente A. Somsen[1], Rick J.C. Schellekens[1], Carlo J.A. Verhoef[1], Michelle R. Arkin[2], Christian Ottmann[1], Peter J. Cossar[1*] and Luc Brunsveld[1*]

1 Laboratory of Chemical Biology, Department of Biomedical Engineering and Institute for Complex Molecular Systems, Eindhoven University of Technology, PO Box 513, 5600 MB Eindhoven, The Netherlands
2 Department of Pharmaceutical Chemistry and Small Molecule Discovery Centre (SMDC), University of California, San Francisco 94143, United States

* Corresponding authors
Peter J. Cossar: p.cossar@tue.nl
Luc Brunsveld: l.brunsveld@tue.nl

Content:

Supplementary Figures and Tables……………………………………………………………………..2-33
Experimental Section Biophysical Assays………………………………………………………… 34-37
Experimental Section Chemistry…………………………………………………………………….…38-49
SI References……………………………………………………………………………………………………50

Supplementary Figures and Tables

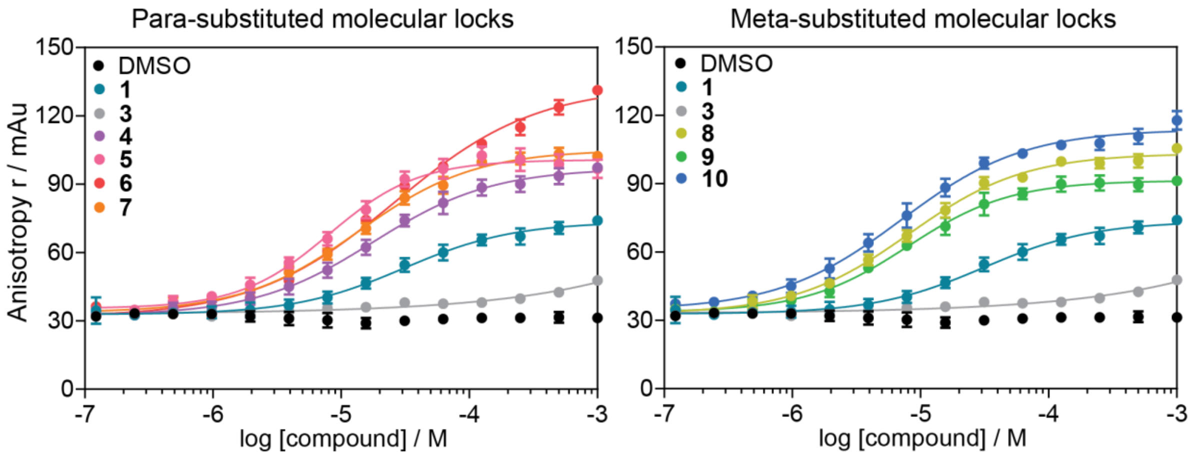

Figure S1. FA compound titrations. Fluorescence anisotropy studies of compound titrations (or DMSO as negative control) to a preformed complex of 14-3-3γ (1 μM) and fluorescein labelled ERRγ peptide (100 nM). Data is separated based on the substitution pattern of the aldehyde and disulfide moiety on the molecular locks.

Table S1. **K_D analysis at t = 24h.** Overview of obtained apparent K_D values of fluorescence anisotropy assay in which 14-3-3γ titration to 10 nM fluorescein-labelled ERRγ peptide in presence of 100 μM compound **1**-**10** (or DMSO as negative control) after 24h incubation (see also Figure 2a). Table represents the obtained apparent K_D for each compound in three independent experiments and the calculated average and standard deviation of these experiments.

K_D	Replicate 1	Replicate 2	Replicate 3	Average	St. deviation
**DMSO**	7.6E-06	6.0E-06	5.9E-06	6.5E-06	9.2E-07
**1**	1.4E-06	1.6E-06	1.3E-06	1.4E-06	1.8E-07
**3**	7.4E-06	6.1E-06	6.6E-06	6.7E-06	6.8E-07
**4**	5.8E-07	7.0E-07	6.5E-07	6.5E-07	5.8E-08
**5**	2.8E-07	3.4E-07	3.6E-07	3.3E-07	4.1E-08
**6**	3.4E-07	4.2E-07	3.5E-07	3.7E-07	4.5E-08
**7**	5.6E-07	5.3E-07	6.8E-07	5.9E-07	8.1E-08
**8**	3.0E-07	3.5E-07	3.1E-07	3.2E-07	2.8E-08
**9**	8.9E-07	1.1E-06	7.5E-07	9.2E-07	1.9E-07
**10**	2.0E-07	1.8E-07	1.5E-07	1.8E-07	2.4E-08

Table S2. **Stabilization factor analysis at t = 24h.** Overview of calculated stabilization factor of each compound at 100 μM (SF_{100}). The SF100 is determined by dividing the apparent K_D of the DMSO control by the apparent K_D of the ternary complex of each compound with 14-3-3γ/ERRγ (see Table S1). Table represents the calculated SF_{100} for each compound in three independent experiments and the calculated average and standard deviation of these experiments.

SF100	Replicate 1	Replicate 2	Replicate 3	Average	St. deviation
**DMSO**					
**1**	5.3	3.7	4.6	4.6	0.8
**3**	1.0	1.0	0.9	1.0	0.1
**4**	13.0	8.6	9.0	10.2	2.4
**5**	27.0	17.8	16.5	20.4	5.7
**6**	22.3	14.3	16.9	17.8	4.0
**7**	13.4	11.4	8.6	11.2	2.4
**8**	25.5	17.3	19.0	20.6	4.4
**9**	8.5	5.4	7.9	7.3	1.6
**10**	37.6	34.1	38.8	36.9	2.4

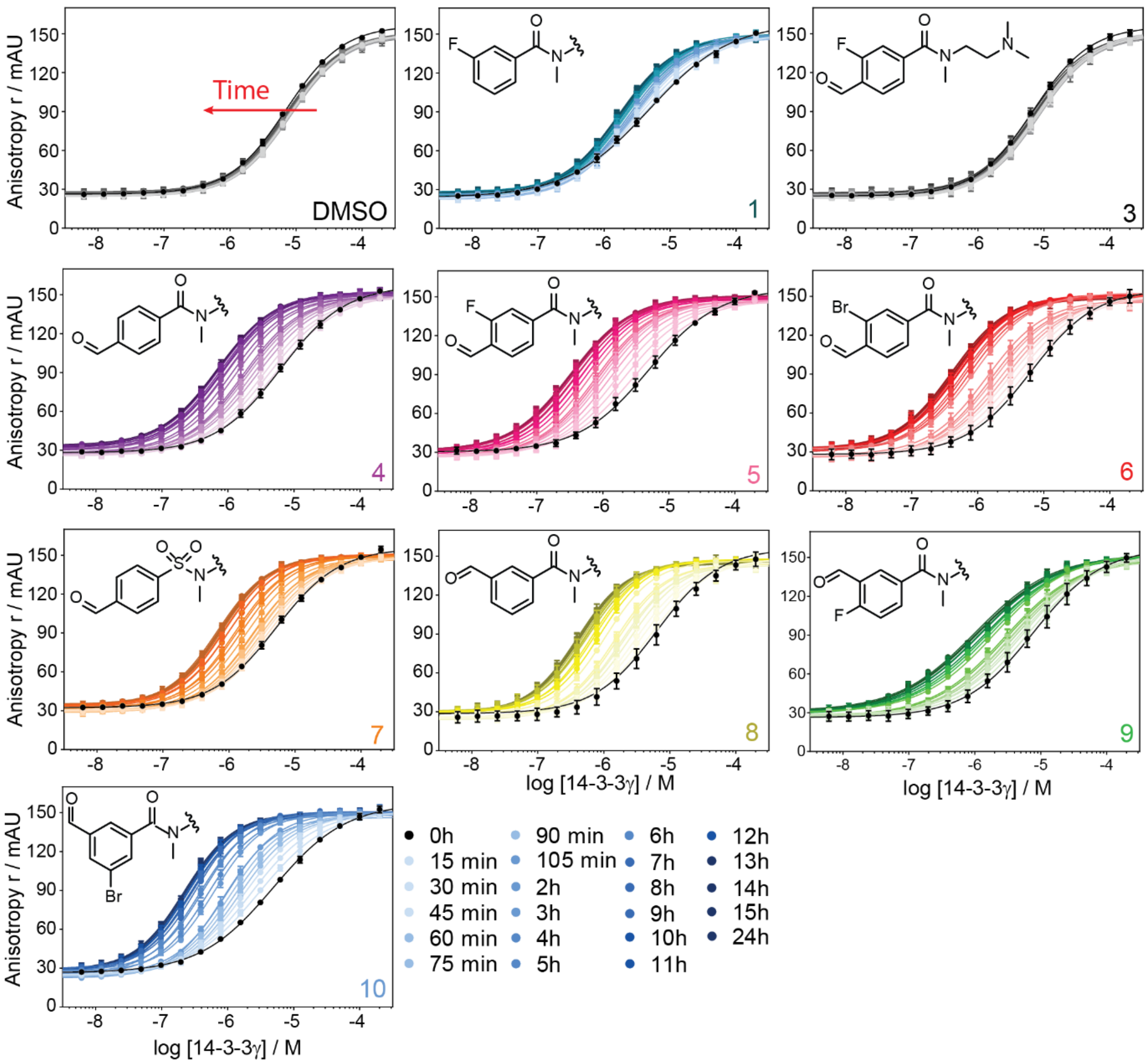

Figure S2. Time resolved fluorescence anisotropy screen. Time dependent results of 14-3-3γ titration to 10 nM fluorescein-labelled ERRγ peptide in presence of 100 μM compound **1**-**10** (or DMSO as negative control). Fluorescence anisotropy was measured every 15 minutes for the first 2 hours after which it was measured every hour for 15 hours. The plate was measured one more time after 24 hours incubation.

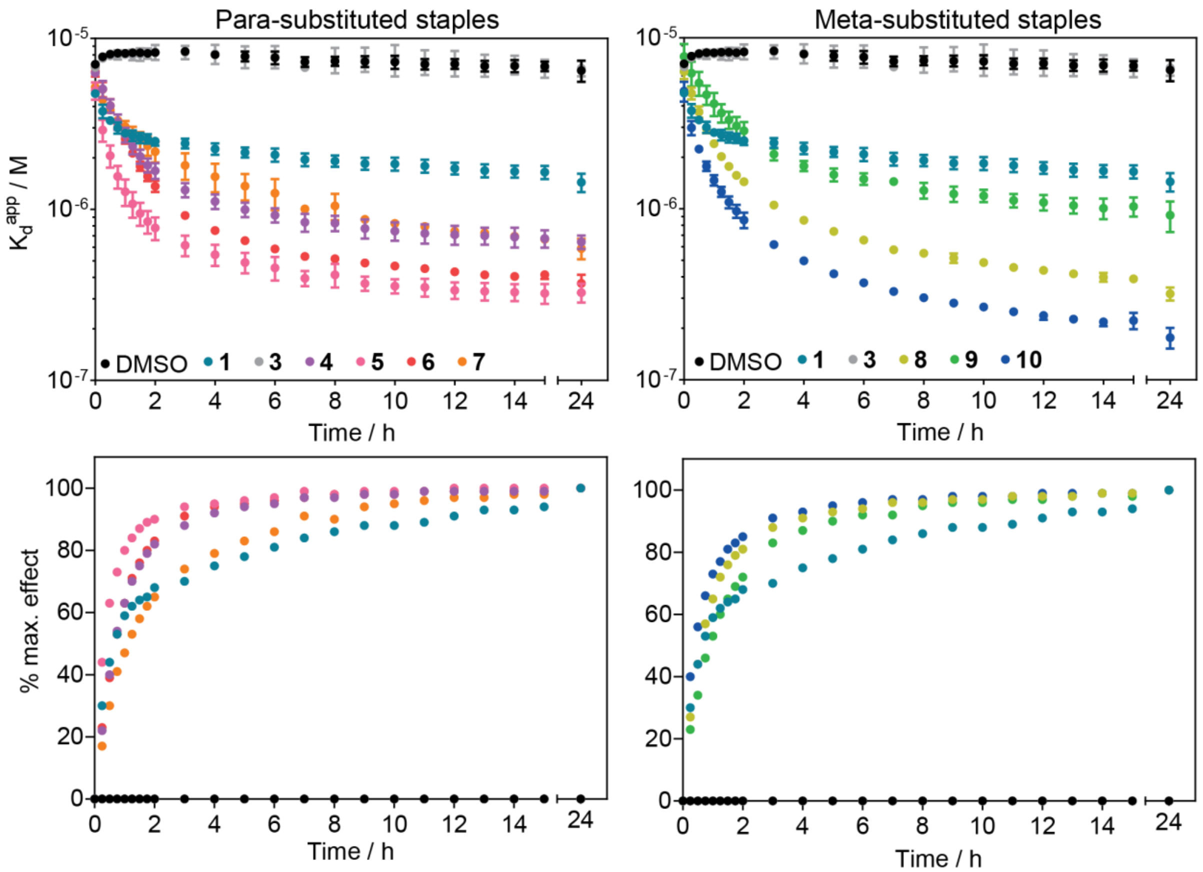

Figure S3. Time-dependent FA-based apparent Kd analysis. Determined apparent K_D from fluorescence anisotropy binding curves (see figure S1) and plotted over time (top row) for all compounds in this study to show a time dependent decrease in K_D (increase in affinity) between 14-3-3γ and ERRγ as induced by the compounds. Bottom graphs show the normalized version of the top graphs where the K_D at t=0h was set to no effect (0%) and the apparent K_D at t=24h as maximal effect (100%).

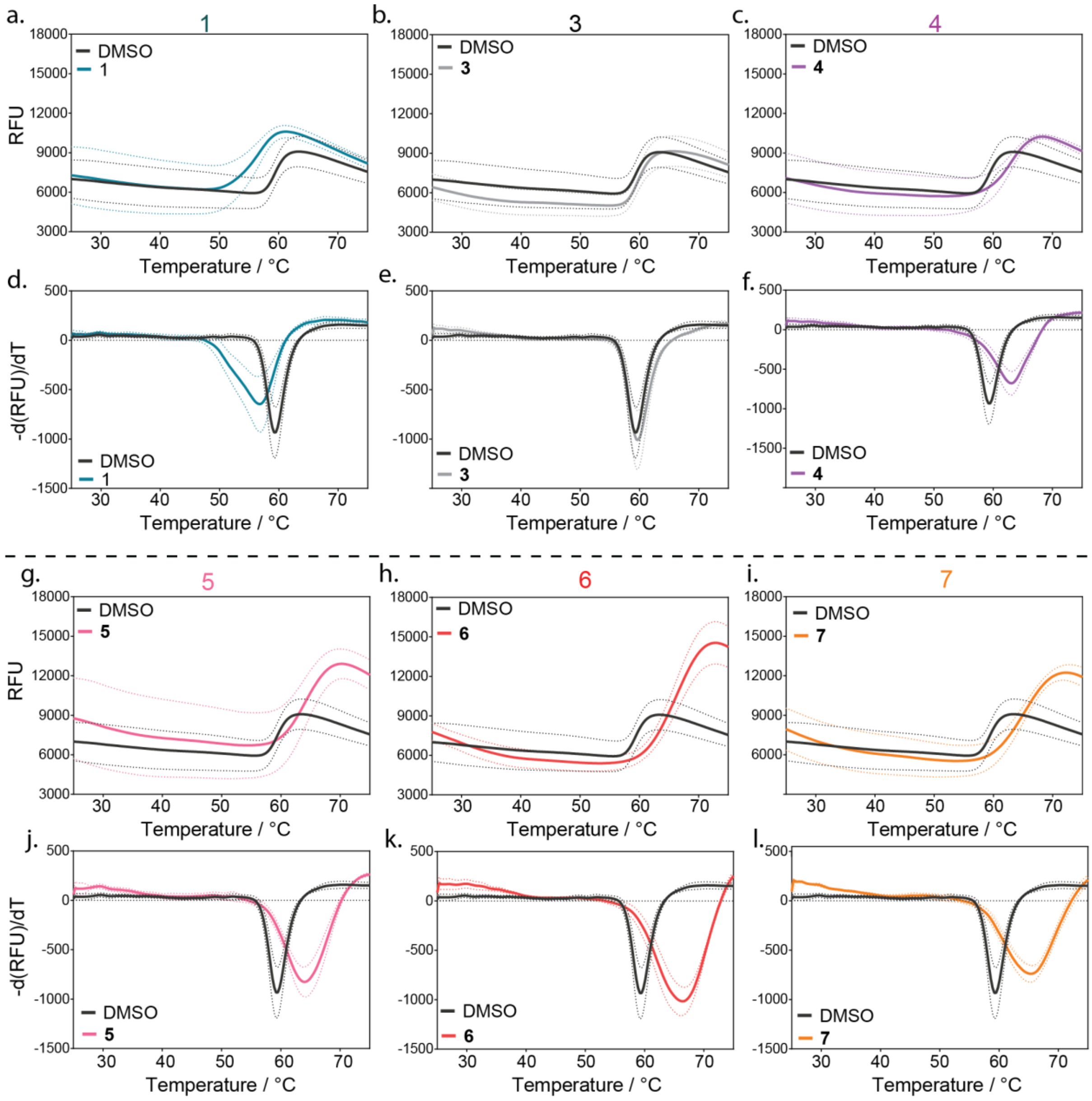

Figure S4. **Differential Scanning Fluorimetry.** Raw melting curves (a-c, g-i) and derivatives of these melting curves (d-f, j-l) of 14-3-3γ (5 μM) with ERRγ (25 μM) incubated with either DMSO (black) or 200 μM compound **1**-**10**.

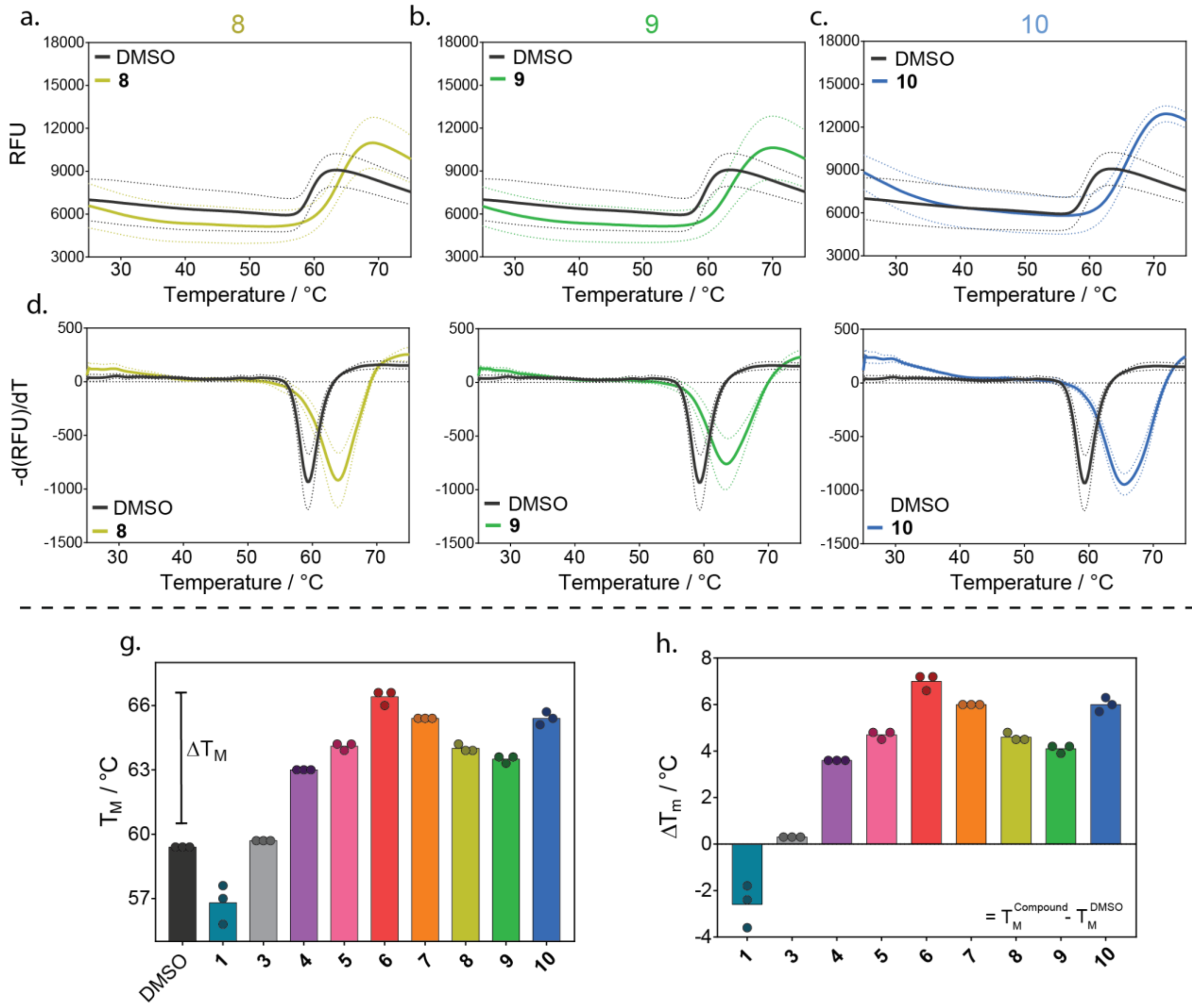

Figure S5. **Differential Scanning Fluorimetry.** Raw melting curves (a-c) and derivatives of these melting curves (d-f) of 14-3-3γ (5 μM) with ERRγ (25 μM) incubated with either DMSO (black) or 200 μM compound **1**-**10**. (g) Identified melting temperatures (T_m) of 14-3-3γ in presence of each of these compounds as determined from the melting curves (h).Difference in melting temperature (ΔT_m) of 14-3-3γ that is induced by each for each om the compounds.

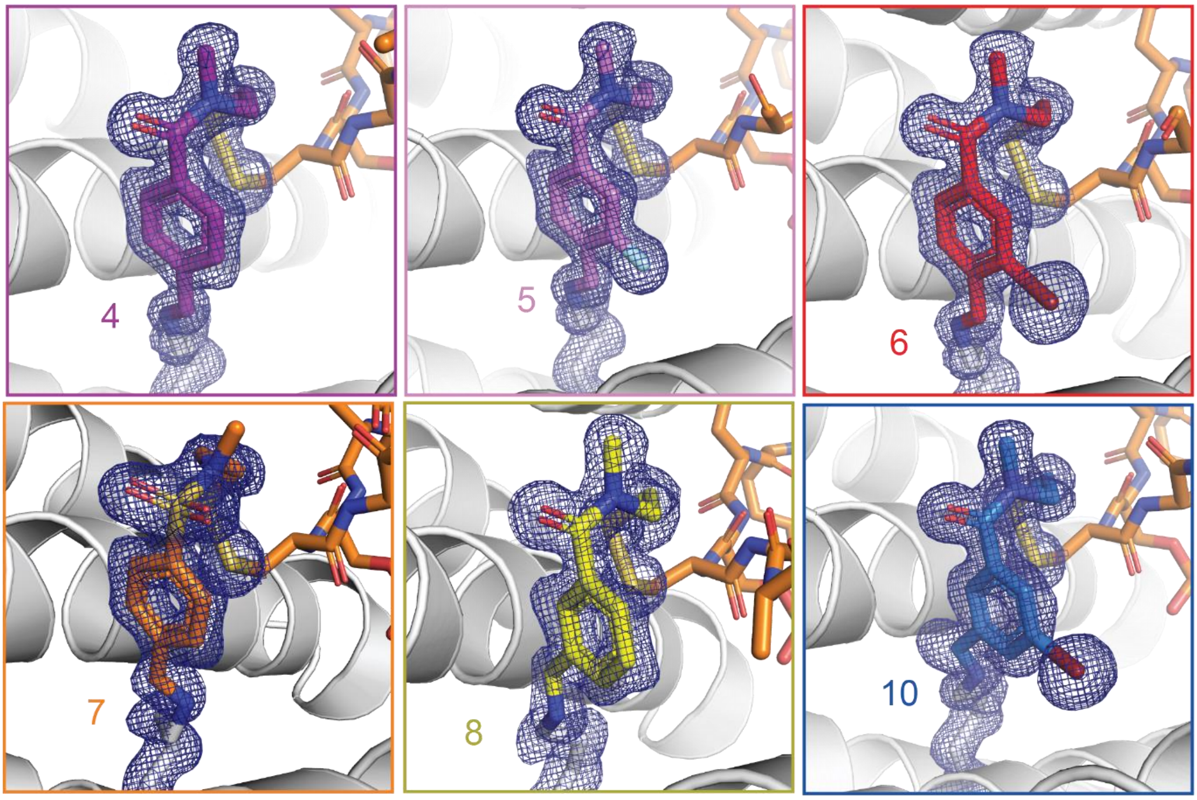

Figure S6. Crystal structures. Crystal structures of molecular locks **4**-**8** and **10**, covalently bound to Lys122 of bound to 14-3-3σ (white cartoon) and Cys180 of the ERRγ peptide (orange sticks). The 2Fo – Fc electron density map (blue mesh) is contoured at 1σ.

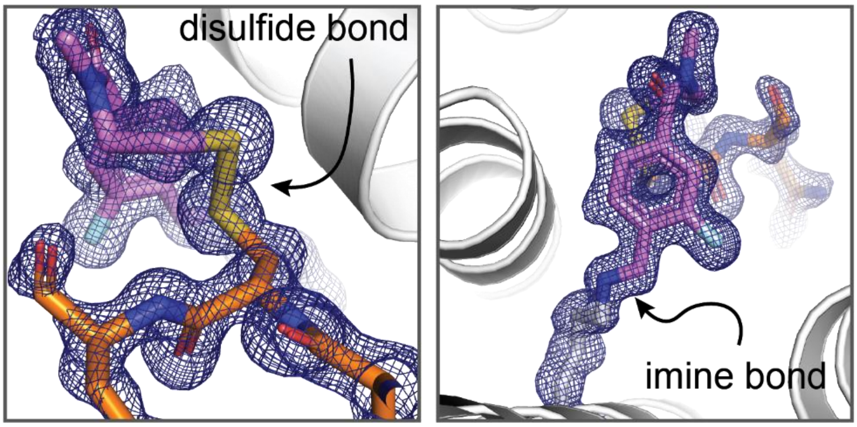

Figure S7. Covalent bond formation with in crystal structures. Crystal structure of molecular staple **5** (pink sticks) with emphasis on the disulfide bond with Cys180 of ERRγ (orange sticks) and imine bond with Lys122 of 14-3-3σ (white cartoon). The 2Fo – Fc electron density map (blue mesh) is contoured at 1σ.

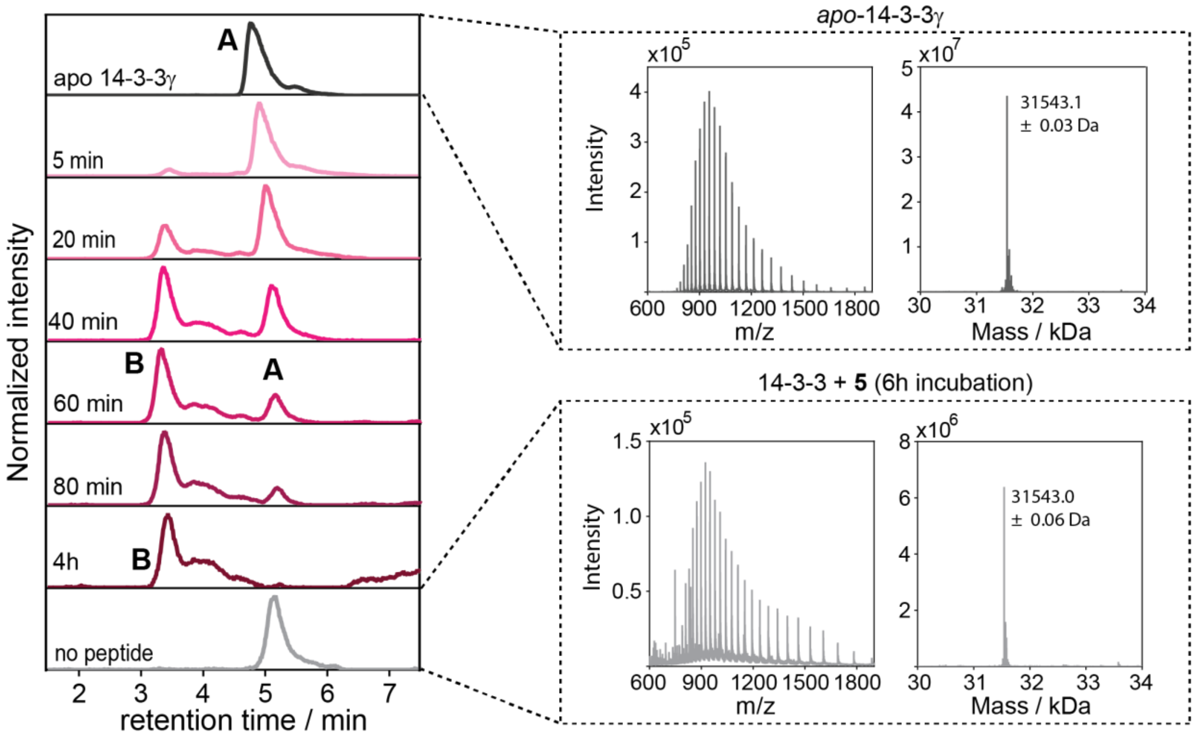

Figure S8a. QTOF-MS analysis of 14-3-3/ERRg/5 ternary complex formation. LC-MS analysis of time-dependent crosslinking experiment of 14-3-3γ (5 μM) and ERRγ (25 μM) phosphopeptide by compound **5** (50 μM). Controls of just apo 14-3-3γ protein and protein incubated with compound **5** without ERRγ peptide are added. (Left) Chromatograms obtained for each sample after x amount of incubation. (Right) m/z spectra and associated mass spectrum of control samples apo 14-3-3γ and 14-3-3γ incubated with **5** (6h incubation). Calculated mass 14-3-3γ apo: 31542.8 Da.

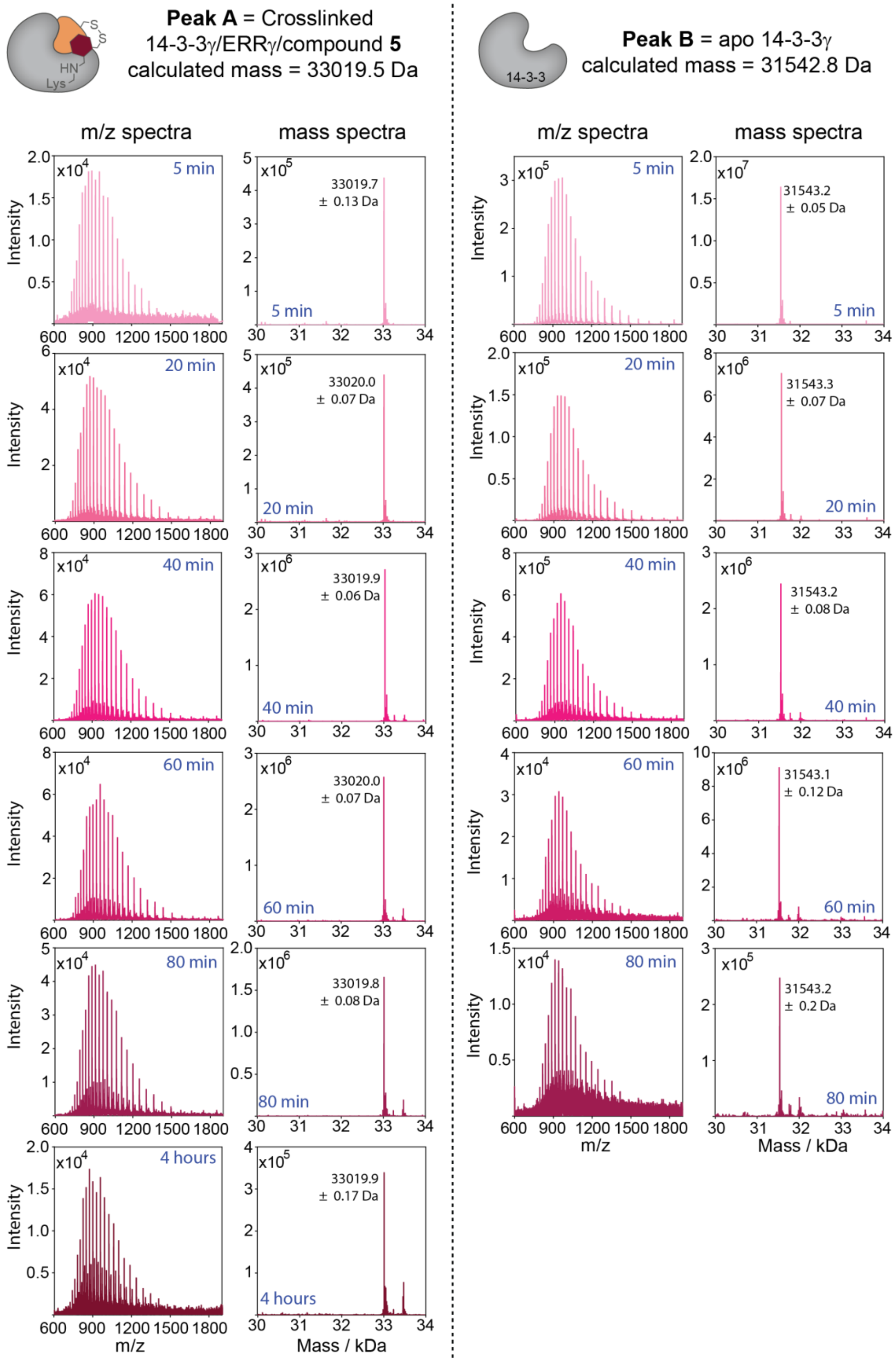

Figure S8b. QTOF-MS analysis of 14-3-3/ERRγ/5 ternary complex formation. m/z spectra and associated mass spectrum of both peak A (retention time: 3.00-3.65 min) and peak B (retention time: 4.75-5.50 min) in chromatograms of Figure S8a with an incubation time between 5 minutes and 4 hours. Calculated mass 14-3-3γ apo: 31542.8 Da; Calculated mass covalently crosslinked 14-3-3γ/ERRγ/**5** complex: 33019.5 Da.

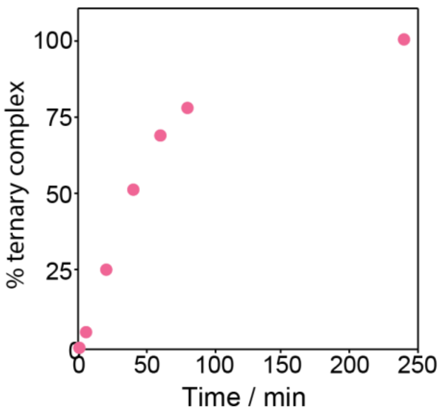

	Peak intensity apo 14-3-3γ	Peak intensity 14-3-3γ/ERRγ/5 complex	% ternary complex
5 min	8095641	432922	5%
20 min	3968503	1349670	25%
40 min	1544695	1628480	51%
60 min	728577	1611928	69%
80 min	330141	1157909	78%
4h	0	399781	100%

Figure S9. Ternary complex formation of 14-3-3/ERRγ/5. Percentage of ternary complex formation over time as identified by QTOF-MS. Based on the determined peak intensity of apo 14-3-3γ (peak A) and 14-3-3γ in complex with ERRg and staple **5** (peak B), the percentage of ternary complex is determined for each time point.

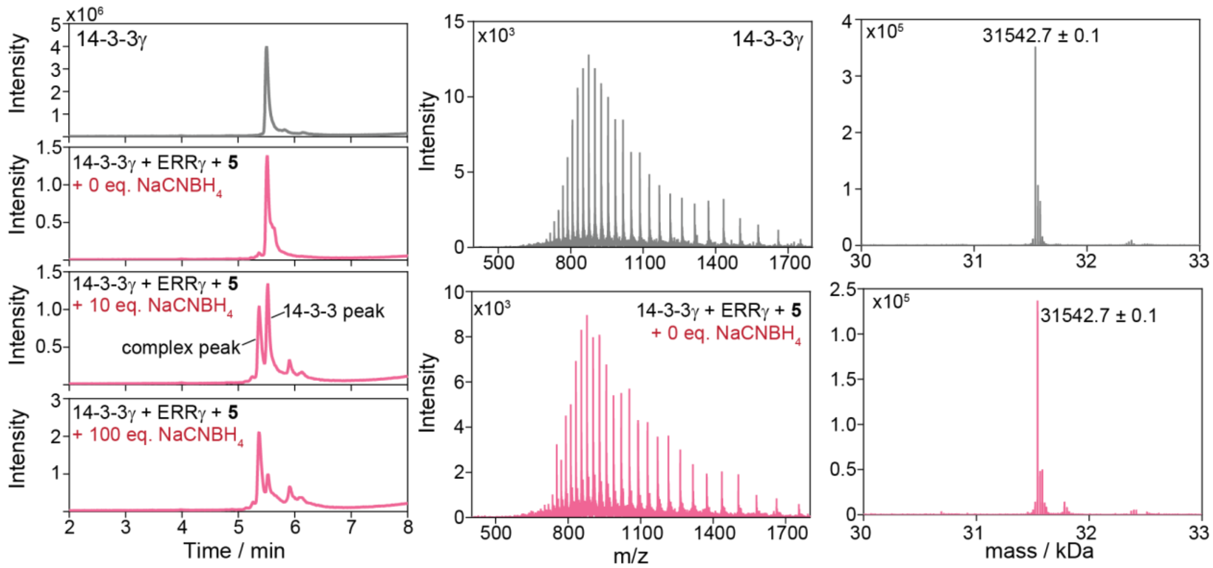

Figure S10. QTOF-MS analysis – effect if NaCNBH4. LC-MS analysis of crosslinking experiment of 14-3-3γ (5 μM) and ERRγ (25 μM) phosphopeptide by compound **5** (50 μM). Samples were incubated for 1h and subsequently reduced with 0, 10 or 100 equivalents sodium cyanoborohydride (relative to compound **5**). Whereas ternary complex formation was observed with 100 eq. $NaBH_3CN$, this was reduced with addition of 10 eq. and completely abolished with 0 eq. $NaBH_3CN$. Mass analysis of the last chromatogram provided a mass similar to that of 14-3-3g alone (calculated mass 14-3-3γ apo: 31542.8 Da).

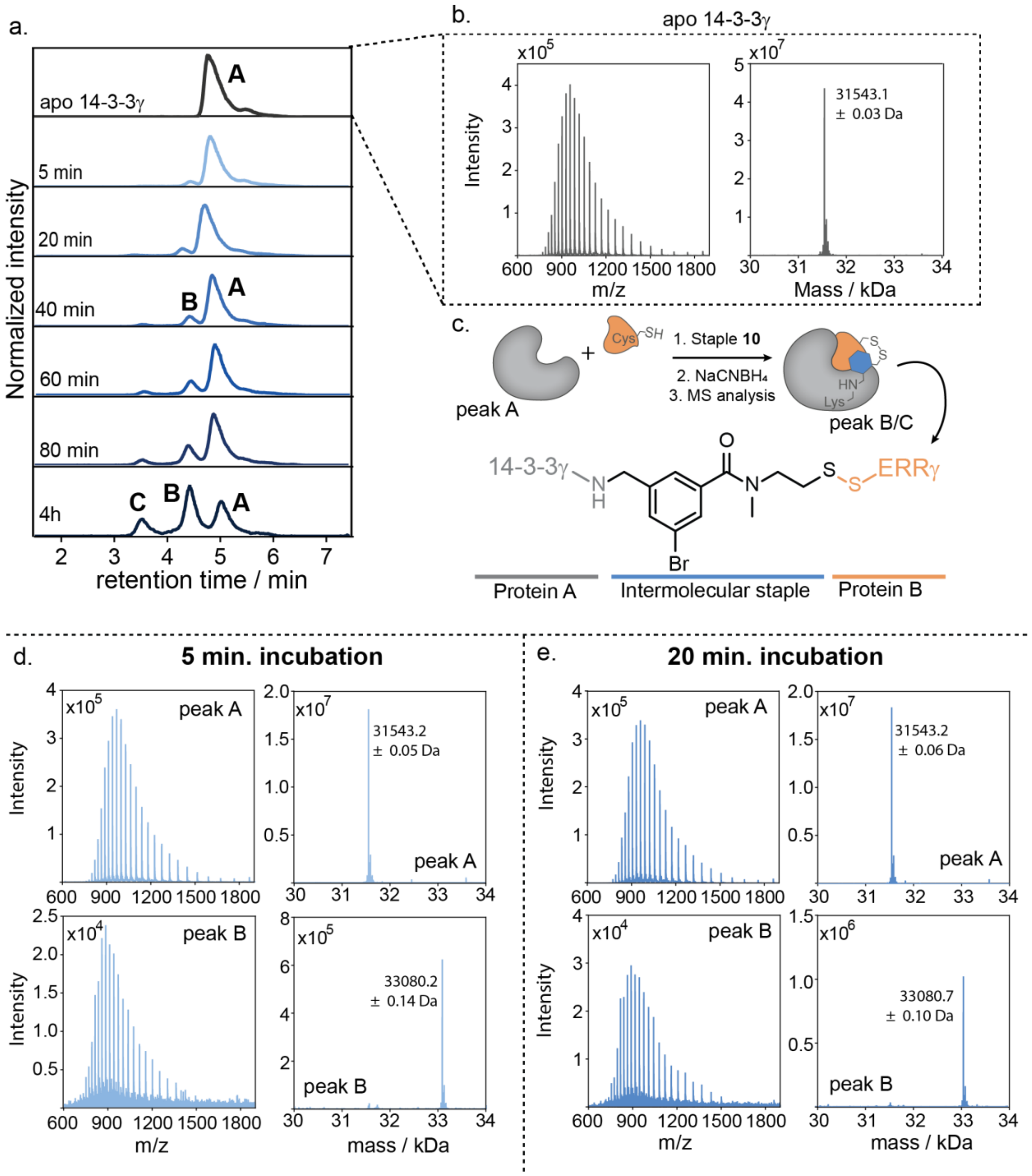

Figure S11a. QTOF-MS analysis of 14-3-3/ERRγ/10 ternary complex formation. LC-MS analysis of time-dependent crosslinking experiment of 14-3-3γ (5 μM) and ERRγ (25 μM) phosphopeptide by compound **10** (50 μM). (a) Chromatograms obtained from each sample after x amount of incubation. (b) m/z spectra and associated mass spectrum of control samples apo 14-3-3γ. (c) Schematic representation of experiment in which 14-3-3γ and ERRγ phosphopeptide are incubated with compound 10 for x amount of time. *In situ* reduction of the imine bond is done using 1000x $NaBH_3CN$. The samples are subsequently measured using LC-MS. (d) m/z spectra and mass spectra of peak A (retention time: 4.75-5.50 min) and peak B (retention time: 4.25-4.55 min) after 5 minutes of incubation. (e) m/z spectra and mass spectra of peak A (retention time: 4.75-5.50 min) and peak B (retention time: 4.25-4.55 min) after 20 minutes of incubation. Calculated mass 14-3-3γ apo: 31542.8 Da; Calculated mass covalently crosslinked 14-3-3γ/ERRγ/**10** complex: 33080.4 Da.

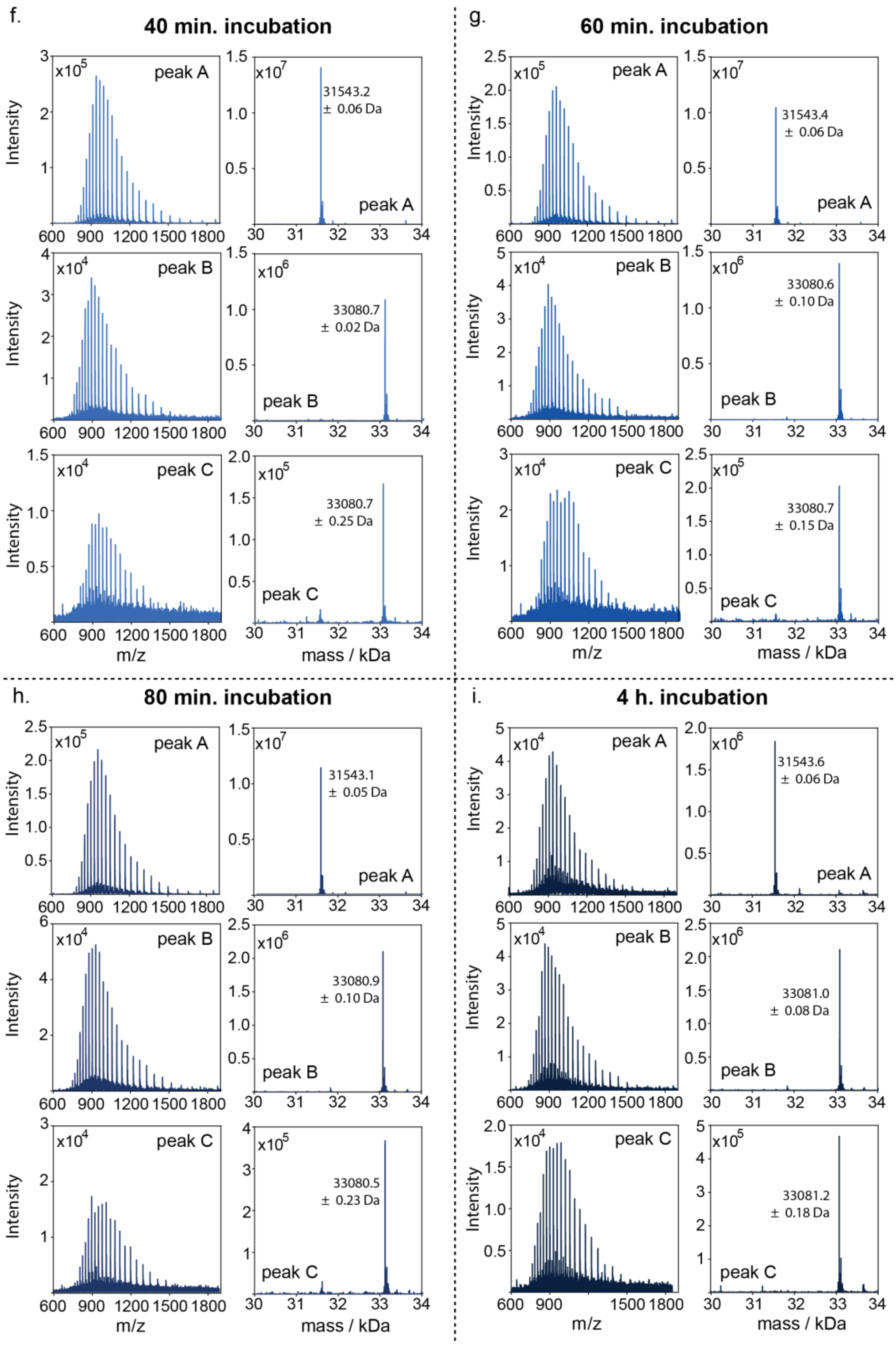

Figure S11b. QTOF-MS analysis of 14-3-3/ERRγ/10 ternary complex formation. LC-MS analysis of time-dependent crosslinking experiment of 14-3-3γ (5 μM) and ERRγ (10 μM) phosphopeptide by compound **10** (50 μM). (f-i) m/z spectra and mass spectra of peak A (retention time: 4.75-5.50 min), peak B (retention time: 4.25-4.55 min) and peak C (retention time: 3.25-4.00 min) in chromatograms of figure S11a after 40 minutes (f), 60 minutes (g), 80 minutes (h) and 4h (i) of incubation. Calculated mass 14-3-3γ apo: 31542.8 Da; Calculated mass covalently crosslinked 14-3-3γ/ERRγ/**10** complex: 33080.4 Da.

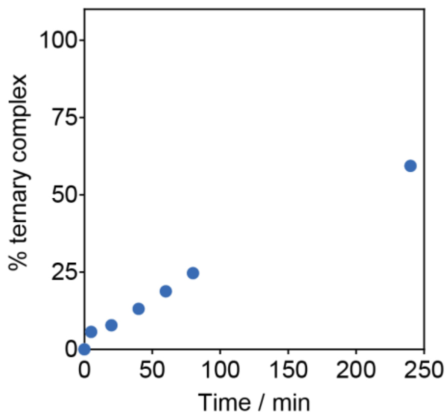

	Peak intensity apo 14-3-3γ (A)	Peak intensity 14-3-3γ/ERRγ/10 complex (B+C)	% ternary complex
5 min	9106371	546880	6%
20 min	8922252	754108	8%
40 min	6760238	1020873	23%
60 min	5344769	1234877	19%
80 min	5504175	1802692	25%
4h	1057025	1543179	59%

Figure S11C. Ternary complex formation of 14-3-3/ERRγ/10. Percentage of ternary complex formation over time as identified by QTOF-MS. Based on the determined peak intensity of apo 14-3-3γ (peak A) and 14-3-3γ in complex with ERRγ and staple **10** (peak B+C), the percentage of ternary complex is determined for each time point.

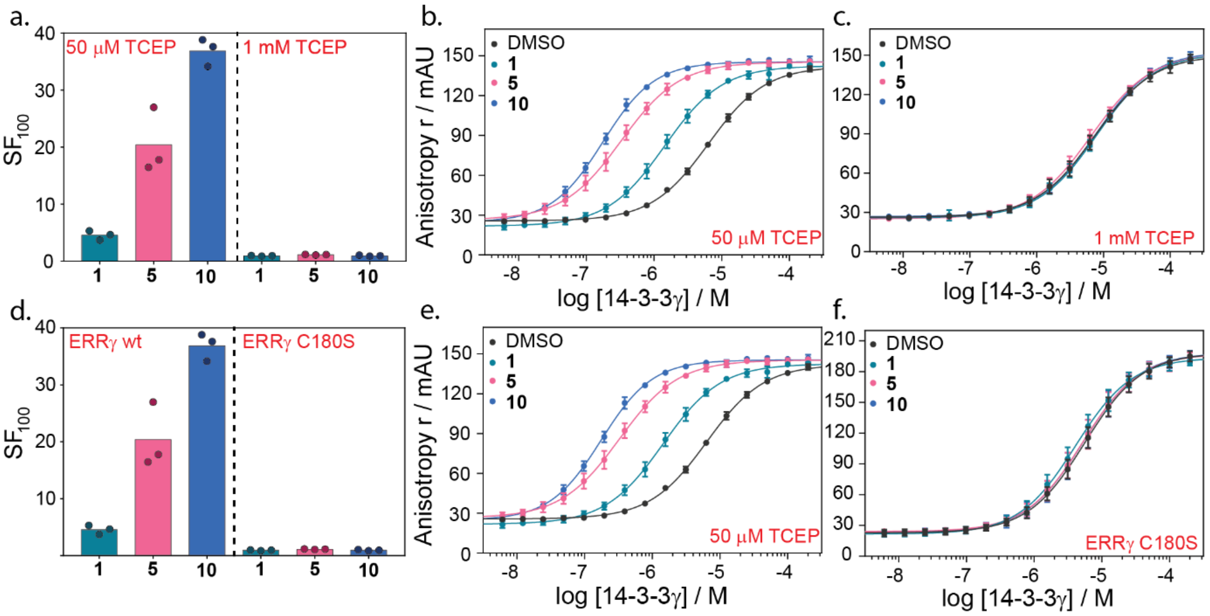

Figure S12. FA studies – effect TCEP. (a) Bar plot representation of stabilization factor 100 (SF_{100}) for compounds **1**, **5**, **10** with either 50 μM TCEP or 1 mM TCEP showing all stabilization is removed when using an excess of TCEP. (b-c) 14-3-3γ titrations to FITC-labelled ERRγ phosphopeptide in presence of 100 μM compound **1**, **5** and **10** with either 50 μM or 1 mM TCEP in the buffer. (d) Bar plot representation of stabilization factor 100 (SF_{100}) for compounds **1**, **5**, **10** on the 14-3-3/ERRγ wt complex for using a C180S mutant of the ERRγ peptide. (e-f) 14-3-3γ titrations to FITC-labelled ERRγ phosphopeptide (either wt or C180S mutant) in presence of 100 μM compound **1**, **5** and **10**.

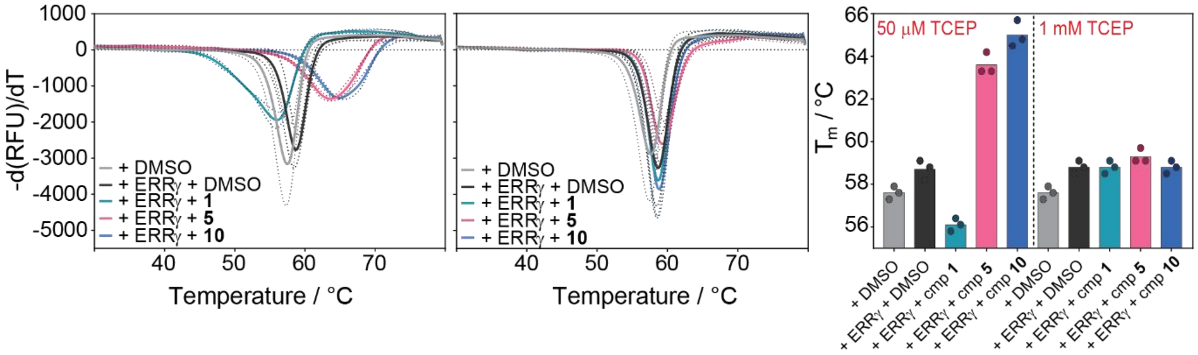

Figure S13. DSF studies – effect TCEP. Differential melting curves of 14-3-3γ in presence of ERRγ with DMSO or compounds **1**, **5** and **10** in presence of either 50 μM (left) or 1 mM TCEP (middle). And a bar plot representation of the identified melting temperature Tm for each of these samples.

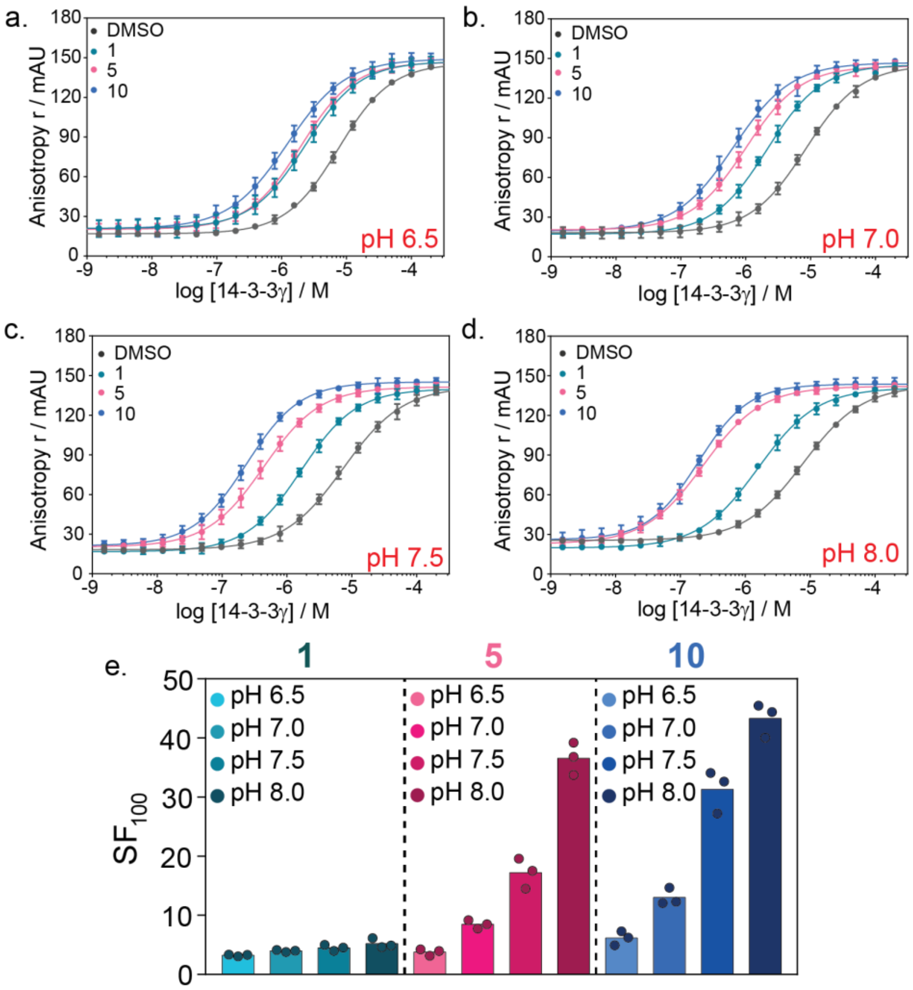

Figure S14. FA-studies – effect pH. (a-d) 14-3-3γ titrations to FITC-labelled ERRγ phosphopeptide in presence of 100 μM compound **1**, **5** and **10** after 24h incubation at four different pH's from 6.5 to 8.0. (b) Bar plot representation of SF_{100} for compounds **1**, **5**, and **10** when performing the FA studies at different pH values.

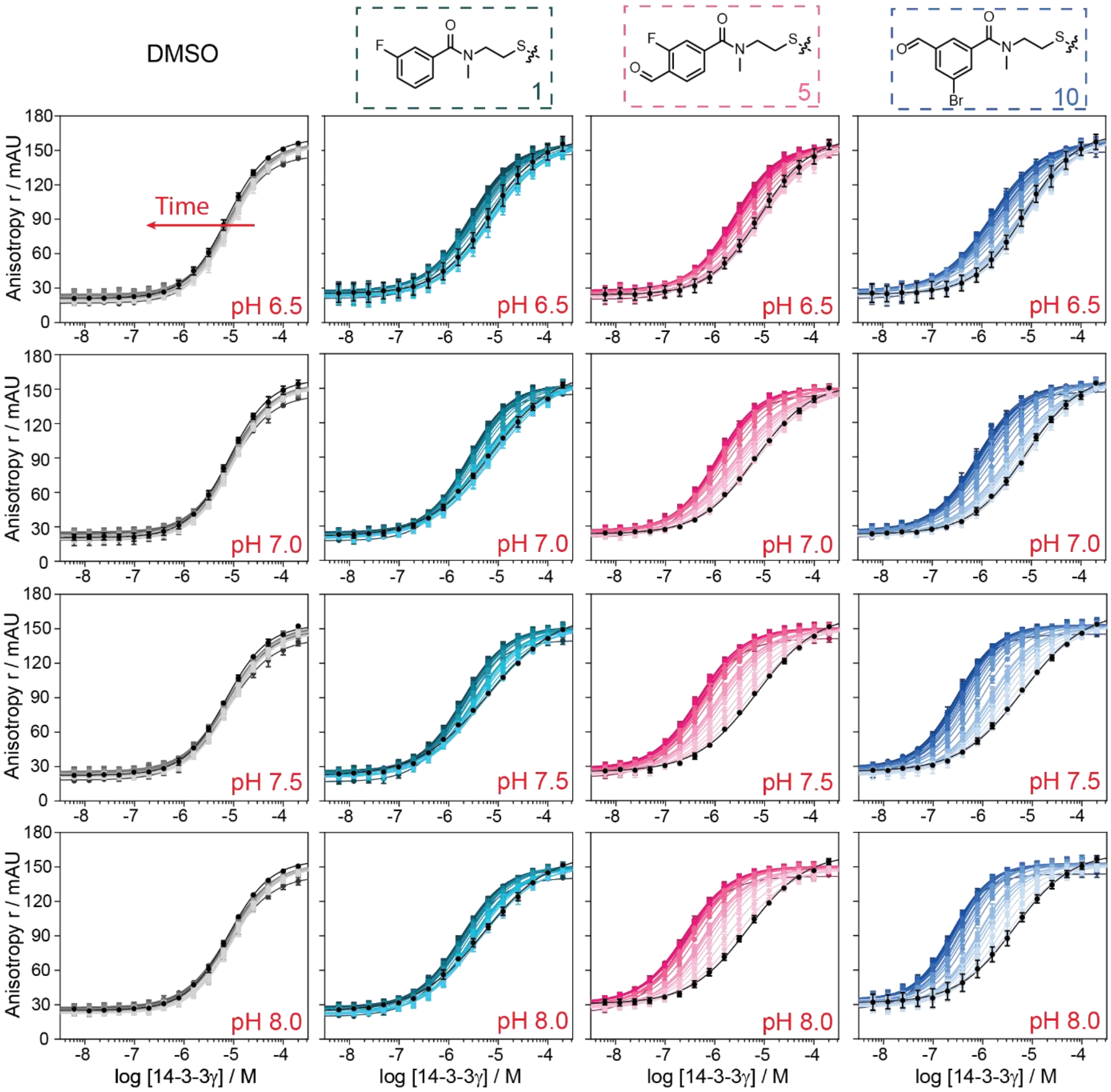

Figure S15. Time dependent FA at different pH. Time dependent results of 14-3-3γ titrations to FITC-labelled ERRγ phosphopeptide in presence of 100 μM compound **1**, **5** and **10** for a time course of 24 hours at four different pH's.

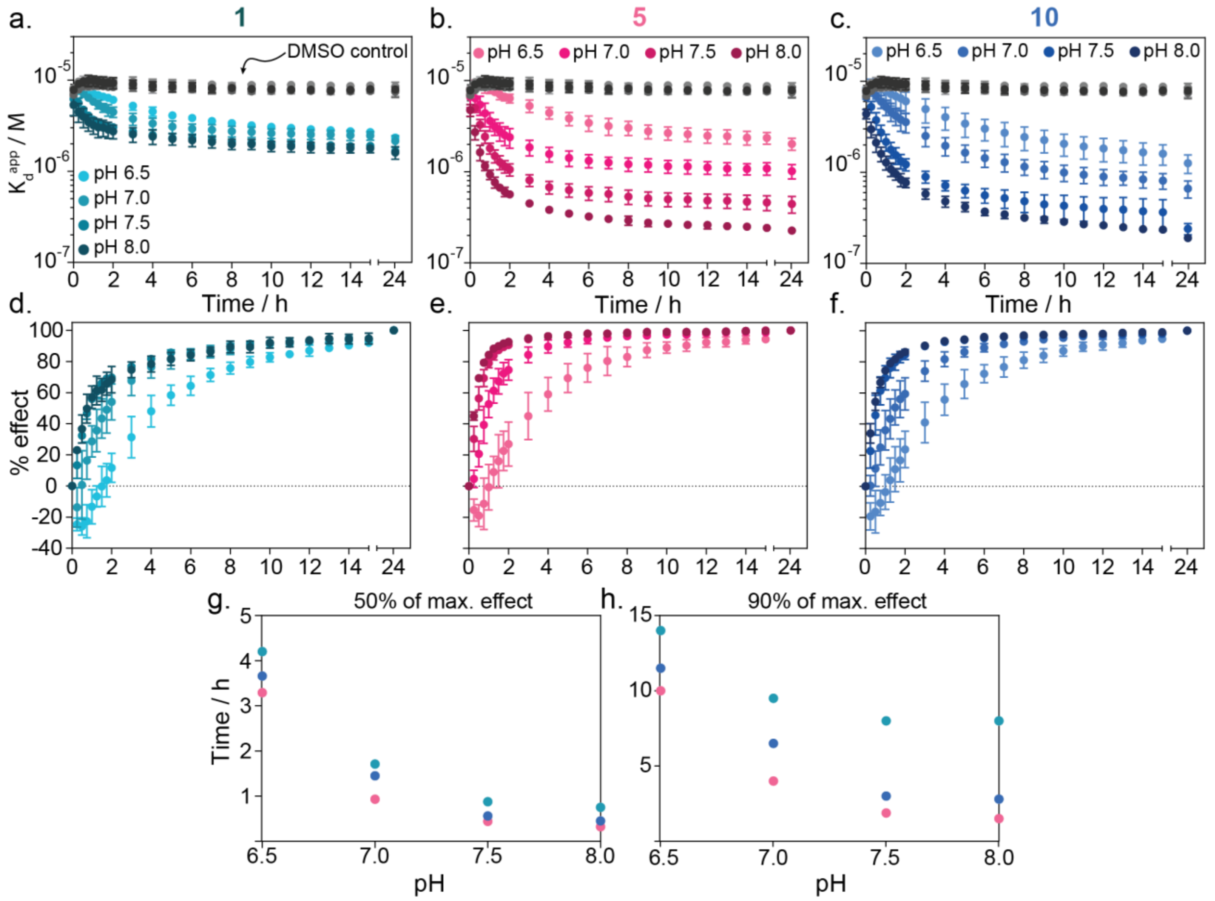

Figure S16. Time- and pH dependent FA-based apparent K_D analysis. (a-c) Determined apparent K_D from fluorescence anisotropy binding curves (see figure S15) and plotted over time for compounds **1**, **5** and **10**. Results show a time dependent decrease in K_D (increase in affinity) between 14-3-3γ and ERRγ as induced by the compounds over time. (d-f) Normalized plot of apparent K_D values as presented in a-c where the K_D at t=0h was set to no effect (0%) and the apparent K_D at t=24h as maximal effect (100%). (g-h) Time point of reaching 50 or 90% of the maximum effect of 14-3-3γ/ERRγ stabilization for compounds **1**, **5** and **10** at different pH's.

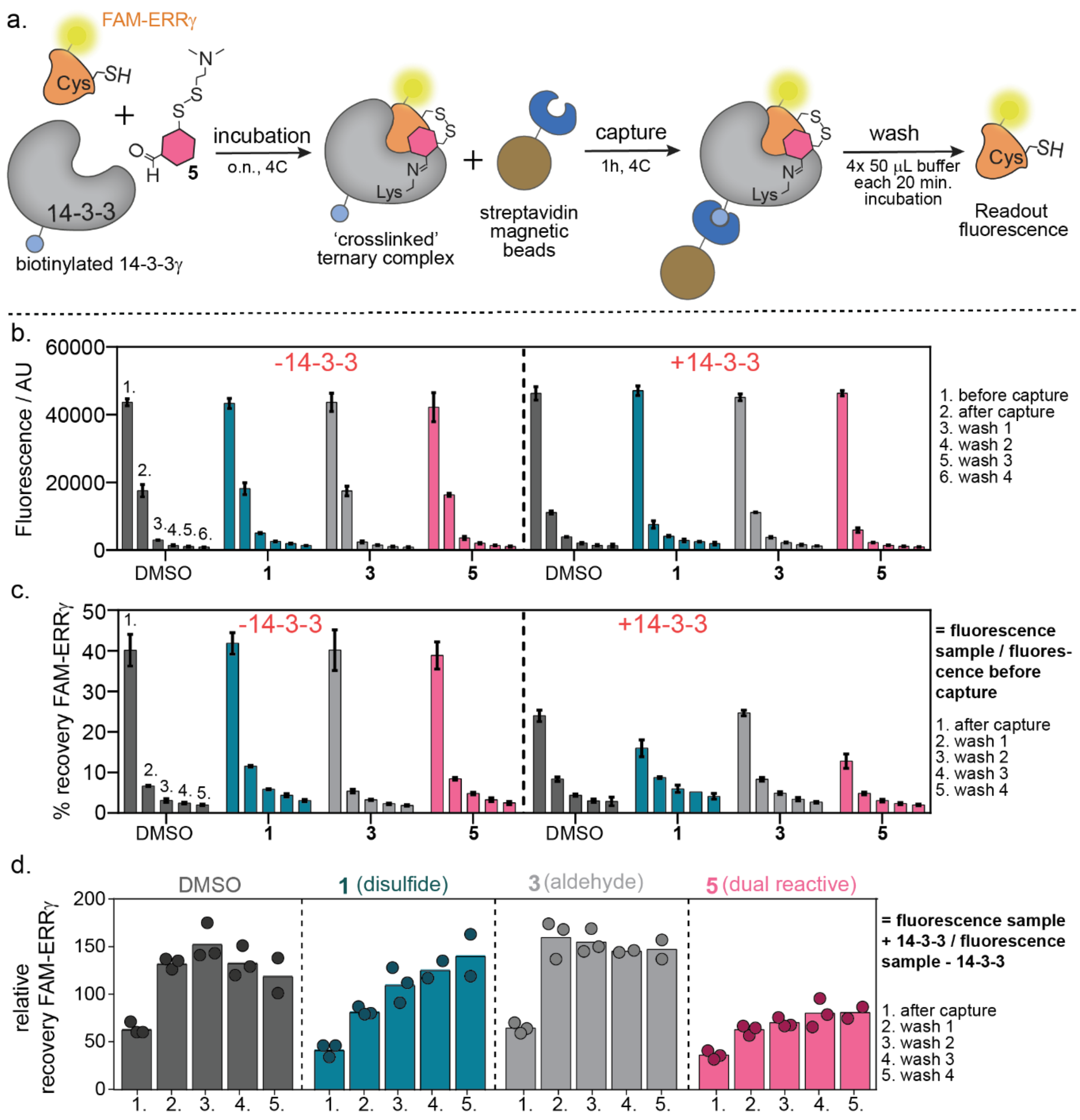

Figure S17. Washout experiment. (a) Schematic representation of washout experiment. Biotinylated 14-3-3γ (5 μM), fluorescein-labelled ERRγ 9-mer peptide (200 nM) and dual-reactive molecular lock **5** were preincubated overnight to form a ternary 'crosslinked' complex. This complex was immobilized on streptavidin-functionalized magnetic beads. Complex-functionalized beads were subsequently washed four times with 50 μL buffer. For each wash step, the complex was incubated 20 minutes in the buffer before removal to allow dissociation of the ERRγ peptide from the ternary complex. From samples before and after capturing, and from all wash fractions, we have measured the fluorescence to determine the amount of ERRγ peptide in each fraction. (b) Fluorescence signal of fluorescein-labelled ERRγ peptide after washout experiment. Samples are present for FAM-ERRγ incubated with and without biotinylated 14-3-3γ and in presence of either DMSO (negative control), disulfide compound **1**, aldehyde compound **3** or dual-reactive compound **5**. Fluorescence was measured from samples before and after capturing and from all wash fractions. (c) % recovery of FAM-ERRγ in each wash fraction of washout experiment. Percentage is obtained by dividing the fluorescence of each sample (see panel a) by the fluorescence signal obtained before capturing (input sample = 100%, see panel a). (d) Recovery of fluorescein labelled ERRγ 9-mer peptide in washout experiment from immobilized 14-3-3γ/ERRγ complex relative to recovery of fluorescein labelled ERRγ 9-mer peptide in absence of 14-3-3γ. Data shows recovery for **5** subsequent washing steps in presence of DMSO, or 100 μM compound **1**, **3** or **5**. Values are calculated by dividing the fluorescence signal (from panel a) of samples with 14-3-3 with the fluorescence signal of samples without 14-3-3, to correct for non-specific binding of peptide to the magnetic beads.

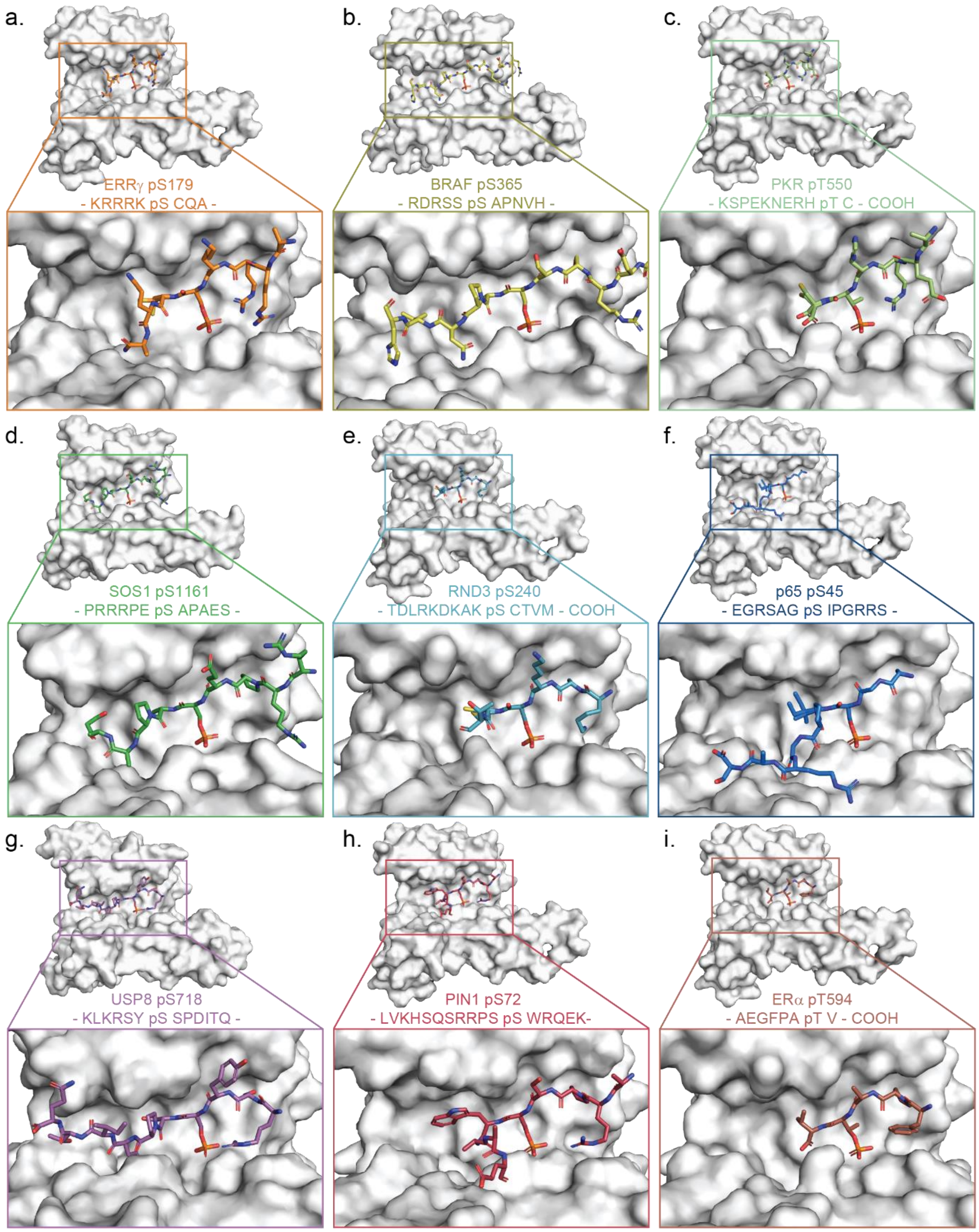

Figure S18. Selectivity pannel of 14-3-3 binders. Three dimensional structures of 14-3-3 (white surface) bound to (a) ERRγ pS179, PDB 6Y1D; (b) BRAF pS365, PDB 6NYB*; (c) PKR pT550, PDB 8B17; (d) SOS1 pS1161, PDB 6F08; (e) RND3 pS240, PDB 8BFC; (f) p65 pS46, PDB 6QHL; (g) USP8 pS718, PDB 6F09; (h) PIN1 pS72, PDB 7AOG; and (i) ERa pT594, PDB 4JC3. *This is a cryo-EM structure where all others are x-ray cyrstal structures.

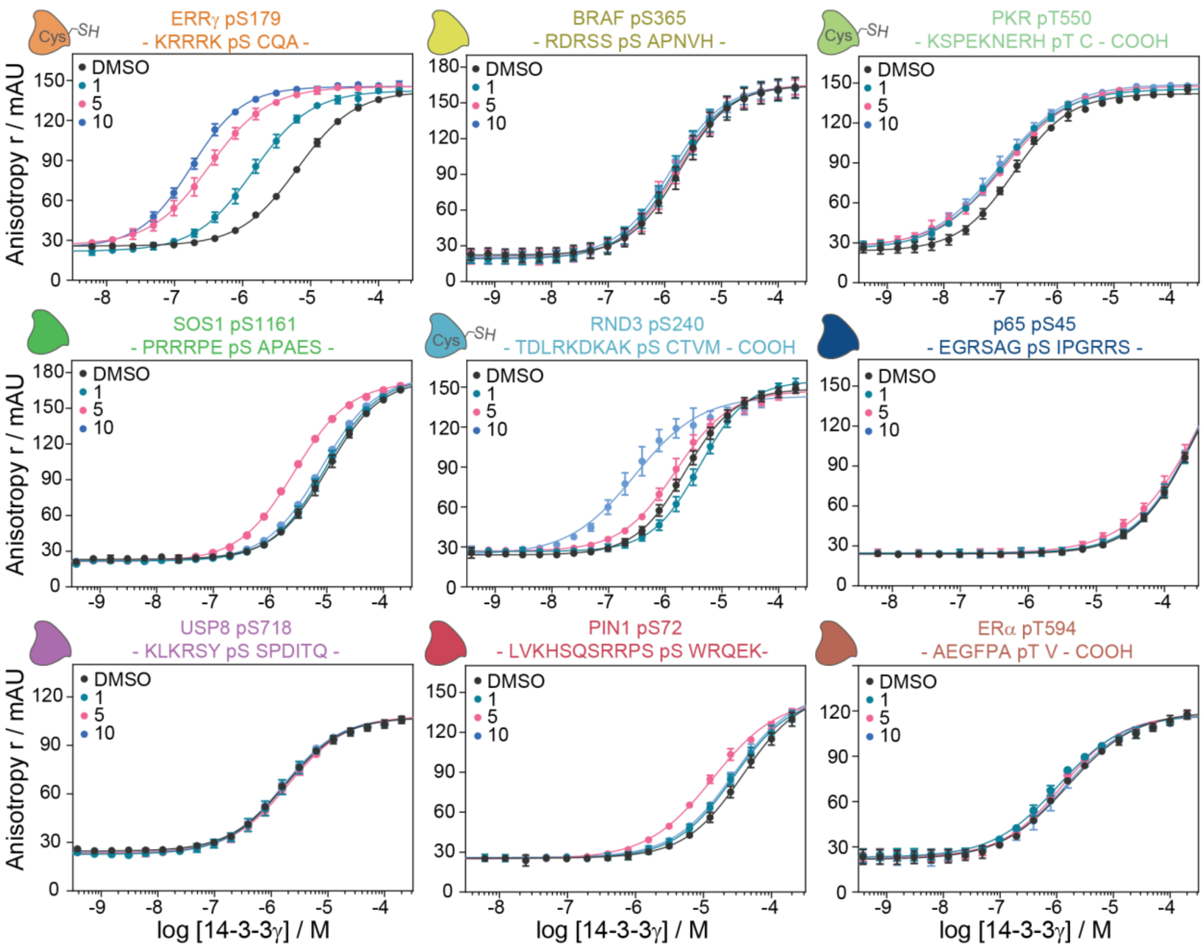

Figure S19. FA-based selectivity studies. FA results of 14-3-3γ titrations to nice fluorescein-labelled phosphopeptides in presence of 100 μM compound **1**, **5** and **10.**

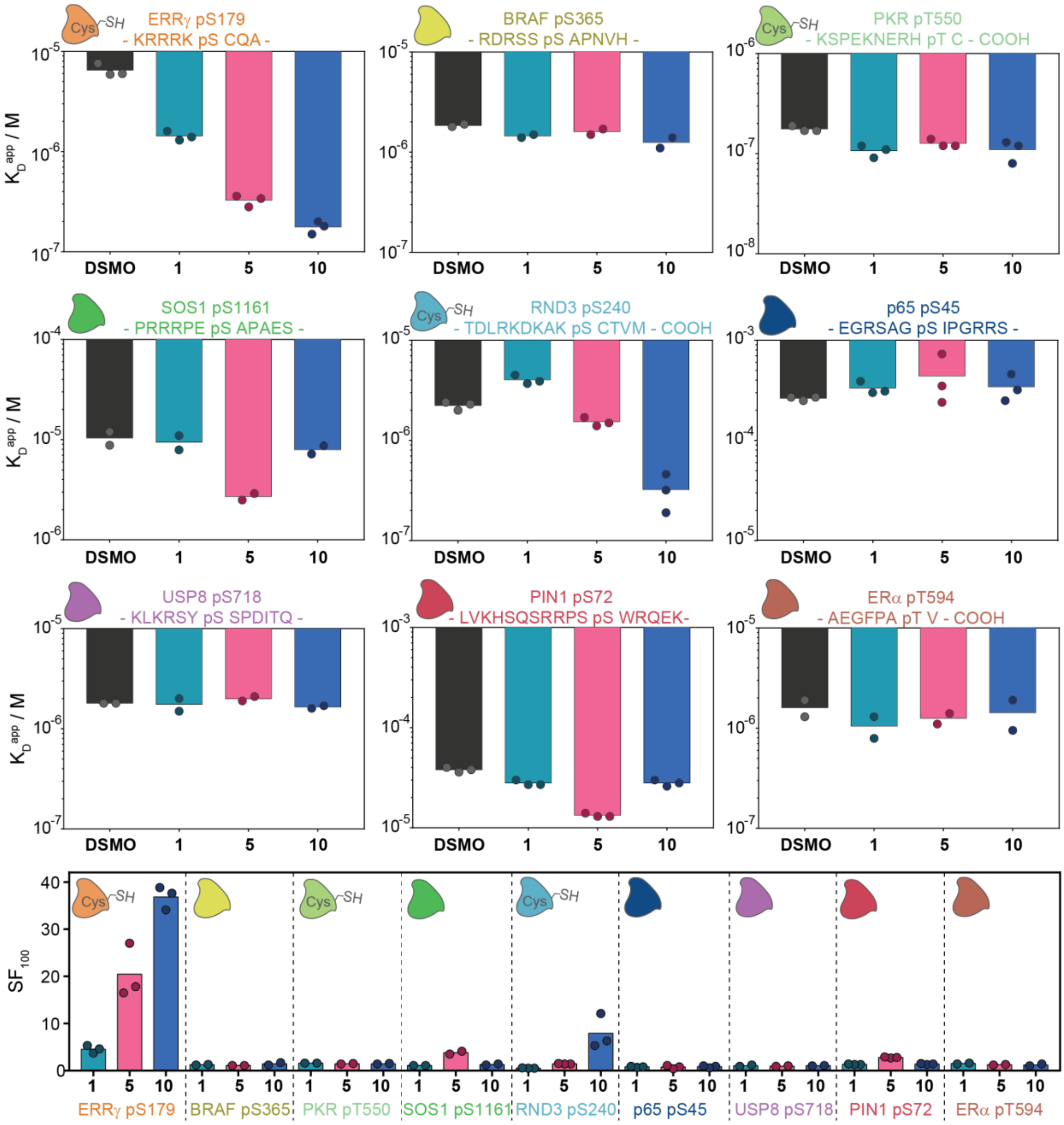

Figure S20. K_D and SF_{100} plot – selectivity screen. Bar plot representation of apparent K_D values of 14-3-3γ to nine different client peptides in presence of DMSO (negative control) or 100 μM of compound **1**, **5** or **10** (based on binding curves in Figure S19). Also a bar plot representation of stabilization factor (SF_{100}) of compounds **1**, **5** and **10** for each of these peptides (bottom plot).

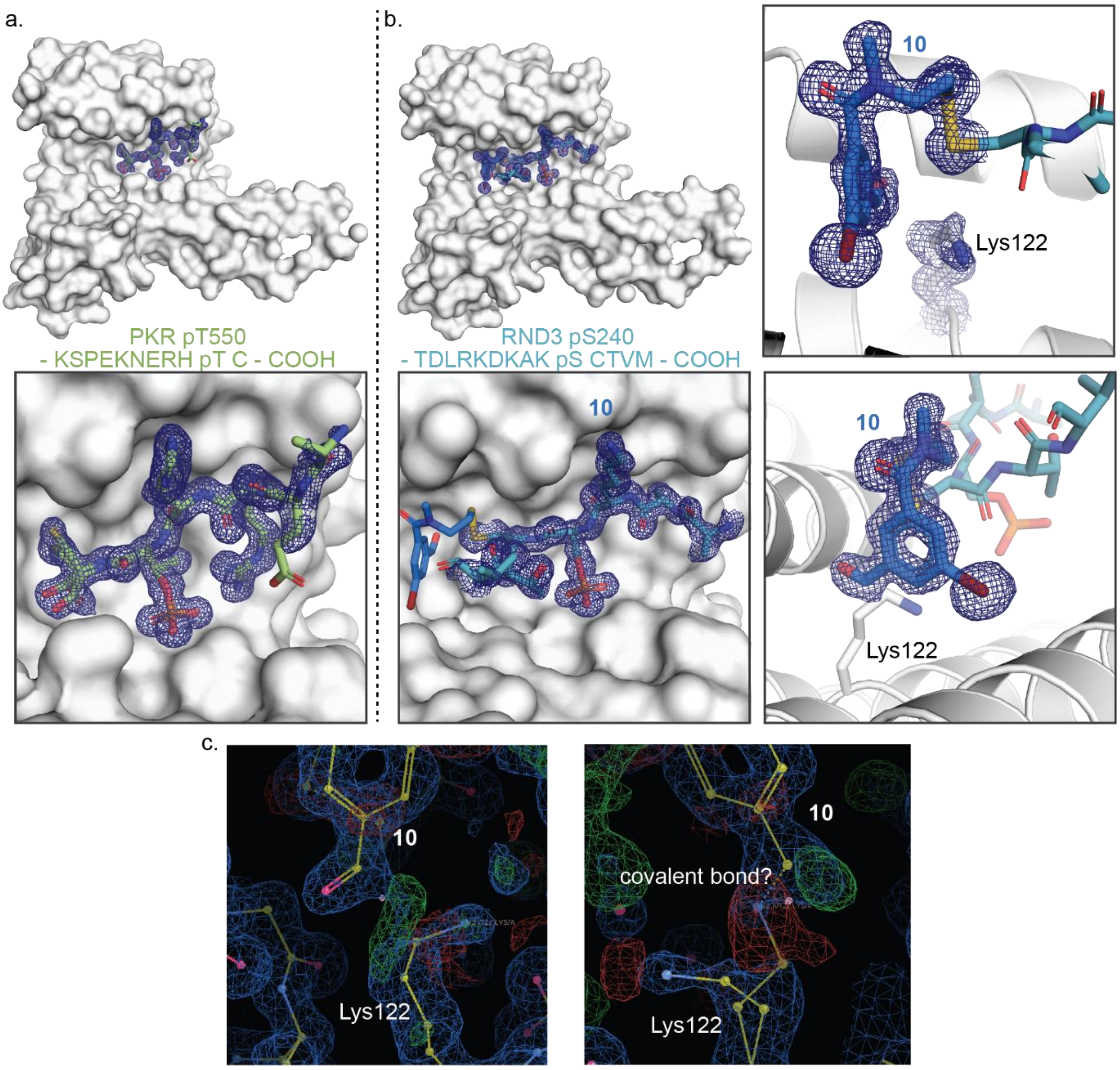

Figure S21. 14-3-3/PKR and 14-3-3/RND3/10 crystal structures. (a) Crystal structure of 14-3-3σ (white surface) bound to PKR phosphopeptide (green). The 2Fo – Fc electron density map (blue mesh) is contoured at 1σ. (b) Crystal structure of 14-3-3σ (white surface) bound to RND3 phosphopeptide (green) and molecular lock **10**. Several perspectives are shown with electron density for the peptide, ligand and lysine 122. The 2Fo – Fc electron density map (blue mesh) is contoured at 1σ. (c) Electron density map and model of 14-3-3/RND3/**10** crystal structures as represented in data processing software coot. Clear electron density is observed for the aldehyde moiety (left) and non-bounded Lys122. Upon attempt of linking fragment and Lys122 clearly no density is present (right) indicating major part of compound **10** is unbound to Lys122. The 2Fo – Fc electron density map (blue mesh) is contoured at 1σ.

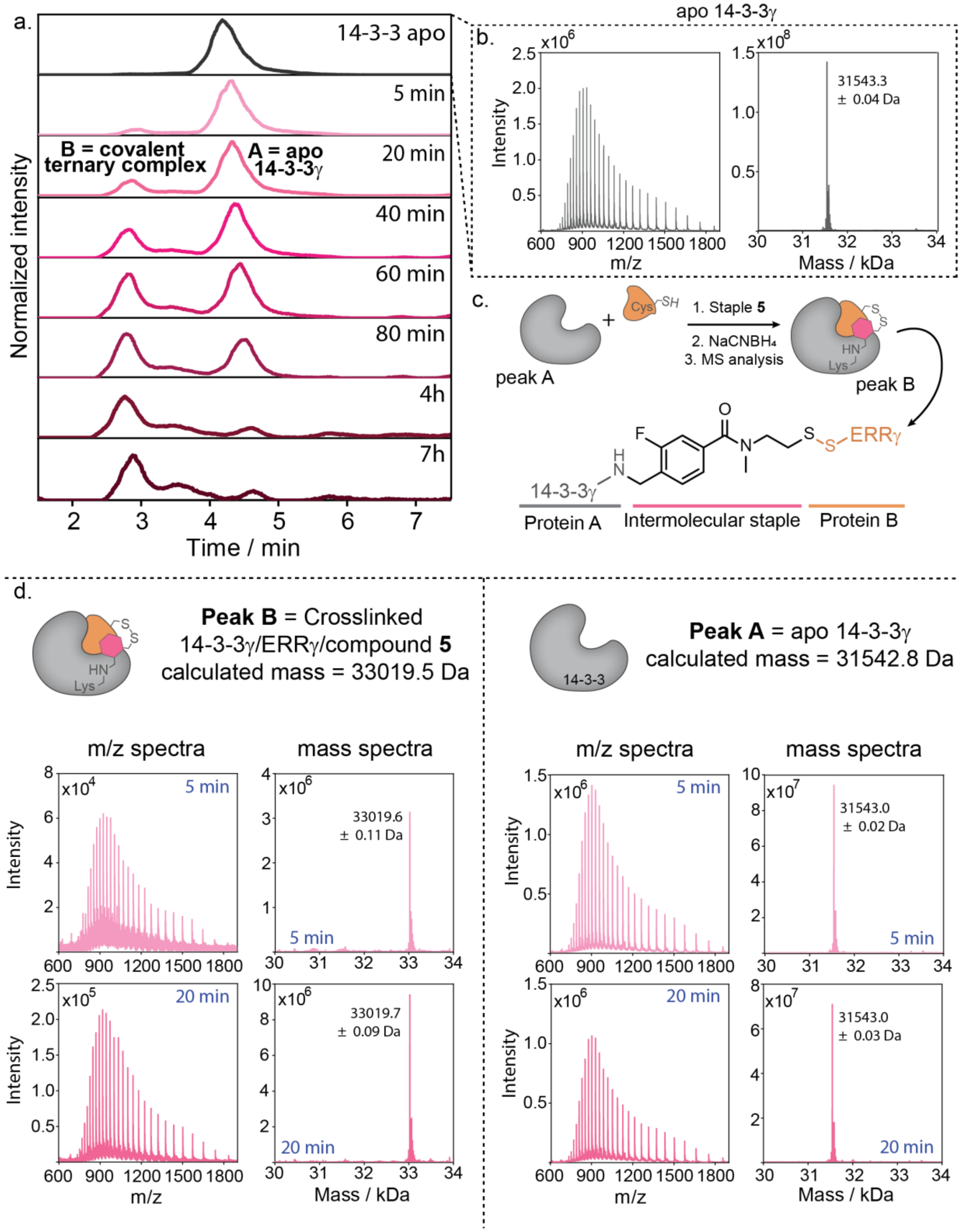

Figure S22a. QTOF-MS analysis of 14-3-3/ERRg/5 ternary complex formation. LC-MS analysis of time-dependent crosslinking experiment of 14-3-3γ (5 μM) and ERRγ (10 μM) phosphopeptide by compound **5** (50 μM). (a) LC-chromatograms of each sample. (b) m/z spectrum and associated MS spectrum of apo 14-3-3γ sample (Calculated mass 14-3-3γ apo: 31542.8 Da). (c) Schematic representation of experiment in which 14-3-3γ and ERRγ phosphopeptide are incubated with compound 5 for x amount of time. After incubation, the imine bond is reduced using 1000x $NaBH_3CN$ for 15 minutes. The samples are subsequently measured using LC-MS. (d) m/z spectra and mass associated mass spectra of peak B (retention time: 2.75-3.25 min) and peak A (retention time: 4.00-5.00 min) in chromatograms of samples after 5 and 20 minutes incubation. Calculated mass 14-3-3γ apo: 31542.8 Da. Calculated mass covalently crosslinked 14-3-3γ/ERRγ/**5** complex: 33019.5 Da.

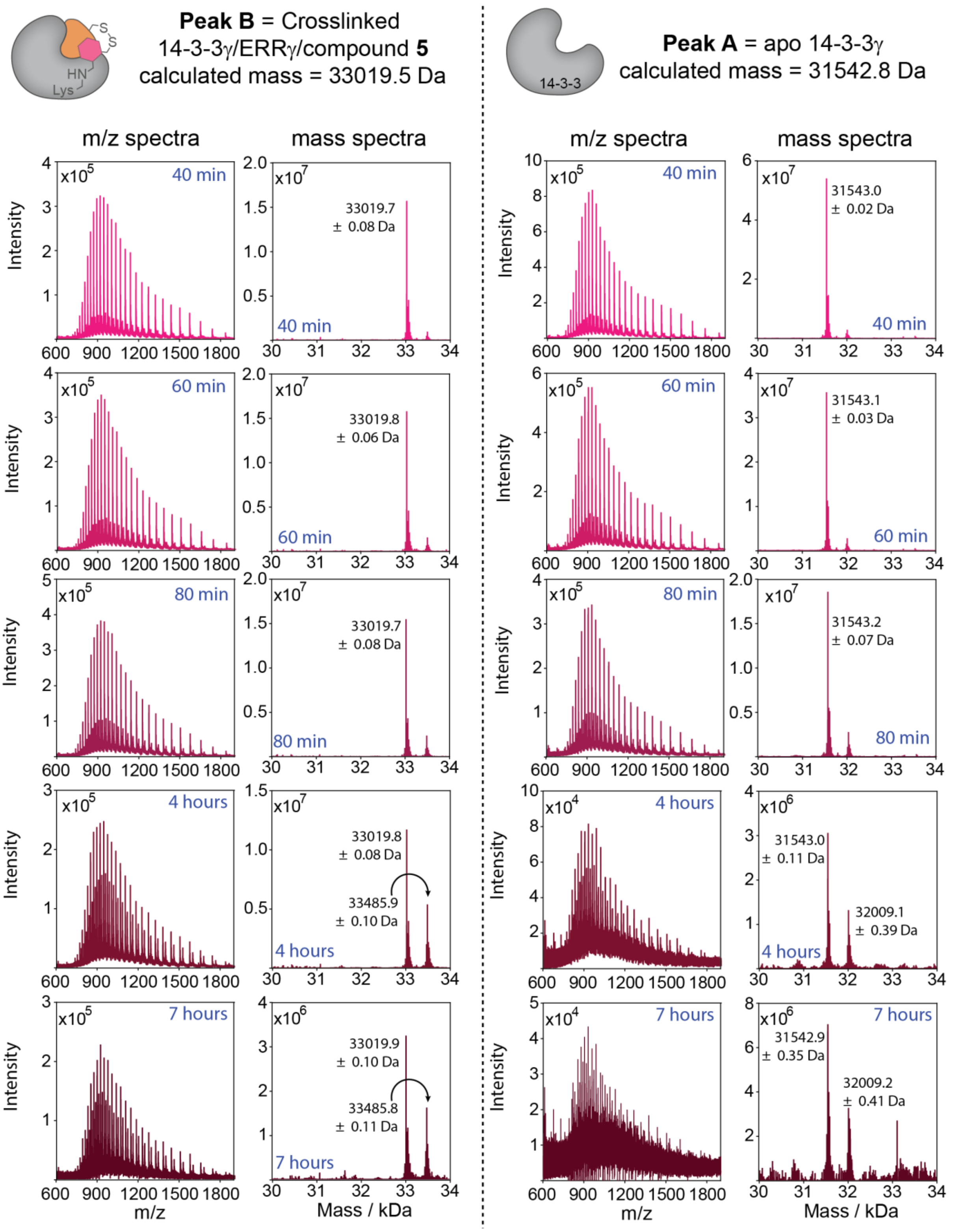

Figure S22b. QTOF-MS analysis of 14-3-3/ERRg/5 ternary complex formation. m/z spectra and associated mass spectrum of both peak A (retention time: 2.75-3.25 min) and peak B (retention time: 4.00-5.00 min) in chromatograms of Figure S22a with an incubation time between 5 minutes and 7 hours. Calculated mass 14-3-3γ apo: 31542.8 Da; Calculated mass covalently crosslinked 14-3-3γ/ERRγ/**5** complex: 33019.5 Da.

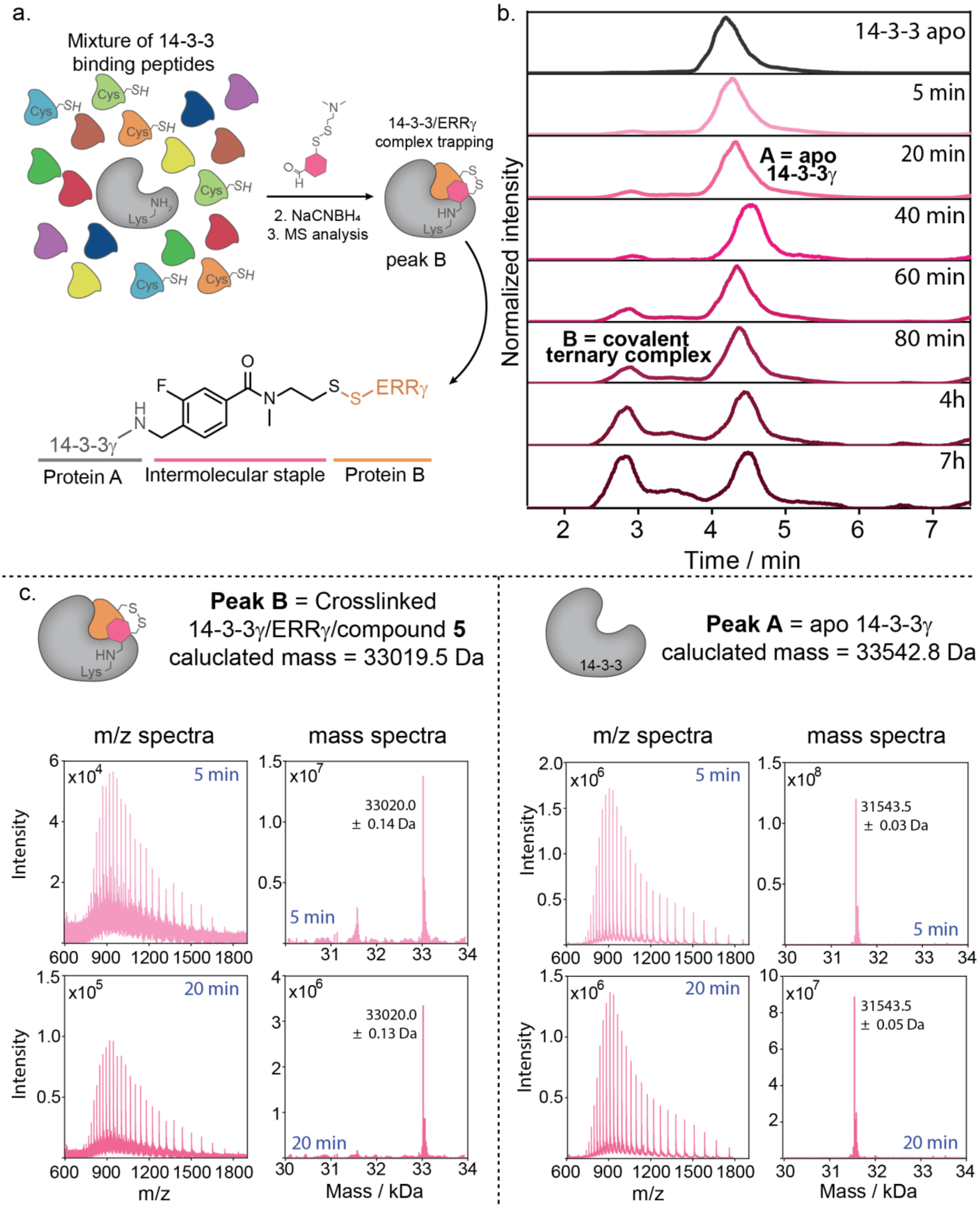

Figure S23a. QTOF-MS analysis of 14-3-3/peptide mix/5. LC-MS analysis of time-dependent crosslinking experiment with a mixture of 14-3-3γ (5 μM) nine different phosphopeptides (10 μM each) including ERRγ pS179 and compound **5** (50 μM). (a) Schematic representation of experiment in which 14-3-3γ and ERRγ are crosslinked using compound **5** when in presence of a mixture of peptides. (b) LC-chromatograms of each sample after x amount of incubation time. (c) m/z spectra and mass associated mass spectra of peak B (2.50-3.25 min) and peak 2 (3.75-5.00 min) in chromatograms of samples after 5 and 20 minutes incubation. Calculated mass 14-3-3γ apo: 31542.8 Da. Calculated mass covalently crosslinked 14-3-3γ/ERRγ/**5** complex: 33019.5 Da.

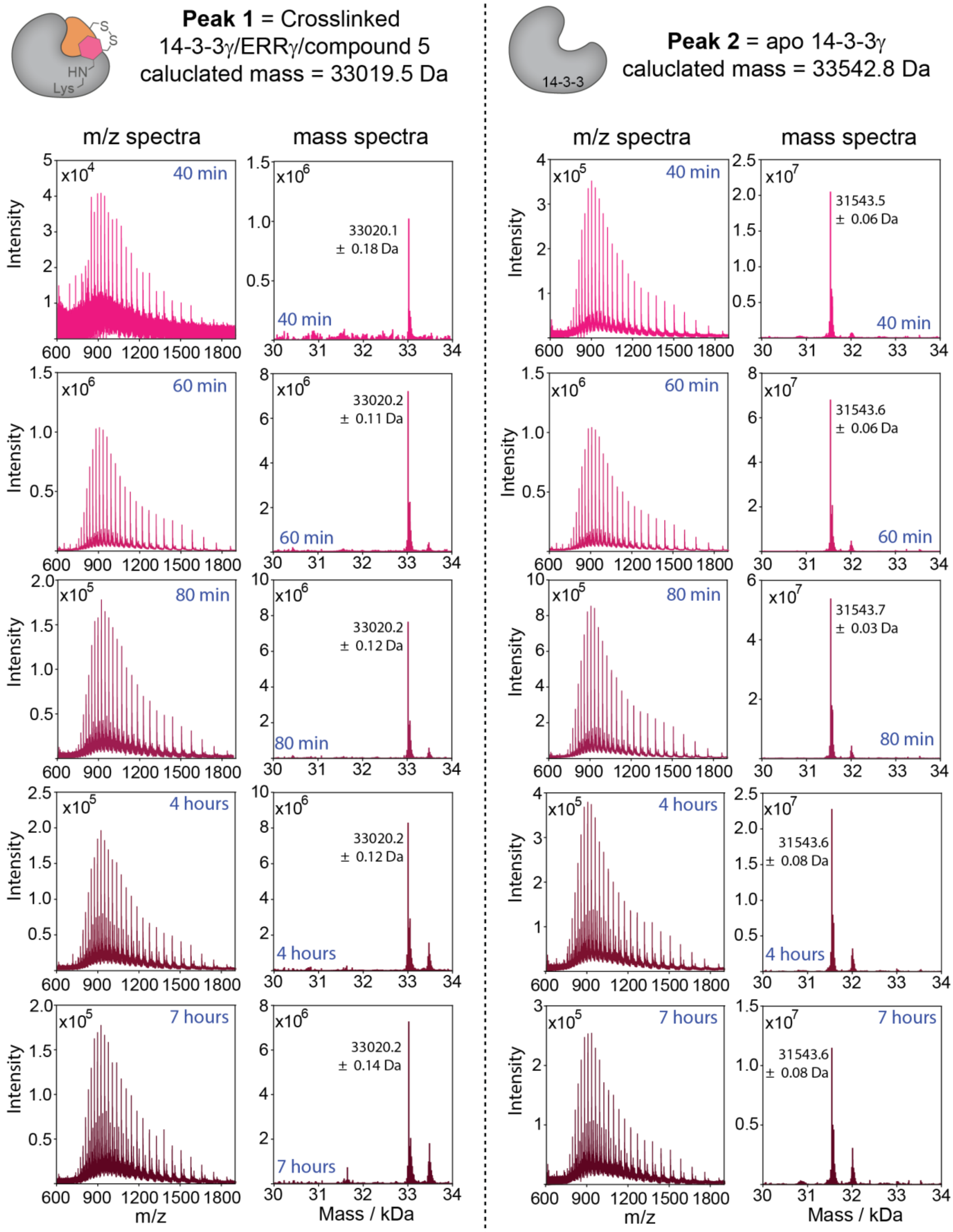

Figure S23b. QTOF-MS analysis of 14-3-3/peptide mix/5. m/z spectra and associated mass spectrum of both peak A (retention time: 2.50-3.25 min) and peak B (retention time: 3.75-5.00 min) in chromatograms of Figure S23a with an incubation time between 5 minutes and 7 hours. Calculated mass 14-3-3γ apo: 31542.8 Da; Calculated mass covalently crosslinked 14-3-3γ/ERRγ/**5** complex: 33019.5 Da.

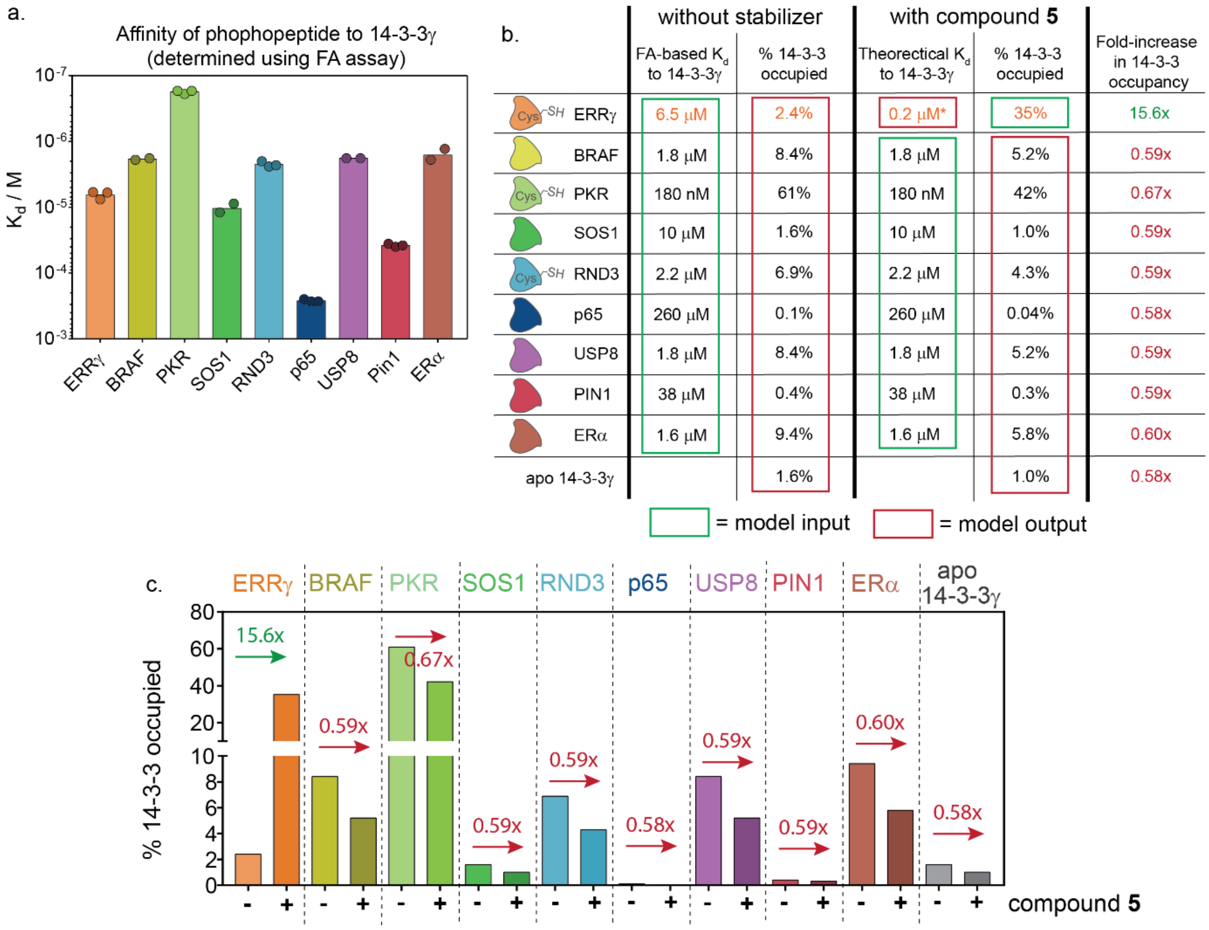

	without stabilizer		with compound **5**		
	FA-based K_d to 14-3-3γ	% 14-3-3 occupied	Theorectical K_d to 14-3-3γ	% 14-3-3 occupied	Fold-increase in 14-3-3 occupancy
Cys-SH ERRγ	6.5 μM	2.4%	0.2 μM*	35%	15.6x
BRAF	1.8 μM	8.4%	1.8 μM	5.2%	0.59x
Cys-SH PKR	180 nM	61%	180 nM	42%	0.67x
SOS1	10 μM	1.6%	10 μM	1.0%	0.59x
Cys-SH RND3	2.2 μM	6.9%	2.2 μM	4.3%	0.59x
p65	260 μM	0.1%	260 μM	0.04%	0.58x
USP8	1.8 μM	8.4%	1.8 μM	5.2%	0.59x
PIN1	38 μM	0.4%	38 μM	0.3%	0.59x
ERα	1.6 μM	9.4%	1.6 μM	5.8%	0.60x
apo 14-3-3γ		1.6%		1.0%	0.58x

(green box) = model input (red box) = model output

Figure S24. QTOF-MS analysis of 14-3-3/ERRγ/5 ternary complex formation. (a) Bar plot representation of affinity (K_D) of nine phosphopeptides to 14-3-3γ as determine in fluorescence anisotropy assays (see Figure S17-18). (b) Values determined by thermodynamic model[1] of 14-3-3 occupancy by each of the nine peptides when incubated as mixture of 5 μM 14-3-3 and 10 μM of each peptide. First column describes the K_D values (model input) obtained from FA studies, which provides (model output) the 14-3-3 occupancy percentages for each peptide in the second column. Column three and four show similar results however now K_D values are used for all peptides except ERRγ as model input, together with the 35% 14-3-3 occupancy by ERRγ upon stabilization with compound **5**. Final column shows the fold-increase in 14-3-3 occupancy by each of the peptide partners upon increase of ERRg affinity. (c) Bar plot representation of % 14-3-3 occupancy by each of the 14-3-3 binding peptide when using a complex mixture as calculated by thermodynamic model. Occupancy are shown for a non-stabilized environment and in presence of compound **5**. Furthermore the fold change in 14-3-3 occupancy is shown for each species.

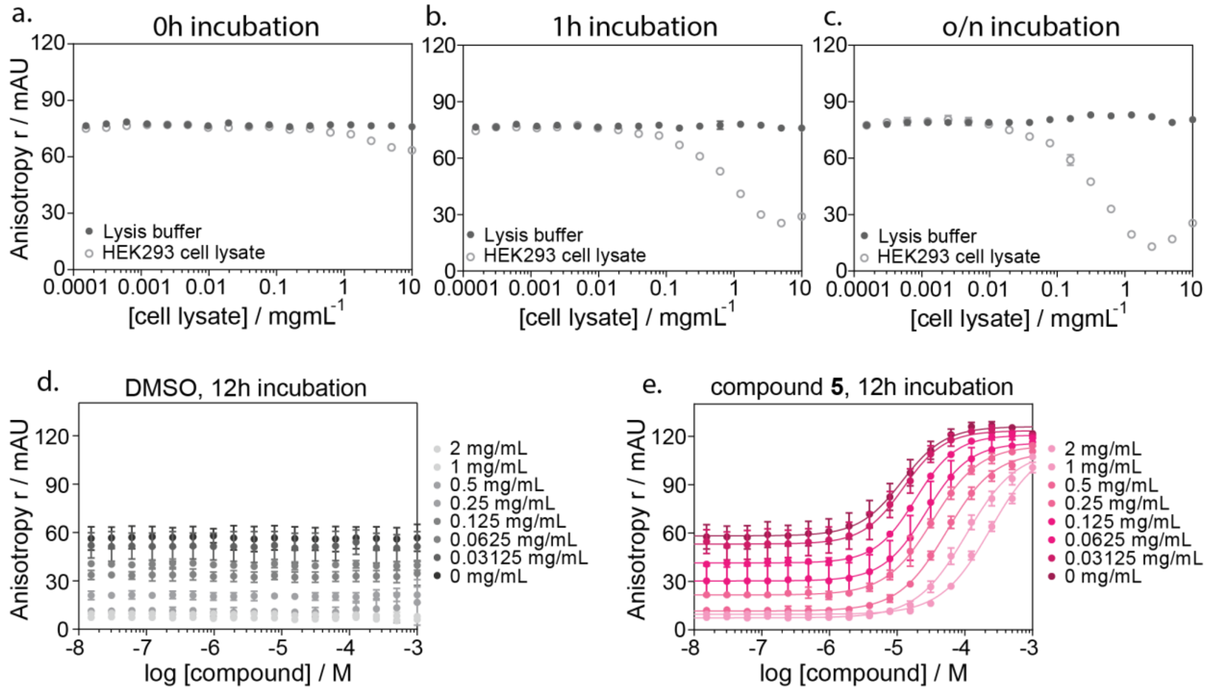

Figure S25. FA compound titrations with cell lysate. (a-c) HEK293 cell lysate (or lysis buffer) titration to preformed complex of 14-3-3γ (5 μM, 0.16 mg/mL) and fluorescein labelled ERRγ peptide (100 nM). Data is shown after 0h (a), 1h (b), and overnight (c) incubation. (d-e) Fluorescence anisotropy studies of compound **5** (right) titrations or DMSO as negative control (left) to a preformed complex of 14-3-3γ (5 μM, 0.16 mg/mL) and fluorescein labelled ERRγ peptide (100 nM) at various concentrations HEK293 cell lysate (up to 2 mg/mL). Data is shown after 12h of incubation at room temperature.

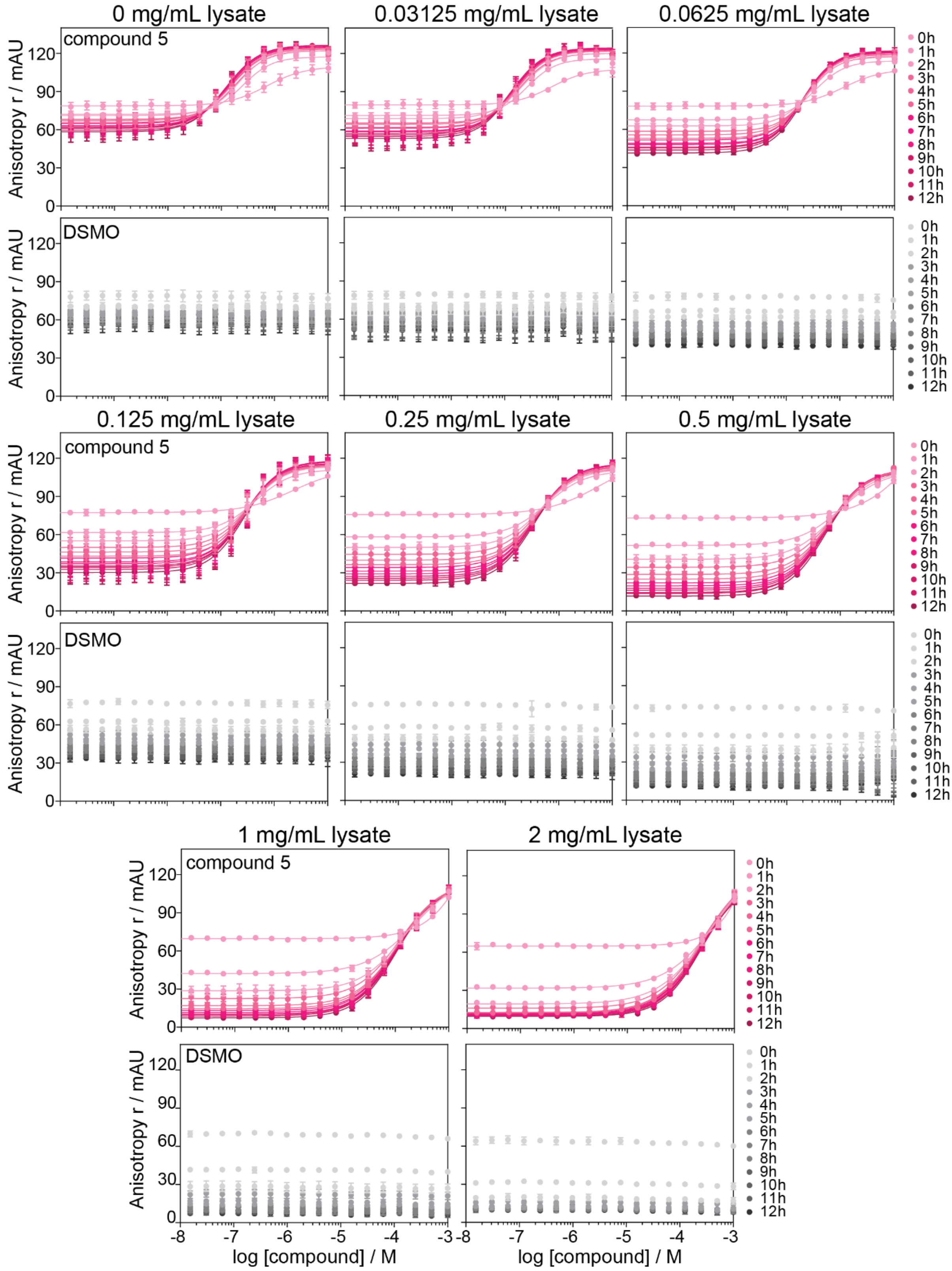

Figure S26. Time-dependent FA compound titrations with cell lysate. Fluorescence anisotropy studies of compound **5** titrations (or DMSO as negative control) to a preformed complex of 14-3-3γ (5 μM) and fluorescein labelled ERRγ peptide (100 nM). Assay is performed in presence of various concentrations HEK293 cell lysate (up to 2 mg/mL) and is measured over time (every hour for 12 hours).

Table S3. Data collection and refinement statistics of crystal structures

**PDB**	**8B2I**	**8B4Q**	**8BJN**
Protein	14-3-3σΔc C38N	14-3-3σΔc C38N	14-3-3σΔc C38N
Peptide	ERRγ pS179 9-mer	ERRγ pS179 9-mer	ERRγ pS179 9-mer
Compound	**4**	**5**	**6**
Beam	DESY p11	DESY p11	DESY p11
Data collection			
Wavelength (Å)	1.0332	1.0332	1.0332
Space group	C 2 2 21	C 2 2 21	C 2 2 21
Cell dimensions			
a, b, c (Å)	82.6 112.0 62.6	82.6 112.2 62.5	82.5 112.3 62.6
α, β, γ (°)	90, 90, 90	90, 90, 90	90, 90, 90
Resolution (Å)	45.57 – 1.40 (1.42 – 1.40)	45.55 – 1.40 (1.42 – 1.40)	45.56 – 1.40 (1.43 – 1.40)
I / σ(I)	27.7 (9.3)	34.7 (9.0)	37.2 (9.8
Completeness (%)	99.7 (99.4)	99.4 (96.0)	99.8 (98.6)
Redundancy	13.3 (12.2)	13.4 (12.3)	13.2 (12.2)
$CC_{1/2}$	0.999 (0.985)	1.000 (0.984)	1.000 (0.990)
Refinement			
No. reflections	57056	56939	57123
R_{work}/R_{free}	0.164/0.178	0.166/0.175	0.168/0.176
No. atoms			
Protein	2047	2016	1986
Ligand/ion	19	19	19
Water	327	335	316
B-factors			
Protein	13.42	13.07	13.32
Ligand/ion	16.60	16.06	19.79
Water	25.74	25.48	24.49
R.m.s. deviations			
Bond lengths (Å)	0.006	0.006	0.006
Bond angles (°)	0.92	0.89	0.91
Ramachandran			
favored (%)	98.73	99.15	98.28
outliers (%)	0.00	0.00	0.00

**PDB**	**8BM5**	**8BJG**	**8B5P**
Protein	14-3-3σΔc C38N	14-3-3σΔc C38N	14-3-3σΔc C38N
Peptide	ERRγ pS179 9-mer	ERRγ pS179 9-mer	ERRγ pS179 9-mer
Compound	**7**	**8**	**10**
Beam	DESY p11	DESY p11	DESY p11
Data collection			
Wavelength (Å)	1.0332	1.0332	1.0332
Space group	C 2 2 21	C 2 2 21	C 2 2 21
Cell dimensions<br>a, b, c (Å)<br>α, β, γ (°)	 82.6 112.0 62.6<br>90, 90, 90	 82.4 112.2 62.5<br>90, 90, 90	 82.5 111.8 62.5<br>90, 90, 90
Resolution (Å)	45.59 – 1.40 (1.42 – 1.40)	45.53 – 1.40 (1.43 – 1.40)	45.51 – 1.40 (1.43 – 1.40)
I / σ(I)	36.8 (9.8)	33.1 (8.8)	33.6 (7.7)
Completeness (%)	99.8 (98.6)	99.7 (95.5)	99.5 (96.9)
Redundancy	13.4 (12.5)	13.3 (12.0)	13.4 (12.7)
$CC_{1/2}$	1.000 (0.989)	0.999 (0.981)	1.000 (0.973)
Refinement			
No. reflections	57210	57005	56711
R_{work}/R_{free}	0.166/0.186	0.166/0.176	0.170/0.185
No. atoms<br>Protein<br>Ligand/ion<br>Water	 1991<br>20<br>323	 2023<br>20<br>346	 1945<br>19<br>348
B-factors<br>Protein<br>Ligand/ion<br>Water	 14.46<br>20.87<br>26.28	 13.12<br>15.38<br>25.36	 12.97<br>14.58<br>25.34
R.m.s. deviations<br>Bond lengths (Å)<br>Bond angles (°)	 0.006<br>0.92	 0.006<br>0.88	 0.006<br>0.91
Ramachandran<br>favored (%)<br>outliers (%)	 98.73<br>0.00	 98.73<br>0.00	 98.73<br>0.00

PDB	8BFC	8B2K	8B17
Protein	14-3-3σΔc C38N	14-3-3σΔc C38N	14-3-3σΔc
Peptide	RND3 pS240<br>14-mer	RND3 pS240<br>14-mer	PKR pT550 11-mer
Compound	-	**10**	-
Beam	DESY p11	DESY p11	DESY p11
Data collection			
Wavelength (Å)	1.0332	1.0332	1.0332
Space group	C 2 2 21	C 2 2 21	C 2 2 21
Cell dimensions<br>a, b, c (Å)<br>α, β, γ (°)	 82.4 112.4 62.7<br>90, 90, 90	 82.4 112.2 62.6<br>90, 90, 90	 82.7 112.5 62.6<br>90, 90, 90
Resolution (Å)	45.59-1.40 (1.42-1.40)	45.56 – 1.40 (1.43 – 1.40)	45.64 – 1.40 (1.43 – 1.40)
$I / \sigma(I)$	15.2 (3.9)	41.5 (17.6)	36.4 (7.1)
Completeness (%)	99.7 (95.0)	99.9 (99.2)	99.7 (98.0)
Redundancy	5.3 (4.9)	13.2 (12.2)	12.9 (12.0)
$CC_{1/2}$	0.996 (0.914)	0.999 (0.995)	0.999 (0.984)
Refinement			
No. reflections	57286	57149	57185
R_{work}/R_{free}	0.164/0.1721	0.167/0.179	5596
No. atoms<br>Protein<br>Ligand/ion<br>Water	 2039<br>5<br>286	 1980<br>20<br>324	 1974<br>4<br>330
B-factors<br>Protein<br>Ligand/ion<br>Water	 14.48<br>15.63<br>25.96	 13.37<br>18.44<br>25.97	 14.64<br>19.16<br>25.92
R.m.s. deviations<br>Bond lengths (Å)<br>Bond angles (°)	 0.006<br>0.89	 0.006<br>0.92	 0.006<br>0.89
Ramachandran<br>favored (%)<br>outliers (%)	 98.30<br>0.00	 98.31<br>0.00	 98.73<br>0.00

Experimental Section Biophysical Assays

14-3-3γ protein expression (FA, TSA, MS assays)

A pPROEX HTb expression vector encoding the human 14-3-3 protein gamma (14-3-3γ) with a N-terminal his_6-tag was transformed by heat shock into NiCo21 (DE3) competent cells. Single colonies were cultured in 50 mL LB medium (100 μg/mL ampicillin). After overnight incubation at 37 °C, cultures were transferred to 2 L TB media (100 μg/mL ampicillin, 1 mM $MgCl_2$) and incubated at 37 °C until an OD600 nm of 0.8-1.2 was reached. Protein expression was then induced with 0.4 mM isopropyl-β-d-thiogalactoside (IPTG), and cultures were incubated overnight at 18˚C. Cells were harvested by centrifugation (8600 rpm, 20 minutes, 4˚C) and resuspended in lysis buffer (50 mM Hepes, pH 8.0, 300 mM NaCl, 12.5 mM imidazole, 5 mM $MgCl_2$, 2 mM βME) containing cOmplete™ EDTA-free Protease Inhibitor Cocktail tablets (1 tablet/100 ml lysate) and benzonase (5 μl/100 ml). After lysis using a C3 Emulsiflex-C3 homogenizer (Avestin), the cell lysate was cleared by centrifugation (20000 rpm, 30 minutes, 4˚C) and purified using Ni^{2+}-affinity chromatography (Ni-NTA superflow cartridges, Qiagen). Typically two 5 mL columns (flow 5 mL/min) were used for a 2 L culture in which the lysate was loaded on the column, washed with 10 CV wash buffer (50 mM Hepes, pH 8.0, 300 mM NaCl, 25 mM imidazole, 2 mM βME), and eluted in several fractions (2-4 CV) of elution buffer (50 mM Hepes, pH 8.0, 300 mM NaCl, 250 mM imidazole, 2 mM βME). Fractions containing the 14-3-3 protein were combined and dialyzed into 25 mM HEPES pH 8.0, 100 mM NaCl, 10 mM $MgCl_2$, 500 μM TCEP. Finally, the protein was concentrated to ~60 mg/mL, analyzed Q-Tof LC/MS, and aliquots were flash-frozen for storage at −80 °C.

14-3-3σΔc C38N protein expression (Crystallography)

A pPROEX HTb expression vector encoding the human 14-3-3 protein sigma truncated after T231 (14-3-3σΔc), mutated C38N, and containing a N-terminal his_6-tag was transformed by heat shock into NiCo21 (DE3) competent cells. Single colonies were cultured in 50 mL LB medium (100 μg/mL ampicillin). After overnight incubation at 37 °C, cultures were transferred to 2 L TB media (100 μg/mL ampicillin, 1 mM $MgCl_2$) and incubated at 37 °C until an OD600 nm of 0.8-1.2 was reached. Protein expression was then induced with 0.4 mM isopropyl-β-d-thiogalactoside (IPTG), and cultures were incubated overnight at 18˚C. Cells were harvested by centrifugation (8600 rpm, 20 minutes, 4 ˚C) and resuspended in lysis buffer (50 mM Hepes, pH 8.0, 300 mM NaCl, 12.5 mM imidazole, 5 mM $MgCl_2$, 2 mM βME) containing cOmplete™ EDTA-free Protease Inhibitor Cocktail tablets (1 tablet/ 100 ml lysate) and benzonase (5 μl/ 100 ml). After lysis using a C3 Emulsiflex-C3 homogenizer (Avestin), the cell lysate was cleared by centrifugation (20000 rpm, 30 minutes, 4 ˚C) and purified using Ni^{2+}-affinity chromatography (Ni-NTA superflow cartridges, Qiagen). Typically two 5 mL columns were used for a 2 L culture in which the lysate was loaded on the column, washed with 10 CV wash buffer (50 mM Hepes, pH 8.0, 300 mM NaCl, 25 mM imidazole, 2 mM βME), and eluted with several fractions (2-4 CV) of elution buffer (50 mM Hepes, pH 8.0, 300 mM NaCl, 250 mM imidazole, 2 mM βME). Fractions containing the 14-3-3 protein were combined and dialyzed into 25 mM HEPES pH 8.0, 200 mM NaCl, 10 mM $MgCl_2$, 2 mM βME. In addition, 1 mg TEV was added for each 100 mg purified protein to remove the purification tag. The cleaved sample was then again loaded on a 10 mL Ni-NTA column to separate the cleaved product from the expression tag and residual uncleaved protein. The flowthrough was loaded on a Superdex 75 pg 16/60 size exclusion column (GE Life Sciences) using 25 mM HEPES, 100 mM NaCl, 10 mM $MgCl_2$, 500 μM TCEP (adjusted to pH=8.0) as running buffer. Fractions containing the 14-3-3 protein were pooled and concentrated to ~60 mg/mL, analyzed Q-Tof LC/MS, and aliquots were flash-frozen for storage at −80 °C.

Fluorescence anisotropy assay

14-3-3γ was titrated in a 2-fold dilution series (starting at 400 μM 14-3-3γ) to 100 or 10 nM of fluorescein labeled peptide (most often ERRγ-pS179-9mer) in FA buffer (10 mM HEPES pH 8.0, 150 mM NaCl, 50 μM TCEP, 0.1% (v/v) Tween20, 0.1% (w/v) BSA). This is done in absence (DMSO control) or presence of 100 μM compound (20-100 mM stock in DMSO); each well contained a final concentration of 0.5% DMSO. Dilution series were made in a polystyrene (non-binding) low-volume Corning Black Round Bottom 384-well plates (Corning 4514 or 4511). Measurements were performed directly after plate preparation, using a Tecan Infinite F500 plate reader at room temperature (l_{ex}: 485 ± 20 nm; l_{em}: 535 ± 25 nm; mirror: Dichroic 510; flashes: 20; integration time: 50 ms; settle time: 0 ms; gain: 60; and Z-position: calculated from well). Wells containing only fluorescein labelled peptide were used to set as G-factor at 35 mP. All data were analyzed using GraphPad Prism (7.00) for Windows and fitted using a four-parameter logistic model (4PL) to determine apparent binding affinities (K_D^{app}). All results are

based on three independent experiments from which the average and standard deviations for each K_D were determined using excel.
Within each kinetic experiment, the plate is incubated over time at room temperature and remeasured after x amount of time to obtain results over time.
Within compound titrations a similar assay was performed however here compound was titrated in a 2-fold dilution series (starting from 1 mM) to 1 μM 14-3-3γ and 100 nM fluorescein labelled ERRγ peptide (50 μM BME in buffer instead of 50 μM TCEP). Each well contained a final concentration of 1% DMSO. Plate preparations and measurements are similar to 14-3-3γ titrations.

Differential Scanning Fluorimetry (DSF)
Different Scanning Fluorimetry (DSF) assays were performed using 40 μL samples containing 5 μM 14-3-3γ, 50 μM ERRγ pS179 9-mer peptide, 200 μM compound (or 1% DMSO) and 5x ProteoOrange (Lumiprobe, 5000x stock in DMSO) in 10 mM Hepes, 150 mM NaCl, 50 μM TCEP (pH 8.0). The samples were heated from 35 °C to 79 °C at a rate of 0.3 °C per 15 s in a CFX96 Touch Real-Time PCR Detection System (Bio-Rad). Fluorescence intensity was determined using excitation 470/40 nm and emission 570/20 nm filters. Based on these melting curves, the negative derivative melting curve is obtained from which the melting temperature T_m was determined. ΔT_m values represent the differences in melting temperature relative to the DMSO control. All described melting temperatures are based on three independent experiments from which the average and standard deviations were determined using excel.

Wash-out experiment
Biotinylated 14-3-3γ (5 μM) was preincubated with fluorescein-labelled ERRγ 9-mer peptide (200 nM) in presence of DMSO (negative control) or 100 μM compounds **1**, **3** or **5** in buffer ((10 mM HEPES pH 8.0, 150 mM NaCl, 50 μM TCEP, 0.1% (v/v) Tween20, 0.1% (w/v) BSA) to a final volume of 100 μL. Similar samples were prepared in absence of biotinylated 14-3-3γ to function as a control experiment and to normalize the data for aspecific binding of the FAM-ERRγ peptide to the magnetic beads. Samples were incubated overnight at 4°C. For each sample 120 μL of 4 mg/mL (480 μg) streptavidin-functionalized magnetic beads were washed 5 times with 1 mL buffer. From each sample, 30 μL sample was added to the magnetic beads which were than incubated for 1 hour at 4°C to capture the biotinylated 14-3-3γ on the streptavidin beads. Sample was than removed and stored for later fluorescence measurements. Samples were than washed with 30 μL buffer with an incubation time of 20 minutes for each wash step. Wash buffer was than removed and stored for fluorescence measurements as well. Washing steps were repeated three more times. For each sample from before capturing, after capturing and each wash step, 2x 10 μL was placed into an polystyrene non-binding low-volume Corning Black Round Bottom 384-well plates (Corning 4514). Fluorescence was measured using the Tecan SPARK plate reader at room temperature with an excitation wavelength of 485 nm and an emission wavelength of 535 nm. Furthermore, fluorescence anisotropy of each sample was measured using the Tecan Infinite F500 plate reader at room temperature (l_{ex}: 485 ± 20 nm; l_{em}: 535 ± 25 nm; mirror: Dichroic 510; flashes: 20; integration time: 50 ms; settle time: 0 ms; gain: 50; and Z-position: calculated from well) to determine whether all 14-3-3γ was captured by the magnetic beads (should results in low anisotropy values in samples after capturing in comparison to samples before capturing. All described values are based on three independent experiments from which the average and standard deviations were determined using excel.

Mass Spectrometry (MS) Assays
Initial crosslinking of 14-3-3γ and ERRγ by compound **5** and **10** using *in situ* quenching: A reaction mixture containing 14-3-3γ (5 μM), N-acetylated ERRγ peptide (25 μM), and compound (50 μM) in buffer (10 mM HEPES, 150 mM NaCl, 50 μM TCEP, 5 mM $NaCNBH_3$, 2.5 v/v% DMSO, pH 8.0) was incubated at room temperature. $NaCNBH_3$ was added to quench the acid-label imine bond formed between 14-3-3 and the compounds. After 5, 20, 40, 80, 240, and 420 min, a 10 μL sample was taken from the mixture, and snap frozen in liquid nitrogen. After all samples had been collected, the samples were thawed and diluted into 490 μL ultrapure water (with 0.1% formic acid (FA)).

Crowded environment experiment with 14-3-3, nine peptides and compound **5** using afterwards quenching: A reaction mixture containing 14-3-3γ (5 μM), compound **5** (50 μM) and either N-acetylated ERRγ (10 μM) or nine N-acetylated peptides (10 μM ERRγ, 10 μM ERα, 10 μM Pin1, 10 μM USP8, 10 μM p65, 10 μM RND3, 10 μM PKR, 10 μM SOS1, 10 μM BRAF) in buffer (10 mM HEPES, 150 mM NaCl, 50 μM TCEP, 2.5 v/v% DMSO, pH 8.0) was incubated at room temperature. After 5, 20, 40, 80, 240, and 420 min, a 10 μL sample was taken from the mixture,

and snap frozen in liquid nitrogen. After all samples had been collected, the samples were thawed and 5 µL of a 10 mM $NaCNBH_3$ solution in buffer was added to reduce the imine bond to a secondary amine. After 15 min of incubation, the mixtures were each diluted into 485 µL ultrapure water with 0.1% formic acid (FA) to quench the imine reduction.

All resulting 500 µL samples were each transferred to an Amicon Ultra 0.5 mL 10K centrifugal filter and concentrated (5 min, 13400 rpm). The concentrates were each diluted to 500 µL and concentrated again, until five desalting steps were performed. The final concentrates (~100 µL) were transferred to a 200 µL LC-MS vial, snap frozen in liquid nitrogen, and stored at -30 °C prior to analysis. UPLC-QToF-MS analysis was performed on a Waters (Milford, MA, USA) Acquity I-Class UPLC system coupled to a Waters Xevo G2-XS quadrupole time-of-flight (QToF) mass spectrometer. The devices were controlled by MassLynx Software (version 4.2, Waters, MA, USA). Full scan in positive electrospray ionization (ESI+) mode was used as MS acquisition mode with an acquisition range from 150 – 2000 m/z. A 3 µm, 100 x 2.0 mm Polaris 3 C18-A column (Agilent, Middelburg, the Netherlands) was placed inside a column oven at 40°C and used for chromatographic separation. Flowrate was set at 0.3 mL/min, and a gradient of water containing 0.1% (v/v) formic acid (A) and acetonitrile containing 0.1% (v/v) formic acid (B) was set as follows (all displayed as % v/v): 0.0-7.5 min (37% to 41% B), 7.5-8.0 min (41% B), 8.0-8.1 min (41% to 37% B), 8.1-10.0 min (37% B). Sample injection volume 2 µL. Mass Spectrometry settings were set as follows: capillary voltage: 0.80 kV, cone voltage: 40 V, source offset: 80 V, source temperature: 120°C, desolvation temperature: 450°C, cone gas: 10 L/h desolvation gas: 1000 L/h.
Data was analyzed using MassLynx software. Chromatograms were background subtracted (polynomial order 1, below curve 40%, tolerance 0.010, flatten edges). To perform mass analysis of the individual peaks were performed by selecting a specific retention time interval for all peaks which are given with each supplementary figure (Figure S8, S10, S20, S21). These peaks were deconvoluted while subtracting the background signal (defined as 1.50- 2.00 min interval). After visual inspection of the m/z spectrum, the spectrum was zoomed (900 - 1040 m/z) from which the mass spectrum was determined using MaxEnt1 (mass ranges 30,000 – 34,000; resolution 0.10 Da/channel, Simulated Isotope Pattern with Spectrometer Blur width 0.32-0.38 Da, minimum intensity ratios left 33%, right 33%, iterate to converge). Mass spectra were centered and errors of the decovolution process were determined. Proteins or protein complex were than quantified by calculating the peak area of each determined mass. Percentages of complex formation were calculated via mass $area_{14\text{-}3\text{-}3\gamma/ERR\gamma/compound}$ / (mass $area_{14\text{-}3\text{-}3\gamma/ERR\gamma/compound}$ + mass $intensity_{14\text{-}3\text{-}3\gamma}$) * 100%.

Modelling of crowded environment:
To determine the 14-3-3 occupancy by each phosphopeptide when preparing a mixture of 14-3-3 with nine different 14-3-3-binding phosphopeptides, we have used a multi-component thermodynamic equilibrium model as described by Geertjens et al. (2021).[1] This general platform generates a customized model to describe multi-component equilibrium systems when given a system description. In our case the following system description was given:

A + B = AB; K_D^1
A + C = AC; K_D^2
A + D = AD; K_D^3
A + E = AE; K_D^4
A + F = AF; K_D^5
A + G = AG; K_D^6
A + H = AH; K_D^7
A + I = AI; K_D^8
A + J = AJ; K_D^9

Based on this system description the framework is able to determine mass balance and equilibrium equations and creates a custom model which allows the prediction of unknown parameters in this system, such as the concentration of specific species.
We provided the model the concentrations of each species as used in the MS studies; A = 14-3-3γ = 5 µM; B-J = phosphopeptides = 10 µM each. Furthermore we provided the model with all K_D values as obtained from FA assays (see Fig S22) and let it calculate the presence of all 14-3-3/phosphopeptide complexes: AB, AC, AD, AE… etc. Based on these concentrations, we calculated the percentage of 14-3-3 occupied by each peptide.
In order to determine the effect of stabilizer 5 to the 14-3-3 occupancy, we have increased affinity (K_D1) of the ERRγ (B) peptide to 14-3-3 (A) until 35% of the 14-3-3 was occupied by ERRγ (35% of all A in AB).

Crystallography

To obtain co-crystal structures of 14-3-3, ERRγ phosphopeptide (or RND3/PKR) and the compounds, a complexation mixture is made containing a final 14-3-3σΔc (truncated after T231 to reduce flexibility) concentration of 10 mg/mL, 1:2:2 or 1:2:5 protein-peptide-compound molar ratio in 25 mM HEPES pH 7.5, 100 mM $MgCl_2$, and 100 μM TCEP. After overnight complexation at 4°C, sitting drop crystallization wells were set-up using 250 nL complex mixture and 250 nL precipitation buffer (95 mM HEPES pH 7.1-7.9, 0.19 M $CaCl_2$, 5% glycerol and 25-29% PEG400). Crystals grew within 10 - 14 days at 4 °C. Suitable crystals were fished and flash-cooled in liquid nitrogen. X-ray diffraction data were collected at the p11 beamline of PETRA III facility at DESY (Hamburg, Germany) which is equipped with a DECTRIS EIGER X 16M detector. Typical settings were 1440 image, 0.25°/image, 100% transmission and 0.1 s exposure time.

Data was processed using the CCP4i2 suite (version 7.1.10).[2] DIALS[3] was used to index and integrate the data after which scaling was done using AIMLESS.[4,5] The data was phased with MolRep[6], using protein data bank (PDB) entry 6Y1D as a template. A three dimensional structure of each compound was generated using AceDRG[7], which was thereafter build in based on visual inspection Fo-Fc and 2Fo-Fc electron density map. Sequential model building (based on visual inspection Fo-Fc and 2Fo-Fc electron density map) and refinement were performed with COOT and REFMAC, respectively.[8-10] Finally, alternating cycles of model improvement (based on isotropic b-factors and standard set of stereo-chemical restraints: covalent bonds, angels, dihedrals, planarities, chiralities, non-bonded) and refinements were performed using coot and phenix.refine from the Phenix software suite (version 1.20.1).[11,12] Pymol (version 2.2.3)[13] was used to make the figures and the structures were deposited in the protein data bank (PDB). See table S3 for crystal statistics.

HEK293 cell lysate extraction and FA assays

HEK293T cells were cultured in DMEM (Gibco) with 10% FBS and penicillin/ streptomycin and incubated at 37 °C in a humidified 5% CO2 atmosphere. Cells were incubated for 72 hours and then harvested by scraping. The whole-cell extract was prepared by resuspending the cells in 500 µl of ice-cold Pierce™ IP Lysis Buffer (Thermo Fisher Scientific) supplemented with EDTA-free protease, phosphatase inhibitors cocktails (Sigma-Aldrich), and Benzonase (1:200, Millipore). The lysate was incubated for 30 minutes at 4°C before centrifugation at 13,400 rpm for 15 minutes at 4°C. The supernatant containing whole protein extract was collected and quantified with DC-Protein assay (Bio-Rad).

For FA - cell lysate experiments, HEK293 cell lysate or cell lysis buffer were titrated in a 2-fold dilution series (starting from 20 mg/mL) to a preformed complex of 5 μM 14-3-3γ and 100 nM fluorescein labelled ERRγ peptide in FA buffer (10 mM HEPES pH 8.0, 150 mM NaCl, 50 μM TCEP, 0.1% (v/v) Tween20, 0.1% (w/v) BSA). At these concentrations, ~50% of the ERRg peptide is bound to 14-3-3γ.
Alternatively, compound 5 (starting at 1 mM) was titrated in a 2-fold dilution series to preformed complex of 5 μM 14-3-3γ and 100 nM fluorescein labelled ERRγ peptide in FA buffer (10 mM HEPES pH 8.0, 150 mM NaCl, 50 μM TCEP, 0.1% (v/v) Tween20, 0.1% (w/v) BSA) with varying concentrations of HEK 293 cell lysate (0.03-2 mg/mL). Results presented are an average (with standard deviation) of three independent experiments.
Dilution series were made in a polystyrene (non-binding) low-volume Corning Black Round Bottom 384-well plates (Corning 4514 or 4511). Measurements were performed directly after plate preparation, using a Tecan Infinite F500 plate reader at room temperature (l_{ex}: 485 ± 20 nm; l_{em}: 535 ± 25 nm; mirror: Dichroic 510; flashes: 20; integration time: 50 ms; settle time: 0 ms; gain: 60; and Z-position: calculated from well). Wells containing only fluorescein labelled peptide were used to set as G-factor at 35 mP. All data were analyzed using GraphPad Prism (7.00) for Windows and fitted using a four-parameter logistic model (4PL) to determine apparent binding affinities (K_D^{app}).

Experimental Section Chemistry

All reactions were prepared using AR or HPLC grade solvents without further purification. All reagents were purchase from Fluorochem, ABCR, Ak Scientific or Sigma-Aldrich and were used without further purification unless stated. Solvents were removed *in vacuo* using a Buchi rotary evaporator and a diaphragm pump. DMF and CH_2Cl_2 were dried and purified by means of a MBRAUN Solvent Purification System (MB-SPS-800). All other solvents used were of chromatography or analytical grade and supplied by Biosolve or Sigma-Aldrich. TLC was carried out on aluminum-backed silica (Merck silica gel 60 F254) plates supplied by Merck. Visualization of the plates was achieved using an ultraviolet lamp (λmax = 254 nm), 2,4-DNP, $KMnO_4$, anisaldehyde, bromine or ninhydrin. Column chromatography was either manually using silica gel (60-63 µm particle size), automated Grace Reveleris X2 or Biotage Isolera chromatograph with prepacked silica columns supplied by Buchi/Grace (40 µm particle size). LC–MS analysis was carried out with a system comprising a Phenomex kinetex® 2.6 µm EVO C18 50 x 2.1 mm column using ultrapure water with 0.1% formic acid (FA) and acetonitrile with 0.1% FA, in general with using a gradient of 5–100% MeCN in water (+ 0.1% HCOOH) over 10 min, connected to a Thermo Fisher LCQ Fleet Ion Trap Mass Spectrometer. The purity of the samples was assessed using a PDA and MS. Unless otherwise stated all final compounds were ≥95% pure as judged by HPLC. GCMS analysis was performed on a Phenomenex Zebron ZB-5MS 30 m × 0.25 mm × 0.25 mm column with a gradient of 80 °C for 1 min to 300 °C for 1 min with a rate of 30 °C/min in helium gas connected to a GCMS-QP2010 Plus Quadrupole Mass Spectrometer. High resolution mass spectra (HRMS) were recorded using a Waters ACQUITY UPLC I-Class LC system coupled to a Xevo G2 Quadrupole Time of Flight (Q-tof) mass spectrometer equipped with a Phenomex kinetex® 2.6 µm EVO C18 100 x 2.1 mm column. Proton (1H) and carbon (13C) NMR spectral data were collected on a 400 MHz Bruker Cryomagnet or 400 MHz Varian Gemini. Chemical shifts (δ) are quoted in parts per million (ppm) and referenced to the residual solvent peak. Coupling constants (*J*) are quoted in Hertz (Hz) and splitting patterns reported in an abbreviated manner: app. (apparent), s (singlet), d (doublet), t (triplet), q (quartet), and m (multiplet). Assignments were made with the aid of 2D COSY, HMQC, and HMBC experiments.

Synthetic procedure and characterization

General procedure

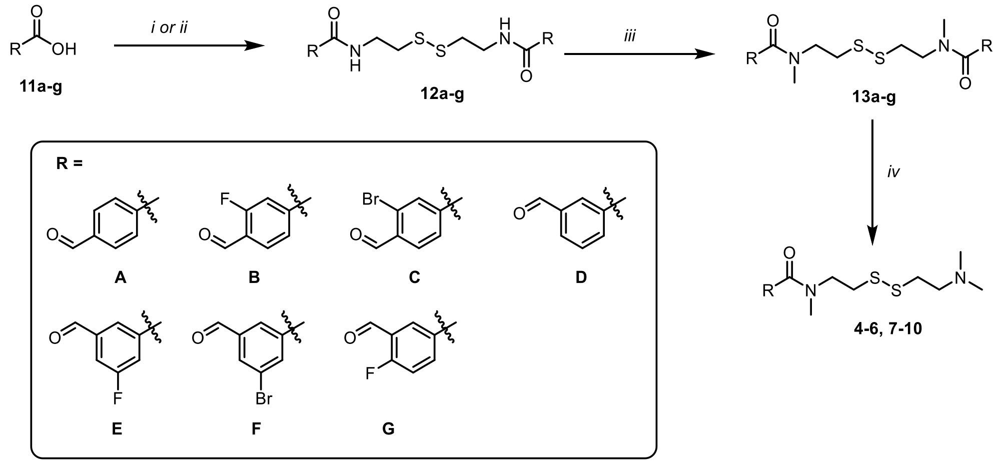

Scheme S1. Synthesis of a *N*-methylbenzamide analogs. Reagents and conditions: *(i)* cystamine dihydrochloride, HOBt, EDC, TEA, H_2O, DMF, rt, overnight (O/N); (*ii*) cystamine dihydrochloride, HBTU, DIPEA, DMF, rt, O/N. (*iii*) NaH, MeI, DMF, 0 °C – rt, 45 min,; (*iv*) 2,2'-Bis(dimethylamino)diethyl disulfide dihydrochloride, TECP, DIPEA, DMF/H_2O (1:1), rt, O/N,.

Amide coupling

Conditions i (compounds 12a, 12b and 12d).

To a 20 mL glass vial was added the corresponding carboxylic acid (2 mmol) and a solution of cystamine dihydrochloride (1 mmol), 1-hydroxybenzotriazole (HOBt, 0.2 mmol), triethylamine (TEA, 2 mmol) in (1:6) H_2O/DMF (7 mL). The reaction mixture was then stirred until homogenous and a solution of *N*-ethyl-*N*'-(3-dimethylaminopropyl)carbodiimide HCl (EDC, 4.4 mmol) in H_2O (0.680 mL) was added. The reaction was then

stirred overnight at room temperature. The resulting reaction mixture was then diluted with H_2O (200 mL). The reaction mixture was then either filtered under vacuum and the precipitate collected, or the product was extracted with 2 x 100 mL of EtOAc, washed with brine (200 mL). The organic layer was then separated, dried over Mg_2SO_4 and concentrated under vacuum to afford the desired product.

Conditions ii (compounds 12c, 12e, 12f and 12g).

To a 20 mL glass vial was added a solution of the corresponding carboxylic acid (2 mmol), *N,N*-Diisopropylethylamine (DIPEA, 6 mmol), *N,N,N',N'*-tetramethyl-*O*-(1*H*-benzotriazol-1-yl)uronium hexafluorophosphate (HBTU, 2 mmol), in DMF (3 mL). The reaction mixture was then stirred for 10 min, before addition of a solution of cystamine dihydrochloride (1 mmol) in DMF (2 mL). The reaction mixture was then stirred overnight at room temperature. The resulting reaction mixture was then diluted in 1 M aqueous hydrochloric acid (~100 mL). The resulting suspension was then sonicated for approx. 2 min, filtered and dried under vacuum to afford the product.

Methylation (Scheme 1iii)

To a 20 mL glass vial was added a solution of disulfide **12** (0.2 mmol) in anhydrous DMF (2 mL). The stirred solution was cooled to 0°C and a 60% dispersion of Sodium hydride in mineral oil (0.9 mmol) was added portion-wise over 10 min. The reaction mixture was then stirred for an additional 10 min before the addition of iodomethane (0.9 mmol). The reaction was warmed to room temperature and stirred for 45 min. The resulting reaction mixture was then diluted in H_2O (8 mL) and the products were extracted with ethyl acetate (2x 4 mL). The organic layer was washed with brine (4 mL) and dried over $NaSO_4$. The solvent was evaporated under reduced pressure to afford the product. The isolated oil was used in the next reaction as is.

Disulfide exchange (Scheme 1iv)

To a 20 mL vial was added the crude methylated disulfide dimer **13** (0.2 mmol) and DIPEA (2 mmol) in anhydrous DMF (2 mL). To the reaction mixture was then added a solution bis(2-dimethylaminoethyl) disulfide HCl (1.0 mmol) and tris(2-carboxyethyl)phosphine HCl (TCEP, 0.04) in H_2O (2 mL). The reaction mixture was then stirred overnight at room temperature. The resulting crude mixture was concentrated under vacuum and the crude material was purified using reverse-phase column chromatography (C18, H_2O:ACN) to afford the desired product.

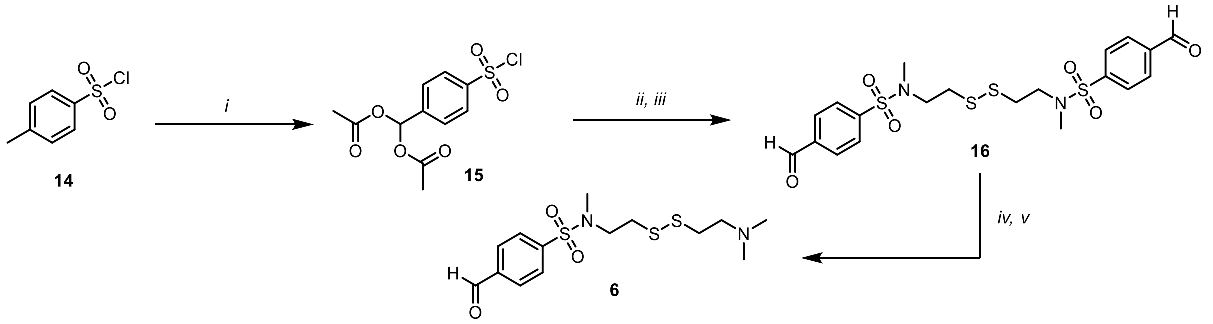

Scheme 2. Synthetic route to access *N*-methylbenzenesulfonamide. Reagents and conditions: (*i*) CrO_3, AcOH, H_2SO_4, 0 °C, 40 min, 39%; (*ii*) cystamine dihydrochloride, TEA, anhydrous DCM, Ar, rt, O/N, 38%; (*iii)* 1 M $HCl_{(aq)}$ DMF (1:1), rt, followed by TLC (EtOAc/DCM), 64%; (*iv*) NaH, MeI, DMF, 0 °C – rt, 45 min, quant. (*v*) 2,2'-Bis(dimethylamino)diethyl disulfide dihydrochloride, TECP, DIPEA, DMF/H_2O (1:1), rt, O/N, 74%.

Methylene diacetate (15, Scheme 2i**)**

A 1 L RB flask was charged with a solution of 4-methylbenzenesulfonyl chloride (236 mmol), acetic anhydride (3969 mmol) in acetic acid (375 mL) and cooled to 0 °C. To the solution was then added concentrated sulfuric acid (571 mmol), followed by portion-wise addition of chromium(VI) oxide (660 mmol). The mixture was stirred at room temperature for 30 minutes, poured into ice/water and the solid was collected by filtration. The solid was dissolved in dichloromethane, dried over magnesium sulfate and concentrated under reduced pressure. The residue was crystallized from a mixture of acetone and hexane to afford the desired compound in 39% yield. ^{1}H NMR ($CDCl_3$): δ 2.15 (s, 6H), 7.75 (s, 1H), 7.80 (d, *J= 8.6 Hz*, 2H), 8.10 (d, *J= 8.6 Hz*, 2H). ^{13}C NMR (100 MHz, CDCl3) δ 168.2, 146.0, 142.3, 131.6, 129.5, 126.1, 123.7, 88.1, 20.7.

Sulfonyl amide coupling (Scheme 2ii)

A 50 ml RB flask was charged with cystamine 2HCl (0.6 mmol) and (4-(chlorosulfonyl)phenyl)methylene diacetate (1.2 mmol) followed by addition of DCM (10 mL). The flask was put under an argon atmosphere and TEA (1.8 mmol) was subsequently added. The mixture was stirred overnight at rt. The reaction mixture was then diluted with 1M solution $NaHCO_3$ (20 mL). The organic layer was extracted with DCM (15 mL). The organic layer was washed with brine (10 mL), dried over $NaSO_4$ and the solvent evaporated to afford a yellow oil. The crude residue was purified by column chromatography (EtOAc/DCM) providing the desired product as a white crystalline solid (159.6 mg, 37.8%).

Aldehyde deprotection (16, Scheme 2iii**)**
The bis-disulfide was redissolved in DMF (5 mL) and 1M aqueous HCl solution (5 mL) was added. The mixture was allowed to stir at rt until TLC analysis showed full consumption of the starting material (EtOAc/DCM). The solvent was removed in vacuo and carried through without further purification.

Methylation (Scheme 2iv)

To a 20 mL glass vial was added a solution of disulfide **16** (0.2 mmol) in anhydrous DMF (2 mL). The stirred solution was cooled to 0°C and a 60% dispersion of Sodium hydride in mineral oil (0.9 mmol) was added portion-wise over 10 min. The reaction mixture was then stirred for an additional 10 min before the addition of iodomethane (0.9 mmol). The reaction was warmed to room temperature and stirred for 45 min. The resulting reaction mixture was then diluted in H_2O (8 mL) and the products were extracted with ethyl acetate (2x 4 mL). The organic layer was washed with brine (4 mL) and dried over $NaSO_4$. The solvent was evaporated under reduced pressure to afford the product. The isolated oil was used in the next reaction as is.

Disulfide exchange (Scheme 1iv)

To a 20 mL vial was added the crude methylated disulfide dimer (0.2 mmol) and DIPEA (2 mmol) in anhydrous DMF (2 mL). To the reaction mixture was then added a solution bis(2-dimethylaminoethyl) disulfide HCl (1.0 mmol) and tris(2-carboxyethyl)phosphine HCl (TCEP, 0.04) in H_2O (2 mL). The reaction mixture was then stirred overnight at room temperature. The resulting crude mixture was concentrated under vacuum and the crude material was purified using reverse-phase column chromatography (C18, H_2O:ACN) to afford the desired product.

N-(2-(dimethylamino)ethyl)-3-fluoro-4-formyl-benzamide (**3**)
Compound **3** was a yellow oil (107 mg, 41%). HRMS (ESI) calcd for $C_{13}H_{18}FN_2O_2$ (M+H), 239.1196; found 239.1191. ^{1}H NMR (400 MHz, DMSO-d6) δ 10.26 (s, 1H), 9.67 (s, 1H), 9.06 – 8.87 (m, 1H), 8.03 – 7.89 (m, 1H), 7.89 – 7.69 (m, 2H), 3.63 (q, *J = 5.9 Hz*, 2H), 3.33 – 3.21 (m, 2H), 2.85 (s, 7H). ^{13}C NMR (100 MHz, DMSO) δ 188.1, 188.1, 165.3, 165.3, 164.6, 162.1, 141.6, 141.5, 130.1, 130.1, 126.0, 126.0, 116.2, 116.0, 56.2, 42.9, 35.2.

***N*-(2-((2-(dimethylamino)ethyl)disulfaneyl)ethyl)-4-formyl-*N*-methylbenzamide (4)**
Compound **4** was a yellow oil (47.7 mg, 24%). HRMS (ESI) calcd for $C_{15}H_{23}N_2O_2S_2$ (M+H), 327.1201; found 327.1208.^{1}H NMR (400 MHz, Chloroform-*d*) δ 10.02 (s, 1H), 7.91 (d, *J* = 7.7 Hz, 2H), 7.55 (d, *J* = 6.6 Hz, 2H), 3.89 – 3.77 (m, 1H), 3.57 – 3.49 (m, 1H), 3.12 – 2.96 (m, 4H), 2.90 – 2.81 (m, 1H), 2.79 – 2.69 (m, 1H), 2.66 – 2.57 (m, 2H), 2.51 – 2.42 (m, 1H), 2.23 (d, *J* = 25.5 Hz, 6H). ^{13}C NMR (100 MHz, $CDCl_3$) δ 191.5, 170.2 (170.8, minor), 142.0, 136.8 (136.7, minor), 129.9 (2C, 129.9, minor), 127.6 (2C), 58.6 (58.4, minor), 47.2 (50.4, minor), 45.3 (2C), 38.7 (33.01, minor), 36.9 (36.7, minor), 35.6 (35.9, minor).

***N*-(2-((2-(dimethylamino)ethyl)disulfaneyl)ethyl)-3-fluoro-4-formyl-*N*-methylbenzamide** (**5**)
Compound **5** was a colorless oil (88.6 mg, 25%). HRMS (ESI) calcd for $C_{15}H_{22}N_2O_2FS_2$ (M+H), 345.1107; found 345.1100. ^{1}H NMR (400 MHz, Chloroform-*d*) δ 10.38 (s, 1H), 7.93 (t, *J* = 7.3 Hz, 1H), 7.29 (dd, J = 23.2, 8.3, 7.2 Hz, 2H), 3.88 – 3.80 (m, 1H), 3.59 – 3.53 (m, 1H), 3.14 – 2.99 (m, 4H), 2.91 – 2.84 (m, 1H), 2.83 – 2.76 (m, 1H), 2.72 – 2.61 (m, 2H), 2.56 – 2.48 (m, 1H), 2.31 – 2.21 (m, 6H). ^{13}C NMR (100 MHz, $CDCl_3$) δ 186.6 (d, *J* = 6.4 Hz), 169.0, 165.8, 163.2, 144.3 (d, *J* = 8.3 Hz), 129.3 (d, *J* = 7.0 Hz), 123.3 (d, *J* = 3.8 Hz), 115.6 (d, *J* = 21.8 Hz), 58.7 (58.5, minor), 47.4 (50.3, minor), 45.4 (2C), 38.8 (33.1, minor) 37.0 (36.7, minor), 35.6 (36.00, minor).

***N*-(2-((2-(dimethylamino)ethyl)disulfaneyl)ethyl)-3-bromo-4-formyl-*N*-methylbenzamide** (**6**)
Compound **6** was a yellow oil (50 mg, 62%). HRMS (ESI) calcd for $C_{15}H_{22}Br7N_2O_2S_2$ (M+H; Br^{79}, Br^{81}), 405.0306, 407.0285; found 405.0293, 407.0272. ^{1}H NMR (399 MHz, Acetone-*d_6*) δ 10.34 (s, 1H), 7.94 (d, *J* = 7.9 Hz, 1H), 7.79 (d, *J* = 1.5 Hz, 1H), 7.60 (d, *J* = 7.7 Hz, 1H), 3.91 – 3.79 (m, 1H) , 3.64 (s, 1H), 3.59 – 3.51 (m, 1H), 3.42 (s, 1H), 3.33 – 3.22 (m, 2H), 3.18 – 2.91 (m, 11H). ^{13}C NMR (100 MHz, Acetone) δ 190.6 (190.07, minor), 168.3 (apparent doublet), 143.7 (143.9, minor), 133.9 (133.8, minor), 132.2 (132.3, minor), 130.0 (apparent doublet), 126.6

(126.8, minor), 126.4, 126.0, 125.9, 56.3 (56.0), 49.5, 46.4, 42.1, 37.1, 34.95 (35.03, minor), 32.0, 31.3 (31.4, minor), 30.5.

***N*-(2-((2-(dimethylamino)ethyl)disulfaneyl)ethyl)-4-formyl-*N*-methylbenzenesulfonamide** (**7**)
Compound **7** was a product yellow oil (32.63 mg, overall yield: 7.4%). HRMS (ESI) calcd for $C_{14}H_{23}N_2O_3S_3$ (M+H), 363.0871; found 363.0866. ^{1}H NMR (400 MHz, Chloroform-*d*) δ 10.11 (s, 1H), 8.01 (dd, *J* = 28.8, 7.8 *Hz*, 4H), 3.42 – 3.34 (m, 2H), 2.91 – 2.79 (m, 7H), 2.66 – 2.58 (m, 2H), 2.28 (s, 6H) ^{13}C NMR (100 MHz, $CDCl_3$) δ 190.8, 143.0, 138.9, 130.3 (2C), 128.0 (2C), 58.5, 49.8, 45.2 (2C), 36.8, 36.4, 35.7.

***N*-(2-((2-(dimethylamino)ethyl)disulfaneyl)ethyl)-3-formyl-*N*-methylbenzamide** (**8**)
Compound **8** was a yellow oil (59.05 mg, 26.5%). HRMS (ESI) calcd for $C_{15}H_{23}N_2O_2S_2$ (M+H), 327.1201; found 327.1197. ^{1}H NMR (400 MHz, Chloroform-*d*) δ 9.99 (s, 1H), 7.88 (d, *J* = 6.1 Hz, 2H), 7.65 (d, *J* = 7.7 Hz, 1H), 7.55 (t, *J* = 7.7 Hz, 1H), 3.85 – 3.75 (brm, 1H), 3.62 – 3.48 (brm, 1H), 3.11 – 2.93 (m, 4H), 2.88 – 2.69 (brm, 2H), 2.59 (brs, 2H), 2.44 (brs, 1H), 2.26 – 2.11 (m, 6H). ^{13}C NMR (100 MHz, $CDCl_3$) δ 191.4, 170.1, (170.7 minor), 137.3, 136.3, 132.8, 130.6 (130.5, minor), 129.3, 128.1, 58.6 (58.4, minor), 47.4 (50.5, minor), 45.3 (2C), 38.8 (33.1 minor), 36.9 (36.6, minor), 35.50 (36.0, minor).

***N*-(2-((2-(dimethylamino)ethyl)disulfaneyl)ethyl)-4-fluoro-3-formyl-*N*-methylbenzamide** (**9**)
Compound **9** was a yellow oil (50.5, 42%). HRMS (ESI) calcd for $C_{15}H_{21}FN_2O_2S_2$ (M+H), 345.1107; found 345.1103.^{1}H NMR (399 MHz, DMSO-d_6) δ 10.23 (s, 1H), 8.14 (s, 1H), 7.86 (dd, *J* = 6.7, 2.3 Hz, 1H), 7.80 (ddd, *J* = 8.6, 5.0, 2.3 Hz, 1H), 7.49 (dd, *J* = 10.5, 8.5 Hz, 1H), 3.74 (s, 2H), 3.51 (s, 2H), 3.08 – 2.86 (m, 4H), 2.83 – 2.66 (m, 1H), 2.33 (s, 4H), 2.25 (s, 3H). ^{13}C NMR (100 MHz, Chloroform-*d*) δ 186.3 (d, *J* = 6.2 Hz), 166.2 (minor, 163.6), 135.5, 133.0, 127.5, 123.7 (d, *J* = 8.8 Hz), 117.2 (d, *J* = 25.3 Hz), 57.65 (minor, 57.5), 47.3, 43.9 (minor, 44.0), 38.7, 35.4, 33.9.

3-bromo-*N*-(2-((2-(dimethylamino)ethyl)disulfaneyl)ethyl)-5-formyl-*N*-methylbenzamide (**10**)
Compound **10** was a yellow oil (52.4 mg, 44%). HRMS (ESI) calcd for $C_{15}H_{22}Br7N_2O_2S_2$ (M+H; Br^{79}, Br^{81}), 405.0306, 407.0285; found 405.0293, 407.0273. ^{1}H NMR (399 MHz, Chloroform-*d*) δ 9.98 (s, 1H), 8.06 (s, 1H), 7.84 (t, *J* = 1.5 Hz, 1H), 7.81 (t, *J* = 1.7 Hz, 1H), 3.94 – 3.78 (m, 2H), 3.59 (s, 1H), 3.47 – 3.34 (m, 2H), 3.29 (s, 0H), 3.16 – 2.75 (m, 10H).^{13}C NMR (100 MHz, $CDCl_3$) δ 189.9 (190.2, minor), 162.0 (apparent quartet), 137.9 (139.2, 138.8, minor), 135.7 (135.7, minor), 133.5 (133.3, minor), 126.5, 123.6, 57.2 (57.1, minor), 47.1 (49.9, minor), 43.4, 38.4, 35.2 (38.4, minor), 31.3.

Compound 3 (PC714)

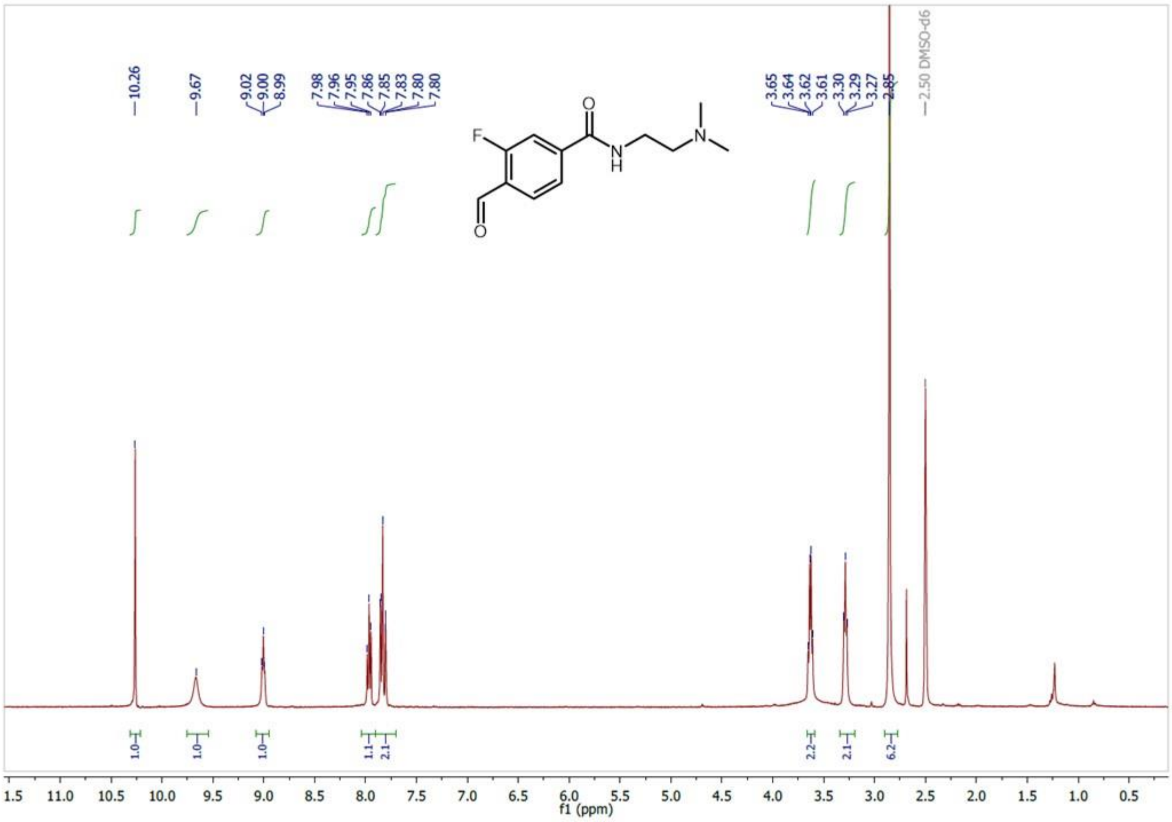

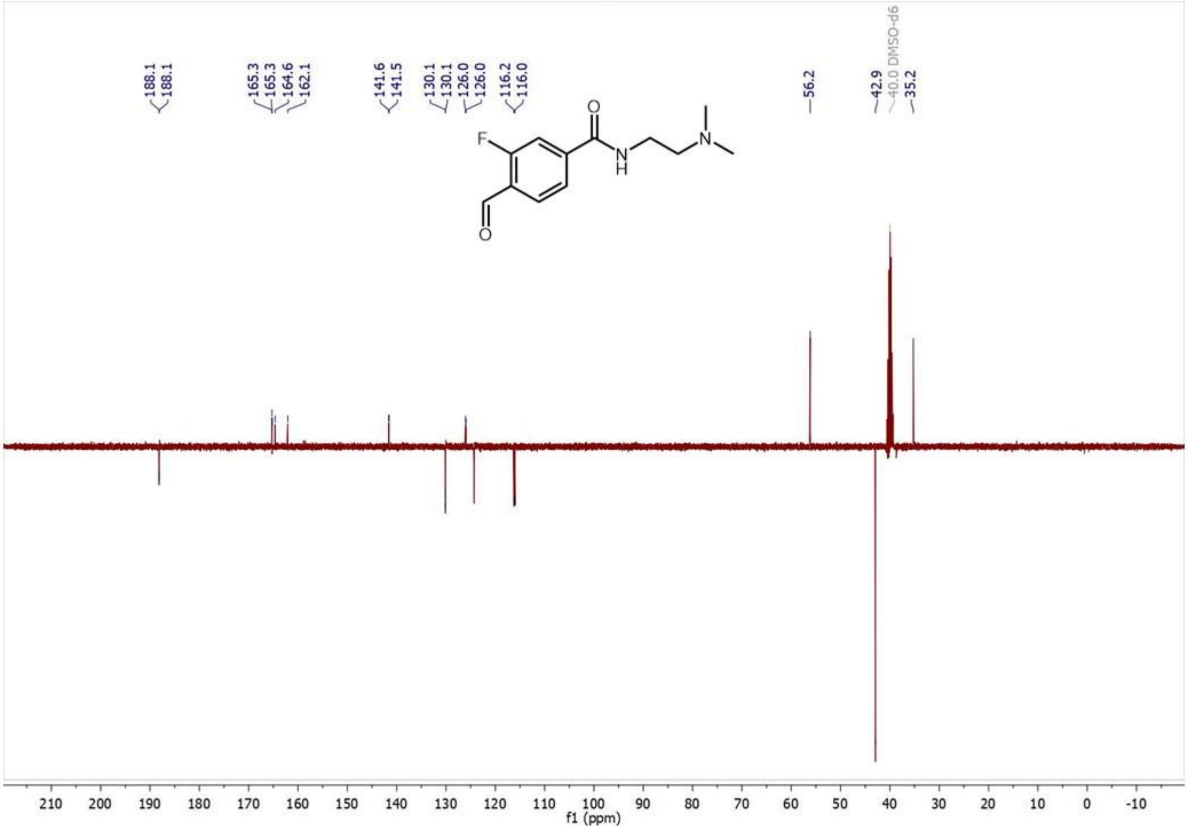

Compound 4 (RS1119)

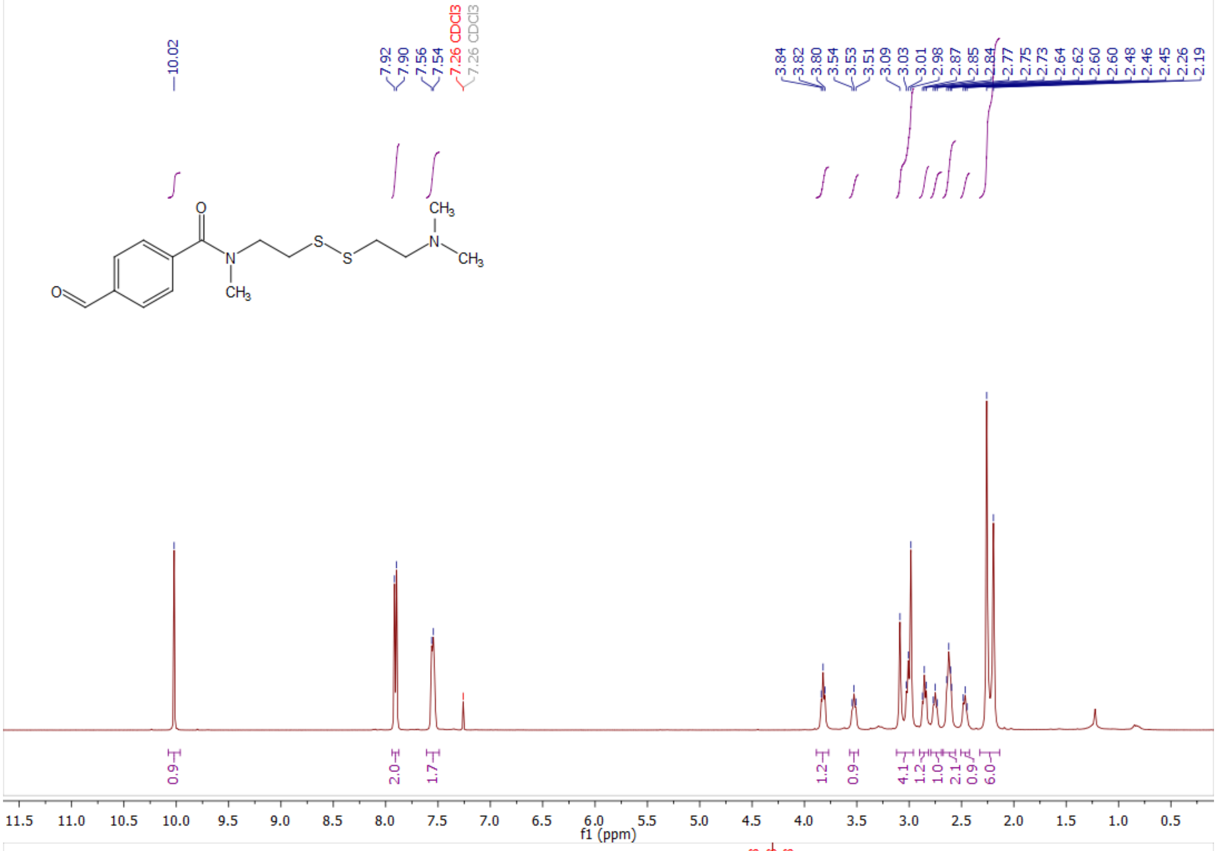

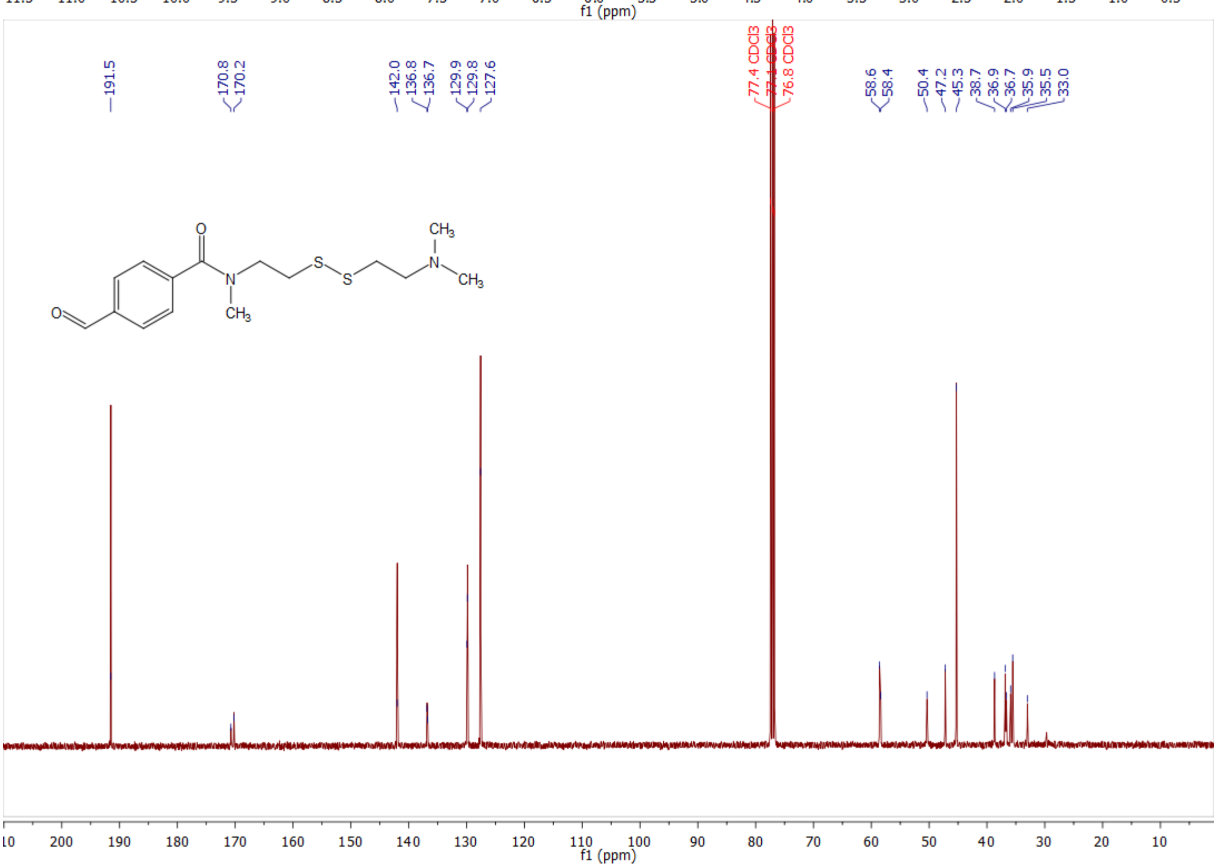

Compound 5 (RS1121)

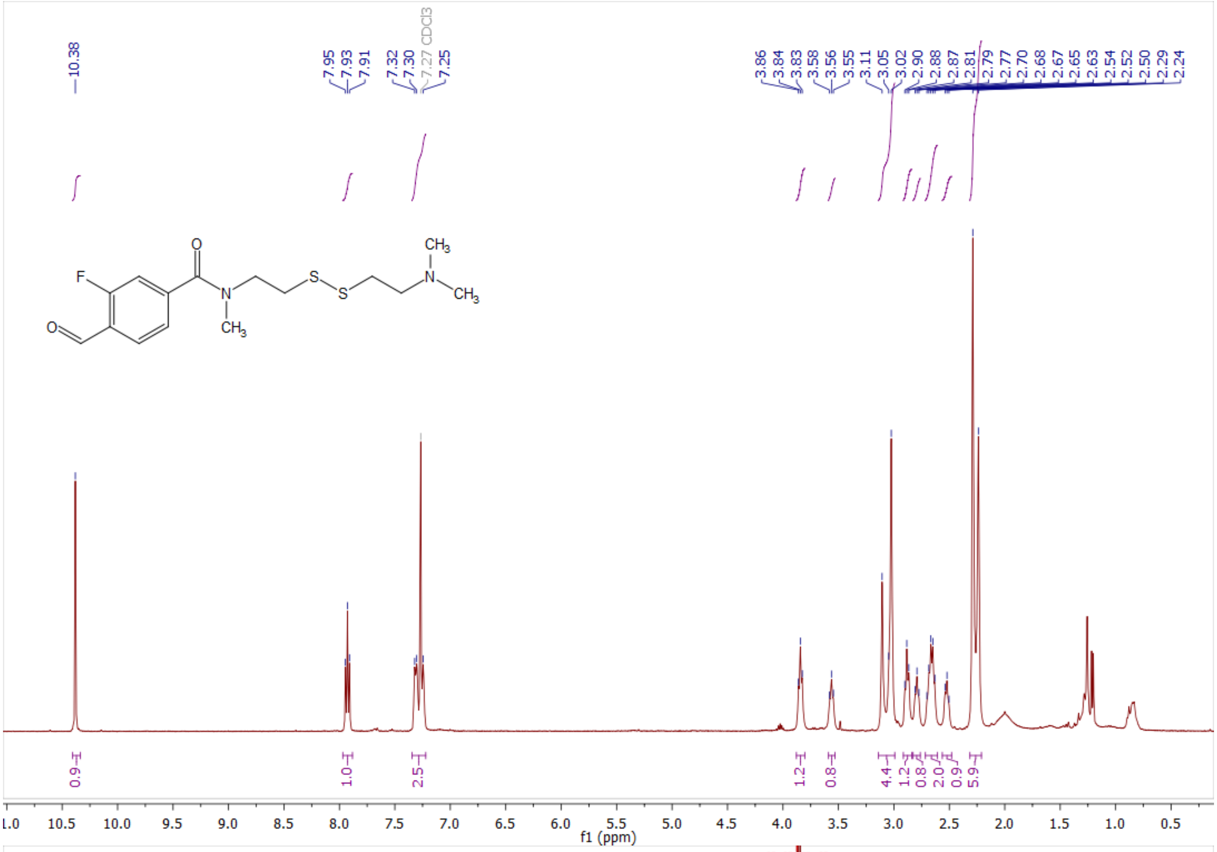

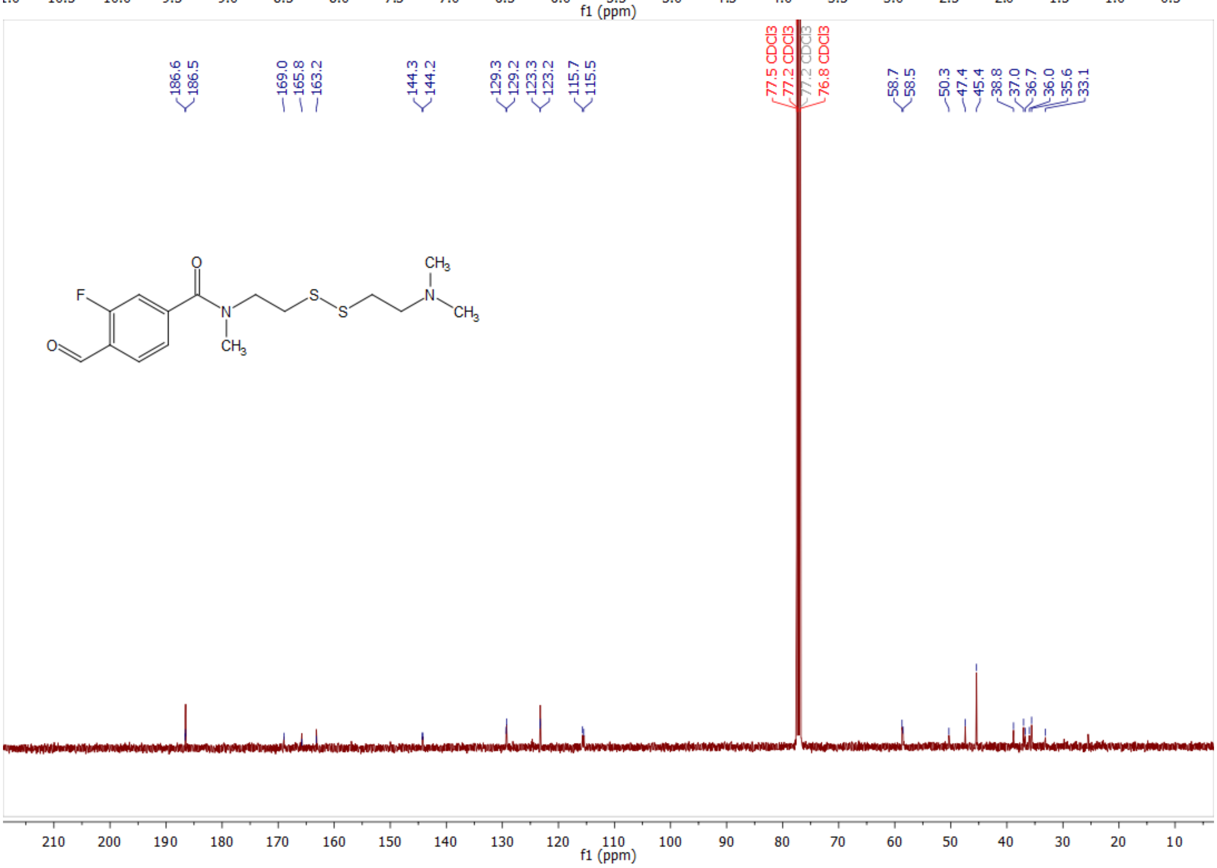

Compound 6 (PC21909A)

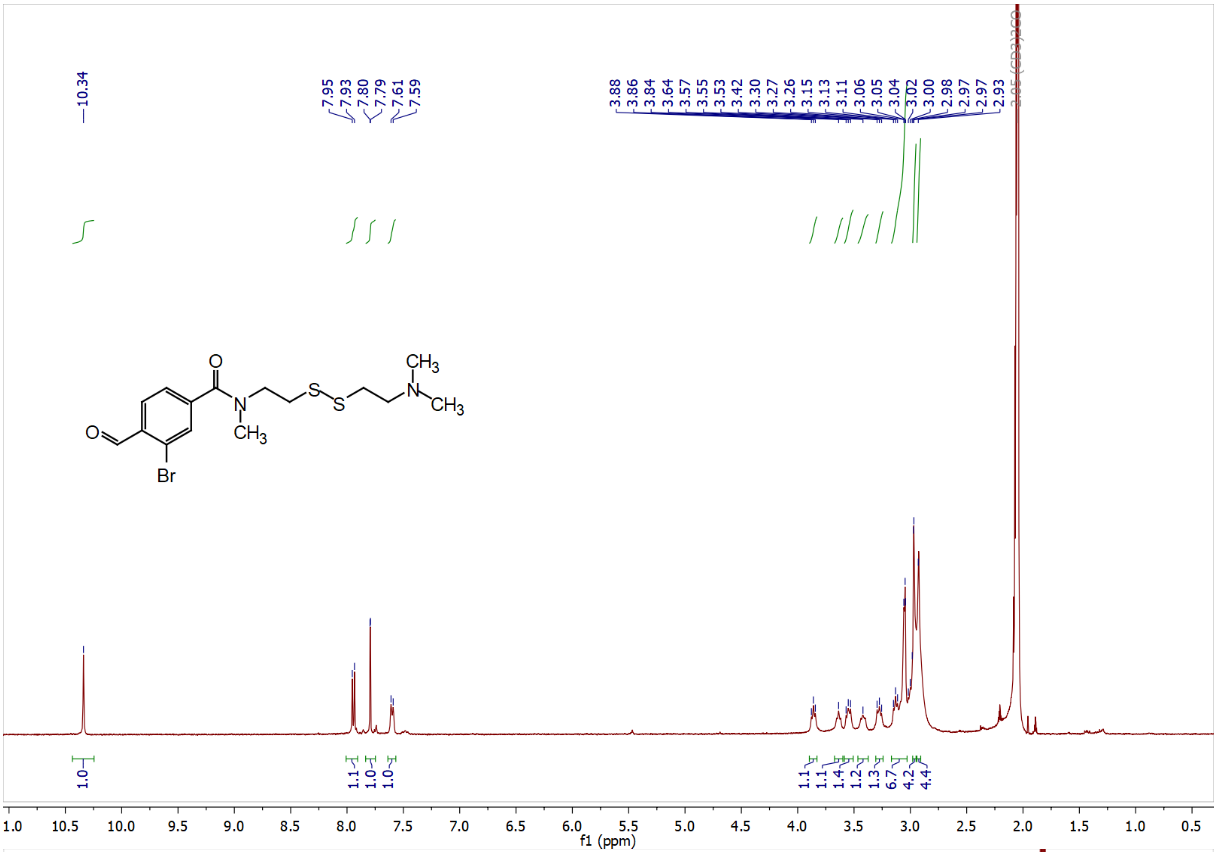

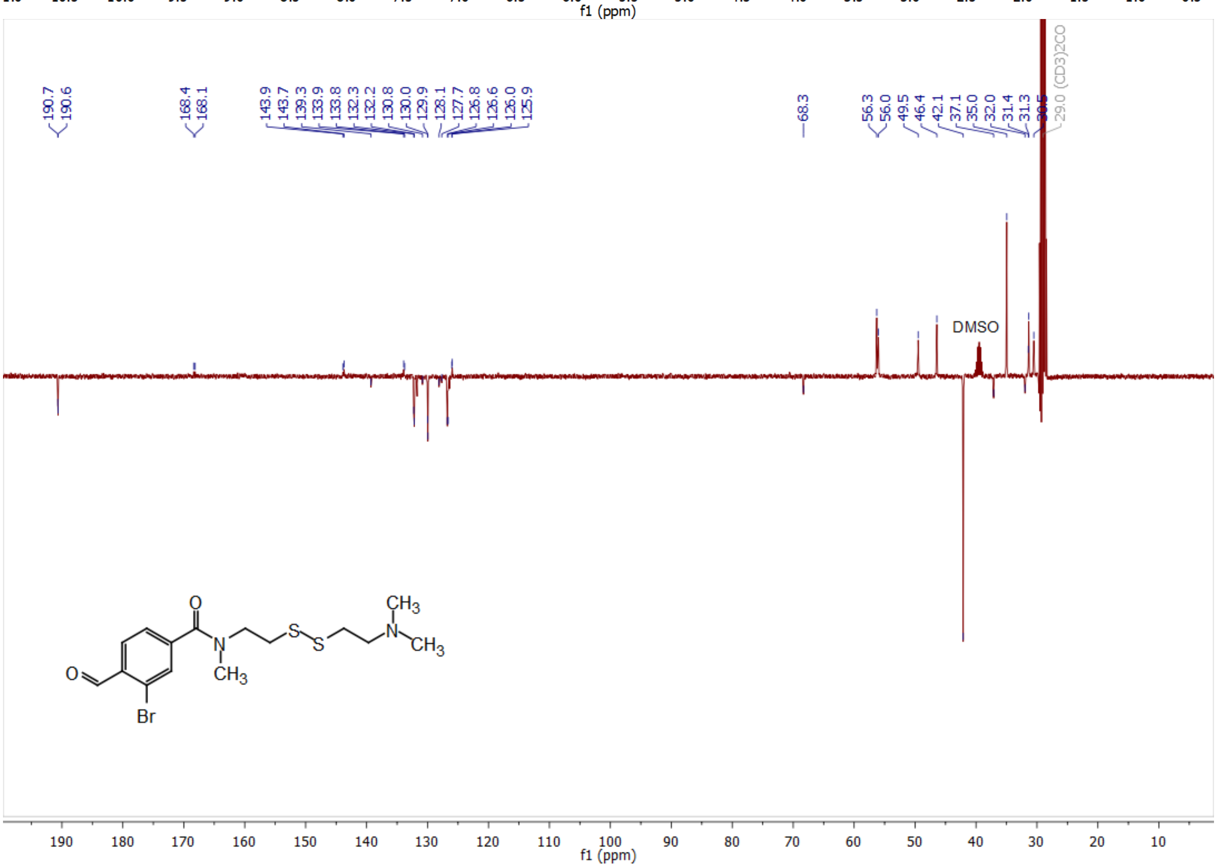

Compound 7 (RS1122)

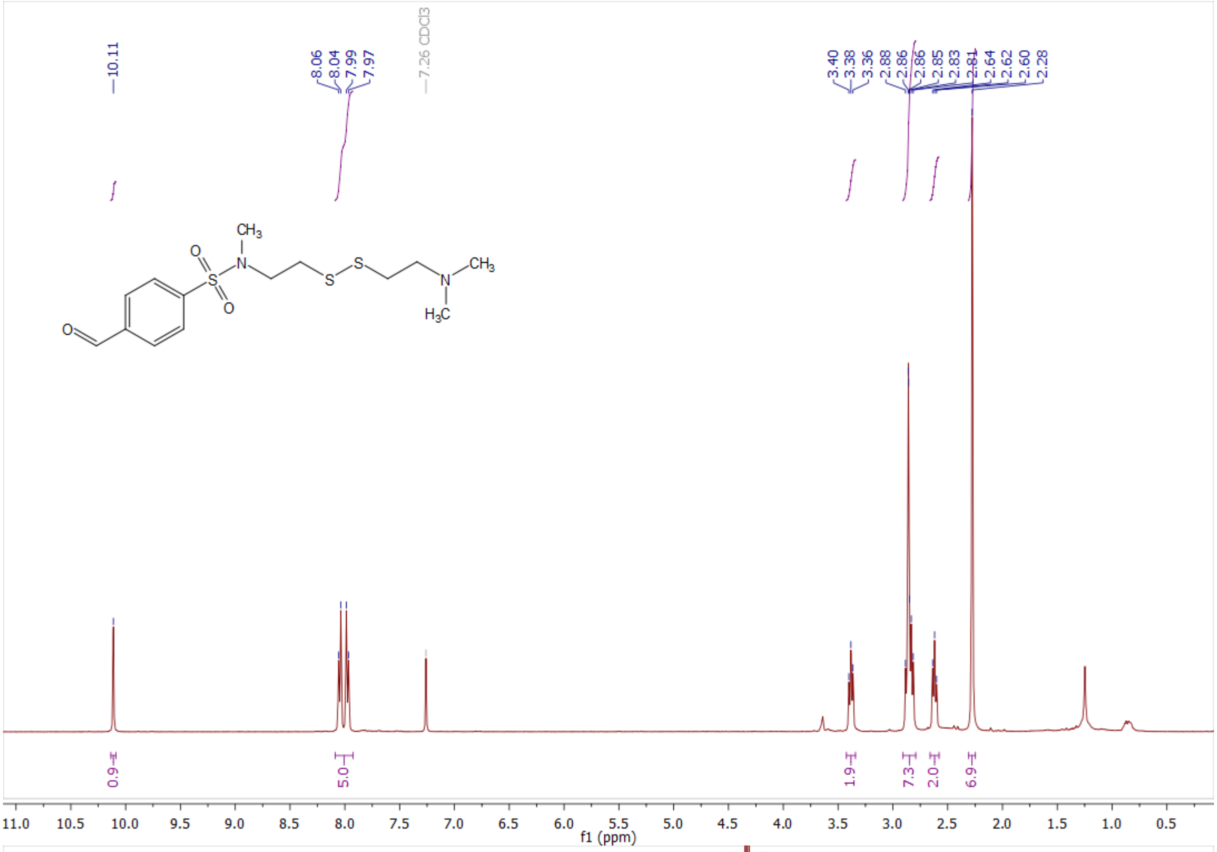

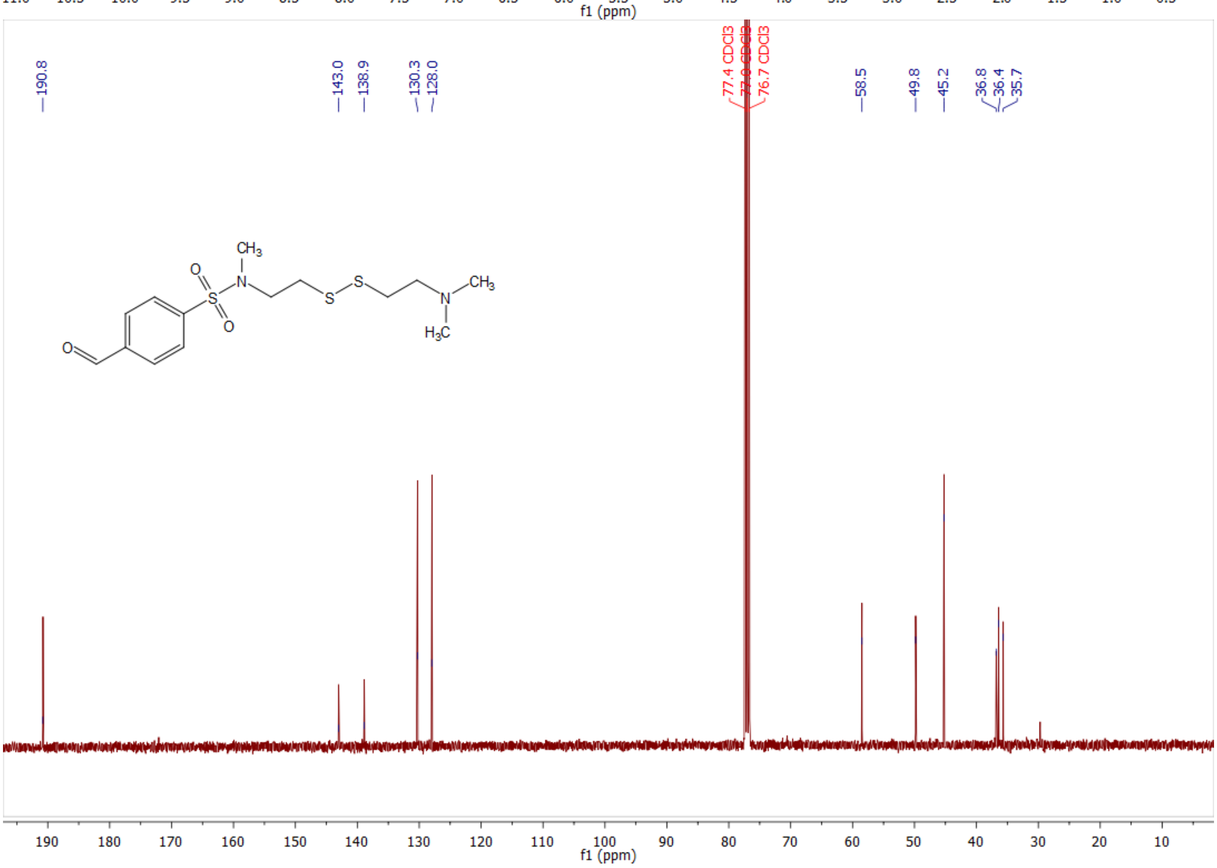

Compound 8 (RS1125)

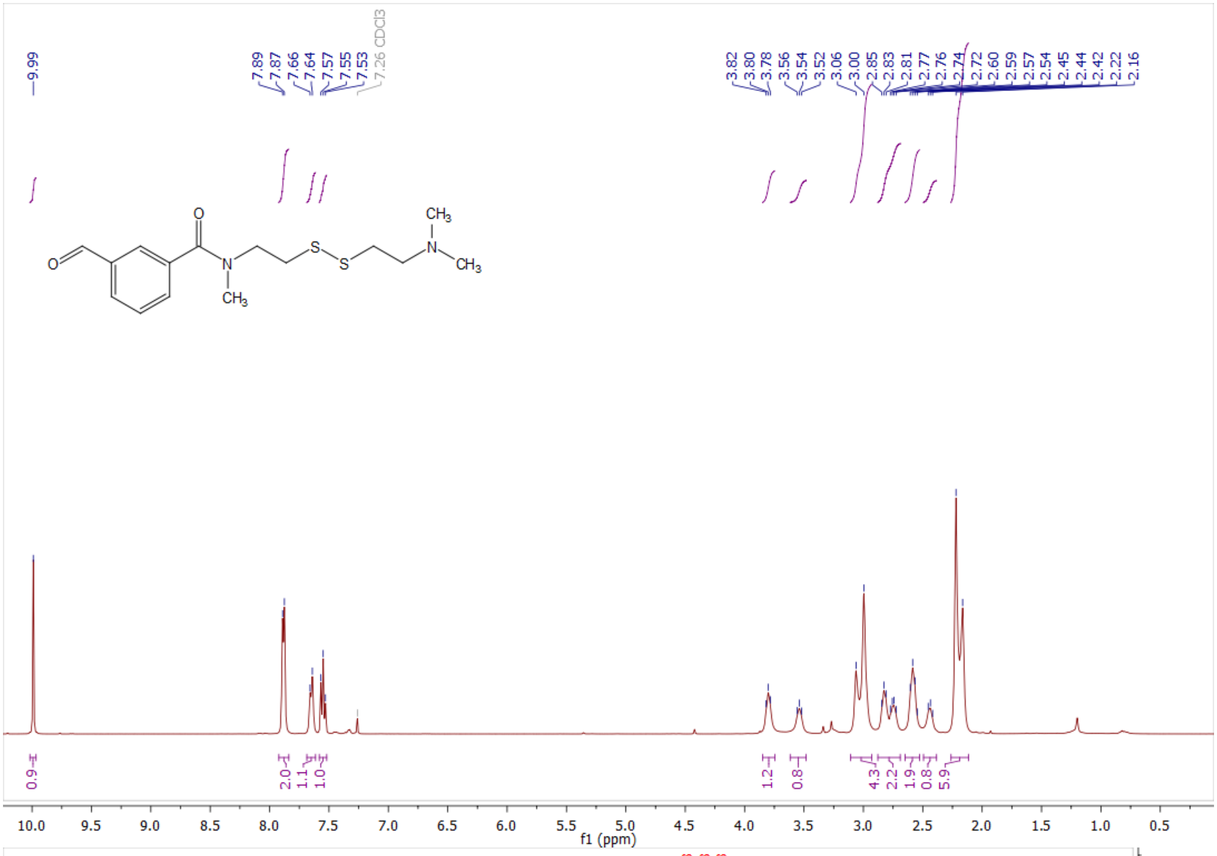

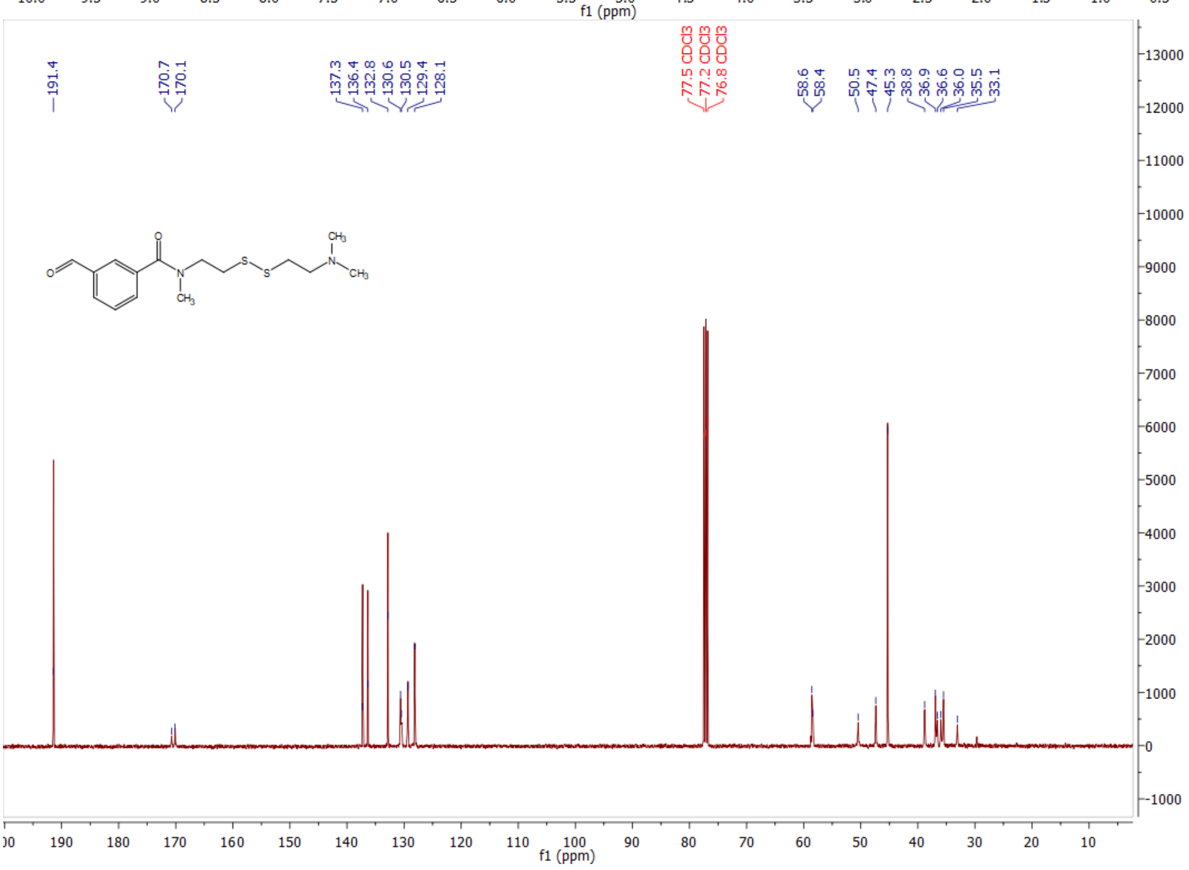

Compound 9 (PC917)

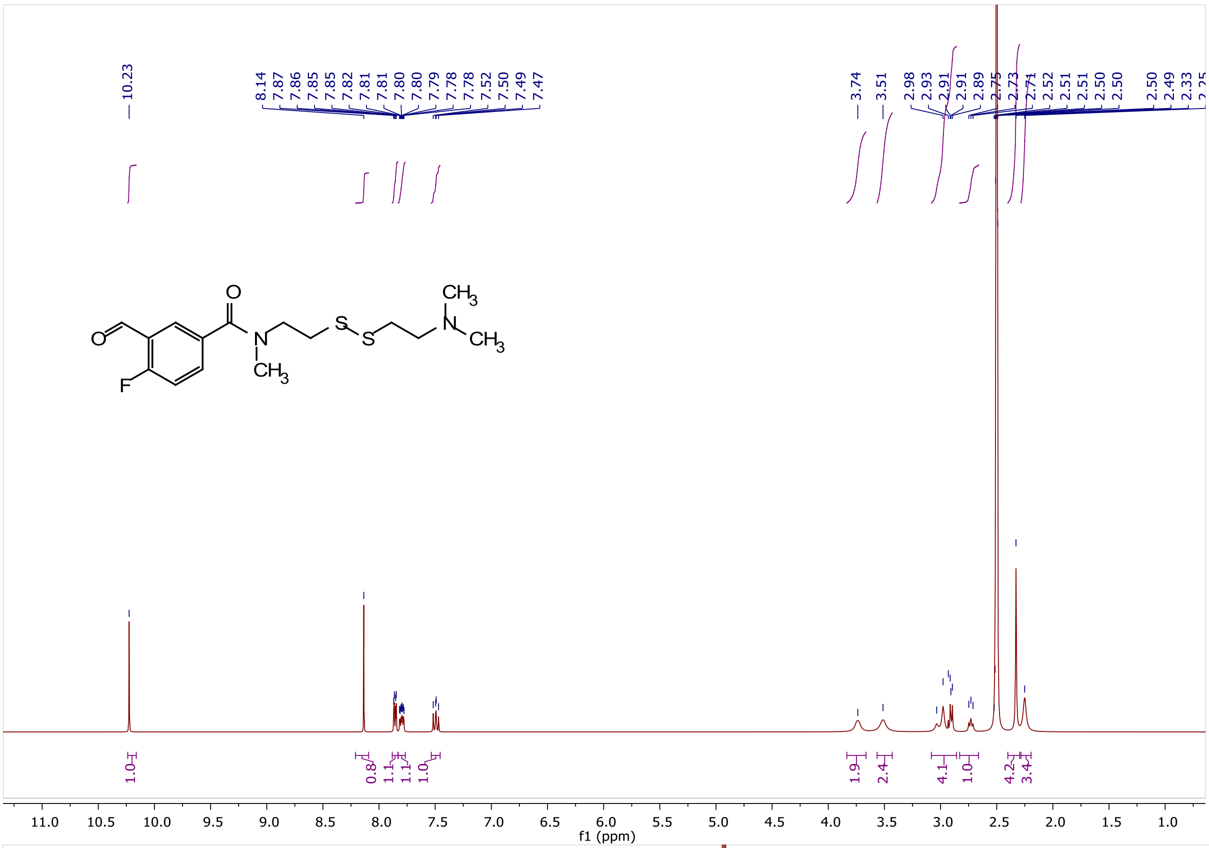

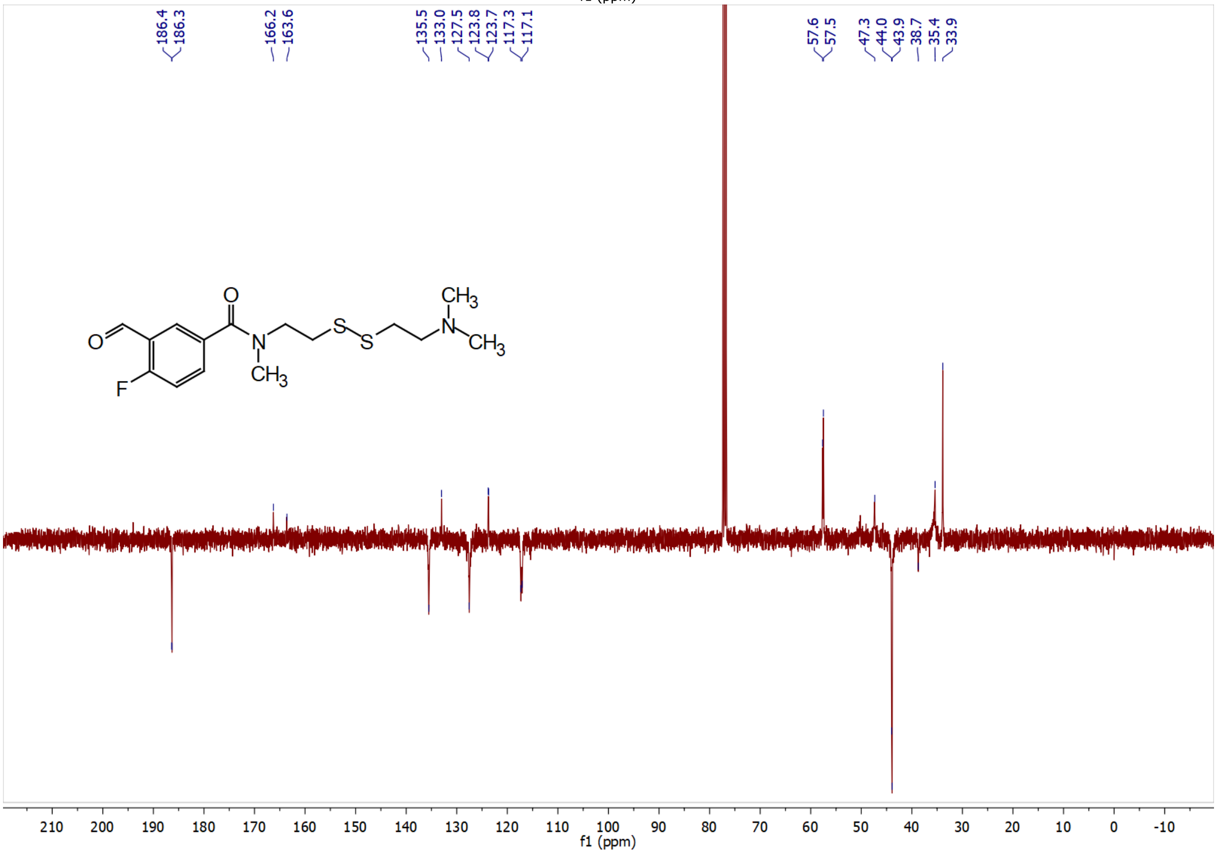

Compound 10 (PC909B)

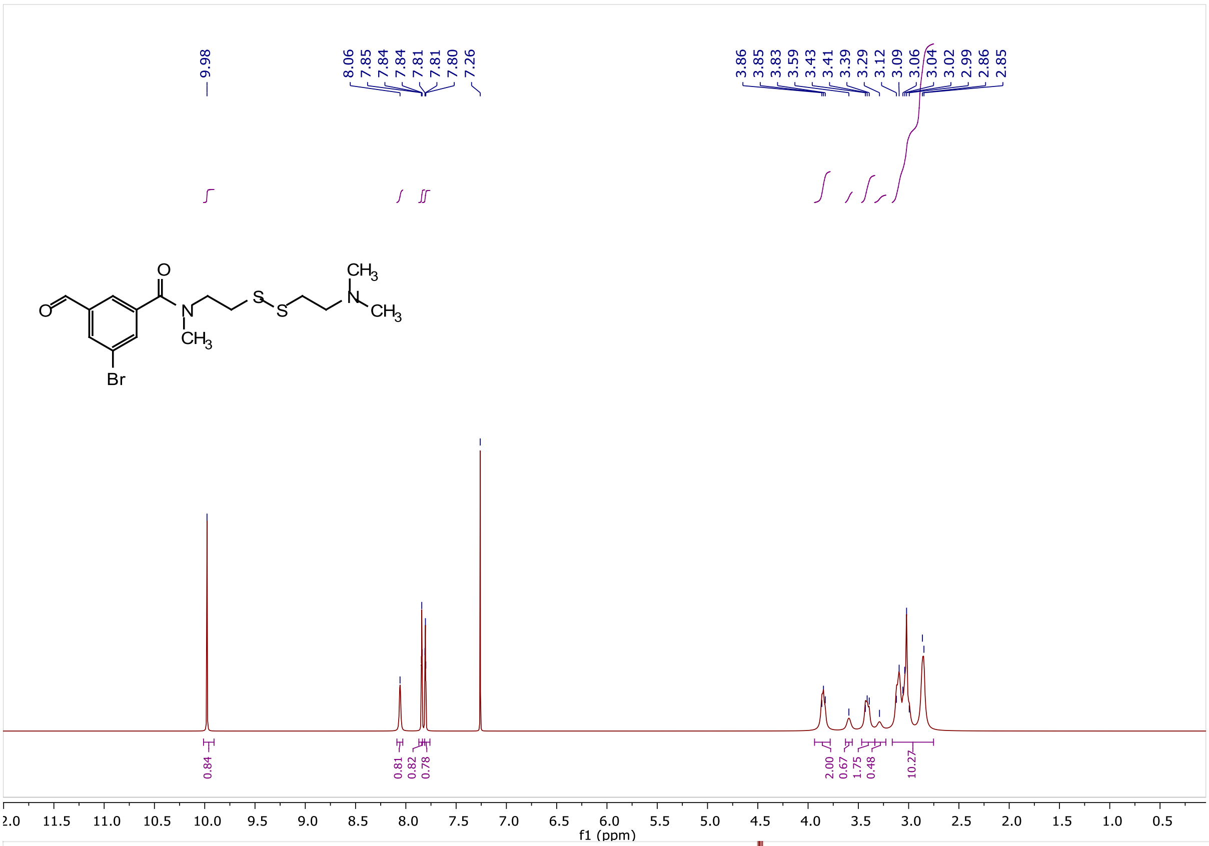

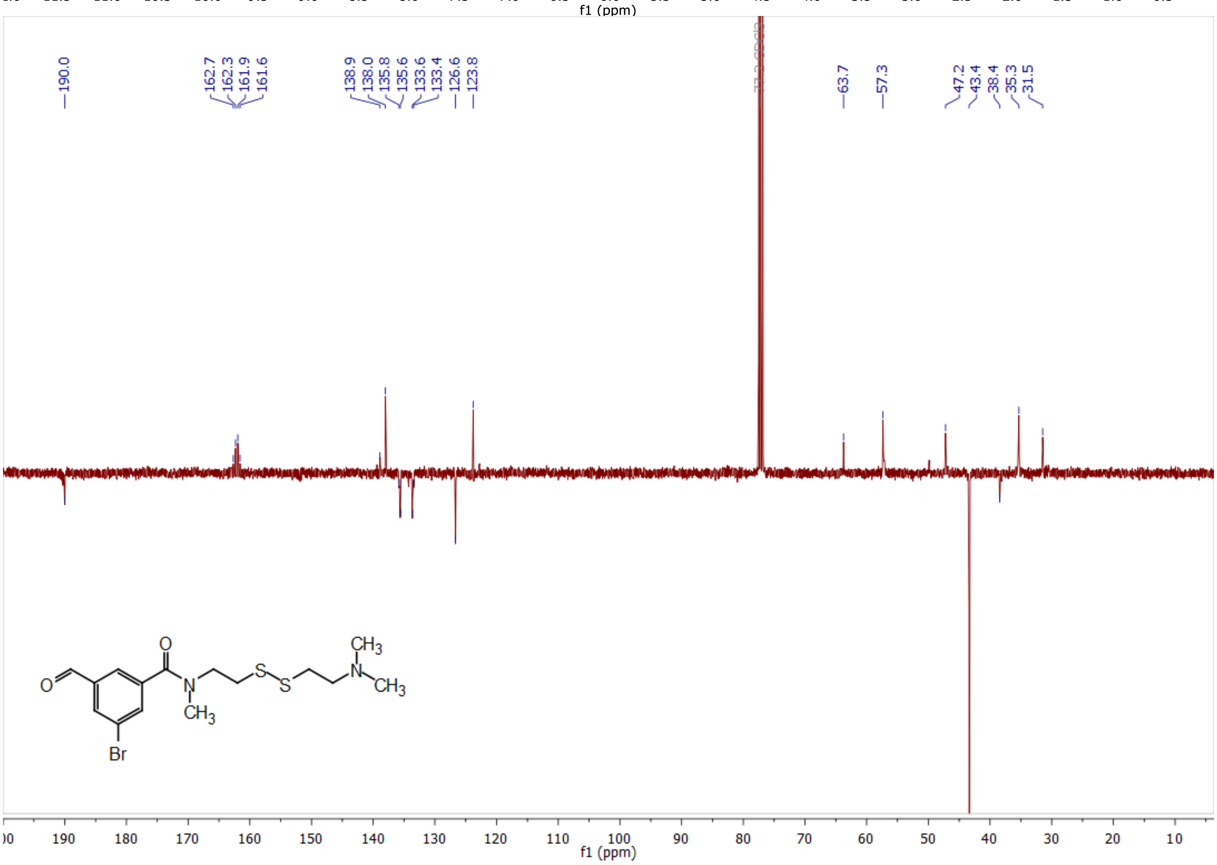

SI References

1. Geertjens, N. H. J.; de Vink, P. J.; Wezeman, T.; Markvoort, A. J.; Brunsveld, L. Straightforward model construction and analysis of multicomponent biomolecular systems in equilibrium. *RSC Chem. Biol.*, DOI: https://doi.org/10.1039/D2CB00211F (2023).
2. Potterton, L. et al. CCP4i2: the new graphical user interface to the CCP4 program suite. *Acta Crystallogr. Sect. D Struct. Biol*. 74, 68–84 (2018).
3. Clabbers, M. T. B. et al. Electron diffraction data processing with DIALS. *Acta Crystallogr. Sect. D Struct. Biol.* 74, 506–518 (2018).
4. Evans, P. R., Murshudov, G. N. How good are my data and what is the resolution? *Acta Crystallogr. D. Biol. Crystallogr.* 69, 1204-14 (2013).
5. Evans, P. R., An introduction to data reduction: space-group determination, scaling and intensity statistics. *Acta Crystallogr. D. Biol. Crystallogr.* 67, 282-292 (2011).
6. Vagin. A., Teplyakov. A.., Molecular replacement with MOLREP. *Acta Crystallogr. D. Biol. Crystallogr.* 66, 22-25 (2010).
7. Long, F. et al. AceDRG : a stereochemical description generator for ligands. *Acta Crystallogr. Sect. D Struct. Biol.* 73, 112–122 (2017).
8. Emsley, P., Lohkamp, B., Scott, W. G. & Cowtan, K. Features and development of Coot. Acta Crystallogr. Sect. D Biol. Crystallogr. 66, 486–501 (2010).
9. Murshudov, G. N. et al. REFMAC5 for the refinement of macromolecular crystal structures. Acta Crystallogr. D. Biol. Crystallogr. 67, 355–67 (2011).
10. Kovalevskiy, O. et al. Overview of refinement procedures within REFMAC5: utilizing data from different source. *Acta Crystallogr. Sect. D Biol. Crystallogr.* 74, 215-227 (2018).
11. Afonine, P. V. et al. Towards automated crystallographic structure refinement with phenix.refine. *Acta Crystallogr. Sect. D Biol. Crystallogr.* 68, 352–367 (2012).
12. Liebschner D., et al (2019). Macromolecular structure determination using X-rays, neutrons and electrons: recent developments in Phenix. Acta Cryst. D75, 861-877.
13. Schrodinger LLC. The PyMOL Molecular Graphics System, Version 2.2.3. (Schrödinger LLC, 2015)
